# Deciphering the distinct transcriptomic and gene regulatory map in adult macaque basal ganglia cells

**DOI:** 10.1093/gigascience/giad095

**Published:** 2023-12-13

**Authors:** Zihao Li, Yunong Sun, Lingjun Ding, Jing Yang, Jinrong Huang, Mengnan Cheng, Liang Wu, Zhenkun Zhuang, Cheng Chen, Yunqi Huang, Zhiyong Zhu, Siyuan Jiang, Fubaoqian Huang, Chunqing Wang, Shiping Liu, Longqi Liu, Ying Lei

**Affiliations:** College of Life Sciences, University of Chinese Academy of Sciences, Beijing 100049, China; BGI Research, Hangzhou 310030, China; College of Life Sciences, University of Chinese Academy of Sciences, Beijing 100049, China; BGI Research, Hangzhou 310030, China; BGI Research, Hangzhou 310030, China; BGI Research, Hangzhou 310030, China; BGI Research, Shenzhen 518083, China; BGI Research, Hangzhou 310030, China; BGI Research, Shenzhen 518083, China; BGI Research, Hangzhou 310030, China; College of Life Sciences, University of Chinese Academy of Sciences, Beijing 100049, China; BGI Research, Hangzhou 310030, China; College of Life Sciences, University of Chinese Academy of Sciences, Beijing 100049, China; BGI Research, Hangzhou 310030, China; College of Life Sciences, University of Chinese Academy of Sciences, Beijing 100049, China; BGI Research, Hangzhou 310030, China; College of Life Sciences, University of Chinese Academy of Sciences, Beijing 100049, China; BGI Research, Hangzhou 310030, China; BGI Research, Hangzhou 310030, China; School of Biology and Biological Engineering, South China University of Technology, Guangzhou 510006, China; College of Life Sciences, University of Chinese Academy of Sciences, Beijing 100049, China; BGI Research, Shenzhen 518083, China; BGI Research, Hangzhou 310030, China; BGI Research, Shenzhen 518083, China; College of Life Sciences, University of Chinese Academy of Sciences, Beijing 100049, China; BGI Research, Hangzhou 310030, China; BGI Research, Shenzhen 518083, China; BGI Research, Shenzhen 518083, China

**Keywords:** basal ganglia, single cell, snATAC-seq, snRNA-seq

## Abstract

**Background:**

The basal ganglia are a complex of interconnected subcortical structures located beneath the mammalian cerebral cortex. The degeneration of dopaminergic neurons in the basal ganglia is the primary pathological feature of Parkinson's disease. Due to a lack of integrated analysis of multiomics datasets across multiple basal ganglia brain regions, very little is known about the regulatory mechanisms of this area.

**Findings:**

We utilized high-throughput transcriptomic and epigenomic analysis to profile over 270,000 single-nucleus cells to create a cellular atlas of the basal ganglia, characterizing the cellular composition of 4 regions of basal ganglia in adult macaque brain, including the striatum, substantia nigra (SN), globus pallidum, and amygdala. We found a distinct epigenetic regulation on gene expression of neuronal and nonneuronal cells across regions in basal ganglia. We identified a cluster of SN-specific astrocytes associated with neurodegenerative diseases and further explored the conserved and primate-specific transcriptomics in SN cell types across human, macaque, and mouse. Finally, we integrated our epigenetic landscape of basal ganglia cells with human disease heritability and identified a regulatory module consisting of candidate *cis*-regulatory elements that are specific to medium spiny neurons and associated with schizophrenia.

**Conclusions:**

In general, our macaque basal ganglia atlas provides valuable insights into the comprehensive transcriptome and epigenome of the most important and populous cell populations in the macaque basal ganglia. We have identified 49 cell types based on transcriptomic profiles and 47 cell types based on epigenomic profiles, some of which exhibit region specificity, and characterized the molecular relationships underlying these brain regions.

## Introduction

The basal ganglia and related nuclei are critical for motor control, motor learning, cognitive functions, and emotional response [1]. The heterogeneous neuron distribution is underlying these diversities of physiological functions, exemplified by the best-known medium spiny neurons in striatum and dopaminergic neurons in the substantia nigra area. The functional organization of basal ganglia, especially the motor circuit, is tightly related to neurodegenerative diseases, such as Parkinson's disease [2]. Single-cell technology has dissected the cell taxonomy of basal ganglia and related areas in mouse and primate [3, 4]. Single-cell technologies have shown significance in assessing cell type–specific gene expression differences in several brain diseases, including Alzheimer's disease, autism spectrum disorder (ASD), multiple sclerosis, and major depressive disorder (MDD) [5–8]. Identifying cell type–specific gene expression is crucial for associating cell identities and functions, as well as for associating/linking cell types and the genetic variation underlying psychiatric disorders such as schizophrenia (SCZ) [9]. While substantial progress has been made in understanding cell type heterogeneity across and within different regions of the basal ganglia, most reports have been limited to single transcriptomics in a few brain regions, such as the substantia nigra (SN) [10], amygdala (AMY) [3], and striatum (STR) [11]. However, the subregional heterogeneities in cell types, transcriptomics, and epigenomics in basal ganglia have not been illustrated. Moreover, the regulatory state for specific cell types in basal ganglia and their correlation with neurological diseases still have not been well defined. It is important to note that the regulatory mechanisms behind the different cell types in different regions of the basal ganglia are vastly different [12]; genome-wide chromatin accessibility sequencing is more sensitive in assessing *cis*-regulatory elements and disease-associated genetic risk loci [13] compared to single-gene expression measurements. The specific cell types and regulatory mechanisms in different regions of the basal ganglia, as well as the association between cell types and disease risk, are still not well understood at present.

To better understand the gene regulatory landscape of nonhuman primate basal ganglia, tissues of the basal ganglia regions (including SN, AMY, STR, and globus pallidum [GP]) were sampled from two 72-month-old female macaques (*Macaca fascicularis*), followed by single-nucleus RNA sequencing (snRNA-seq) and single-nucleus ATAC sequencing (snATAC-seq). We defined 49 cell types by snRNA-seq and 47 cell types by snATAC-seq within the basal ganglia regions, uncovered their molecular features, and revealed the regulatory elements underlying the differences in gene expression among the cell types. We discovered region-specific subtypes of neurons and elucidated the heterogeneity of gene expression and regulatory mechanisms among these region-specific neurons. Additionally, we identified a group of SN-specific astrocyte subtypes associated with neurodegenerative diseases and revealed their transcriptional signatures. Simultaneously, we predicted the regulatory patterns of transcription factors significantly activated in these astrocyte subtypes on neurodegenerative disease-related genes. Furthermore, we systematically analyzed the cross-species conservation and primate-specific differentially expressed genes (DEGs) across different cell types of the SN region and revealed a correlation between primate-specific DEGs and neurodegenerative diseases. Finally, we identified cell type–specific chromatin-accessible sites colocalized with human trait–associated single-nucleotide polymorphisms and plotted disease-associated open regions and gene enrichment related to human neurological diseases in the nonhuman primate basal ganglia on a topographic map. Overall, our results provide a systematic analysis of the lineage of cellular composition, transcription, and regulation in various regions of the macaque basal ganglia. This study fills a gap in the basal ganglia epigenetic data and significantly expands our current understanding of the molecular basis of basal ganglia cell types. Comprehensive analysis of single-cell basal ganglia epigenomic data can help to understand the regulatory mechanisms of key genes in different cell types, study the cell type preference of disease risk loci, and facilitate the identification of therapeutic targets for diseases. The basal ganglia cell atlas presented here offers a valuable resource for future research on model species.

## Results

### Single-nucleus transcriptional and chromatin accessibility profiling in macaque basal ganglia

**Figure 1:**
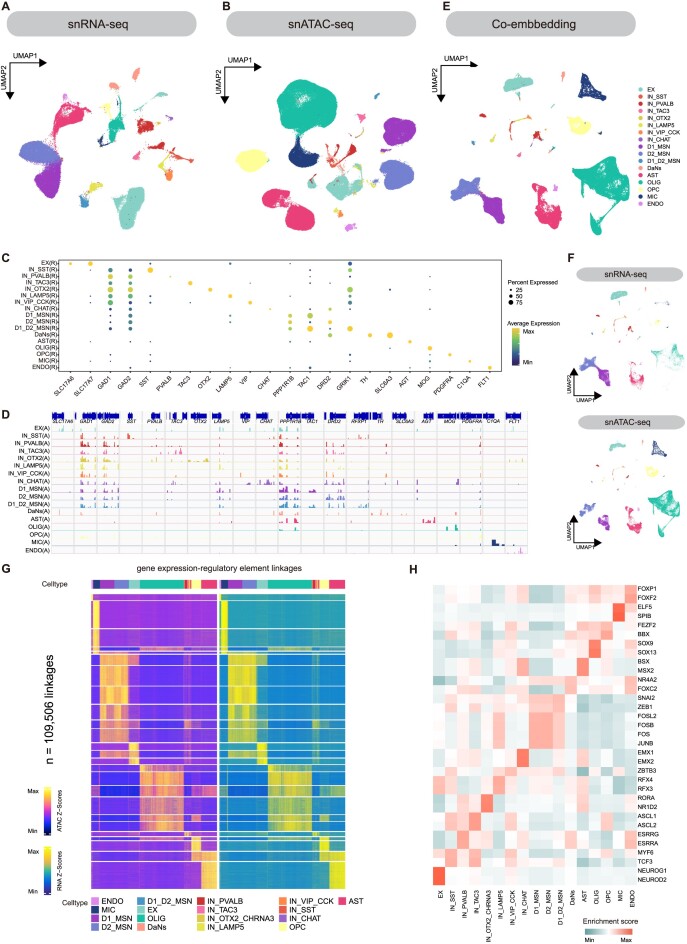
Single-cell transcriptomic and epigenomic characterization of cellular diversity in the basal ganglia. (A, B) UMAP plot showing the clustering of 101,431 nuclei from snRNA-seq (A) and 170,608 nuclei from snATAC-seq (B). Each point represents a nucleus and the colors indicate different cell types. There are 52 cell subtypes in snRNA-seq and 50 cell subtypes in snATAC-seq. (C) Expression of cell type marker genes for the main cell types is shown. The color represents the expression level and the size represents the percentage expression value. (D) Integrative Genomics Viewer (IGV) plot showing the read density of cell type–specific marker genes on snATAC-seq cell types in C. (E) UMAP plot showing the low-dimensional co-embedding clustering of snRNA-seq and snATAC-seq. The main cell types are marked with different colors. (F) Integrated UMAP and separated by data type. (G) Heatmap showing the chromatin accessibility and gene expression of 109,506 significantly linked cRE–gene pairs. It is represented by a one-to-one heatmap with cRE activity on the left and its linked gene expression value on the right. cREs and genes can link to each other mutually. Hence, each cRE and gene may appear repeatedly in the corresponding rows of the heatmap. The cRE–gene link is clustered by *k*-means (*k* = 25). (H) Heatmap showing the motif activity of TFs in different cell types in snATAC-seq.

The basal ganglia striatal caudate (Cd), putamen (Pu), AMY, SN, and GP of 2 female macaque were obtained for snRNA-seq and snATAC-seq sequencing, and the transcriptomic and epigenomic data were generated from the same tissue. After quality control filtering, we obtained a total of 101,431 nuclei for snRNA-seq (20,104 from Pu, 36,451 from Cd, 31,219 from AMY, 9,386 from SN, and 4,271 from GP) (Methods, Supplementary Fig. S1A, B, and F and Supplementary Table S1) and 170,608 nuclei for snATAC-seq (42,027 from Pu, 41,026 from Cd, 20,995 from AMY, 24,221 from SN, and 42,339 from GP) (Methods, Supplementary Fig. S1C, D, and F and Supplementary Table S1). To minimize differences between sample sources in snRNA-seq, we performed batch correction between samples. We then used uniform manifold approximation and projection (UMAP) to reduce the dimensionality of the snRNA-seq data. Subsequently, we employed original Louvain clustering to identify unique clusters of cell types (Fig. 1A and Supplementary Fig. S1E). Based on the expression of marker genes for different cell types in previous studies, we have defined 17 major cell types and 49 subtypes [4, 10, 14–16]. These include excitatory neurons (EX, *SLC17A6*^+^/*SLC17A7*^+^), inhibitory neurons (IN, *GAD1*^+^/*GAD2*^+^, IN_SST interneurons [*SST*^+^], IN_PVALB interneurons [*PVALB*^+^], IN_TAC3 interneurons [*TAC3*^+^], IN_OTX2 interneurons [*OTX2*^+^/*CHRNA3*^+^], IN_LAMP5 interneurons [*LAMP5*^+^], IN_VIP_CCK interneurons [*VIP*^+^], IN_CHAT interneurons [*CHAT*^+^]), medium spiny neurons (MSN, *PPP1R1B*^+^), dopaminergic neurons (DaNs, *TH*^+^), astrocytes (AST, *AGT*^+^), oligodendrocytes (OLIG, *MOG*^+^), oligodendrocyte precursor cells (OPC, *PDGFRA*^+^), microglia (MIC, *C1QA*^+^), and endothelial cells (ENDO, *FLT1*^+^) (Fig. 1C).

For snATAC-seq, we processed the data using the ArchR software package [17] to obtain a low-dimensional result through an iterative approach. Then a consensus set of 657,930 accessible peaks representing potential *cis*-regulatory elements (cREs) based on preliminary clustering results was obtained. To ensure consistency between the 2 technical sources in cell types, we extracted peak and gene score matrices and used SeuratV4 to establish anchors between cells from different technical sources by gene score and gene expression matrices, then integrated the 2 types of dataset by mapping snATAC-seq cells to the low-dimensional space of snRNA-seq (Fig. 1E, F). We annotated the major cell types for snATAC-seq using the labels from snRNA-seq results excluding the cells with prediction scores below 0.6. We utilized the Signac package to perform UMAP reduction and batch correction on the data, followed by the utilization of smart local moving clustering for analyzing the snATAC-seq datasets (Fig. 1B and Supplementary Fig. S1E). The predicted cell types were validated by the increased accessibility in the promoter of marker genes for the corresponding major cell types (Fig. 1D).

**Figure 2:**
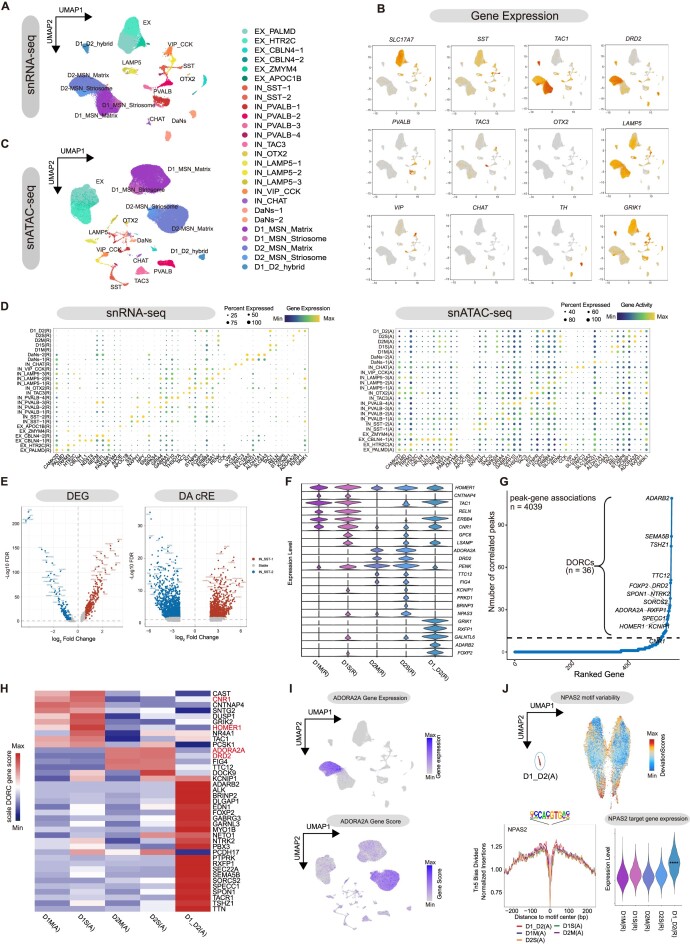
Transcriptional regulation heterogeneity of neuron type–specific and region-specific genes in the basal ganglia. (A) UMAP plot displays the subclassification of basal ganglia neurons from snRNA-seq, with colors representing different subtypes of cells. (B) UMAP plot showing the expression of classical marker genes for neurons in snRNA-seq data. (C) UMAP plot displays the subclassification of basal ganglia neurons from snATAC-seq, with colors representing different subtypes of cells. (D) Dot plot illustrating the expression (left) and activity (right) patterns of DEGs in neuronal subtypes and novel markers identified here. (E) Volcano plot representing the DEGs between IN_SST subtypes from snRNA-seq (left) and the DA cREs (|Log2 fold change (FC)| > 1.5 and false discover rate (FDR) < 0.01) between IN_SST subtypes from snATAC-seq (right), where each point represents a gene or a DA cRE, respectively. The points marked with corresponding subtypes indicate the DEGs or DA cREs linked to them. (F) Violin plots showing MSN subtype-specific marker gene expression. (G) The number of significantly correlated peaks (FDR < 0.01) for each MSN subtype DEG (FDR < 0.01). (H) The gene score of DORCs in subtypes of MSN, with red indicating downregulated genes in patients with Huntington's disease. (I) The gene expression of *ADORA2A* (upper panel) and *ADORA2A* gene score (lower panel) colored in UMAP plot. (J) The upper panel shows the UMAP plot of the snATAC-seq MSN recluster colored by NPAS2 motif variability, while the lower panel displays Tn5 bias-subtracted TF footprinting for NPAS2 by the snATAC-seq MSN subcluster (left) and NPAS2 target gene expression in the MSN subcluster (right).

**Figure 3:**
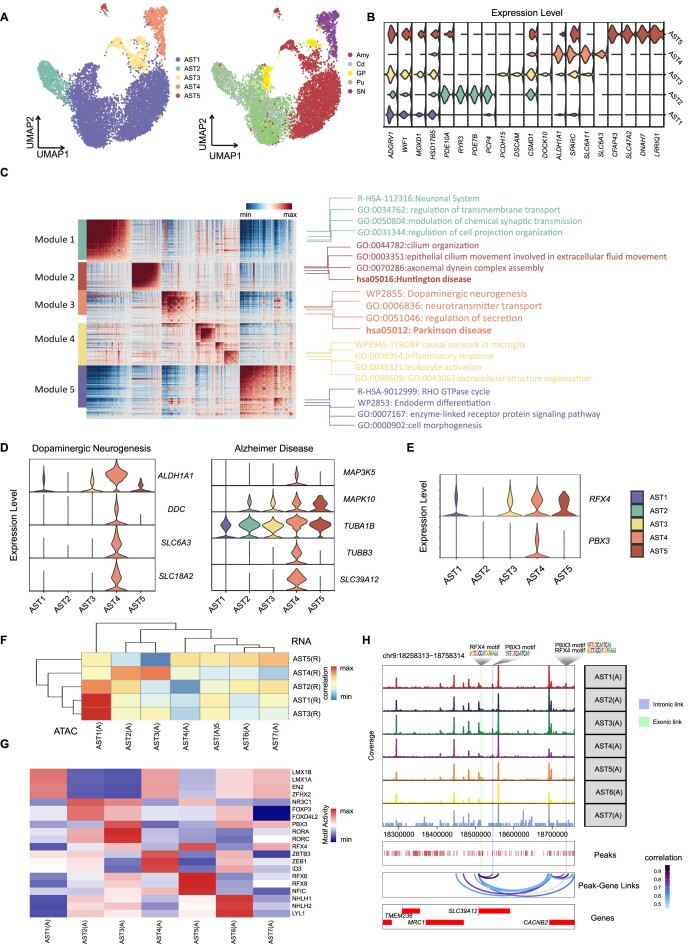
Substantia nigra–specific astrocyte subpopulations. (A) UMAP plot representing subtypes of ASTs from snRNA-seq, where colors represent different cell types (left) and their regional distribution (right). (B) The violin plot shows the differential gene expression patterns of the AST subtypes in snRNA-seq. (C) In total, 2,404 significantly auto-correlated genes (FDR <0.05) were clustered into 5 modules based on pairwise correlation. (D) Violin plot showing the enrichment of genes related to dopaminergic neurogenesis and Alzheimer's disease in the AST4 subtype. (E) The violin plot shows the expression patterns of selected transcription factors with specific expression in AST4 across AST subtypes. (F) Heatmap displaying the correlation between AST subtypes from snRNA-seq and snATAC-seq, revealing a correlation between AST4 from snRNA-seq and AST_c from snATAC-seq. (G) Heatmap showing the enrichment of transcription factor motifs in AST cell subtypes from snATAC-seq. (H) Visualization of the predicted peak–gene pairs containing transcription factor motif binding sites for RFX4 and PBX3 in the locus of the Alzheimer's disease–related gene *SLC39A12*.

Next, we performed an unsupervised clustering analysis of major cell types in snRNA-seq and snATAC-seq data. In total, 49 subtypes were revealed by snRNA-seq and 47 subtypes were revealed by snATAC-seq based on differences in marker gene expression or chromatin accessibility (Supplementary Fig. S1A and B, Figs. 2 and 3, Supplementary Figs. S3 and S4). We further identified a differential accessible cRE (DA cRE) set for the snATAC-seq subtypes. We found that the differences between neuronal subtypes were greater than those between nonneuronal subtypes (Supplementary Fig. S1G). Using ArchR, we linked distal cRE accessibility to gene expression to identify 109,506 cRE–gene pairs representing potential enhancer–gene interactions (Methods, Supplementary Table S3). The covariation of cRE accessibility and gene expression distinguished cell types identified in snRNA-seq and snATAC-seq (Fig. 1G). Clustering of cRE accessibility revealed cell type–specific variability, confirming the similarity of neuronal subclusters and heterogeneity across neuronal and nonneuronal subtypes, as well as indicating dynamic modes of gene regulation across inhibitory neuron clusters.

Next, we used Chromvar to calculate the enrichment level of transcription factor (TF) binding motifs in chromatin open regions of each snATAC-seq cell and evaluated the cell type enrichment of TF binding motifs (Fig. 1H). The TFs with enriched binding motif in these cell types are functionally related to their respective cell types. For example, NEUROD2, enriched in EX, is an effector TF expressed in the cortical projection neuron lineage during the peak of cortical excitatory neurogenesis [18] and is crucial for the development of the AMY [19]. JUNB and FOS families, enriched in MSN, are associated with MSN desensitization [20]. EMX1 and EMX2 are enriched in IN_CHAT, and in the absence of EMX2 and PAX6, EMX1 might inhibit basal ganglia morphogenesis [21]. The transcription factors ASCL2 and TCF3, which are enriched in the IN_PVALB, IN_SST, and IN_TAC cell types, are involved in the development, proliferation, and differentiation of neurons and neural progenitor cells [22, 23], suggesting a crucial role in controlling the formation and function of these specific types of inhibitory neurons. The AST cell type is enriched with the MSX2 binding motif, where this TF is expressed in a time-dependent manner in glial cells after injury [24], while the OPC-enriched BBX may be involved in central nervous system development [25], and SOX9 regulates oligodendrocyte differentiation [26]. It has been suggested that SPIB TF may be an important regulatory factor for MIC sensing function [27], and FOXF2 has been shown to be involved in the development and maintenance of the ENDO blood–brain barrier [28]. It is worth noting that NR4A2 is highly expressed in DaNs. The expression of nr4a2 is crucial for the early differentiation of dopamine neurons, and its expression in adulthood is necessary for dopamine to carry out its function [29].

### Heterogeneity of gene expression and transcriptional regulation in basal ganglia neurons

Based on the gene expression heterogeneity among neurons, we further investigated the feature of neuron subtypes in basal ganglia. The 83,206 neuronal cells revealed by snRNA-seq were categorized into 26 subtypes, as shown in Fig. 2A, B, and D. These comprise 6 subtypes of excitatory neurons, namely, EX_PALMD, EX_HTR2C, EX_CBLN4 (including 2 subtypes, EX_CBLN4-1 and EX_CBLN4-2), EX_ZMYM4, and EX_APOC1B. Additionally, there are 13 subtypes of inhibitory neurons: IN_SST (IN_SST-1 and IN_SST-2), IN_PVALB (IN_PVALB-1-4), IN_TAC3, IN_OTX2_CHRNA3, IN_LAMP5 (IN_LAMP5-1-3), IN_VIP_CCK, and cholinergic neurons (IN_CHAT). Moreover, the data also revealed the presence of 2 subtypes of dopaminergic neurons (DaNs, which included DaNs-1 and DaNs-2) and 5 subtypes of medium spiny neurons D1_MSN (D1_MSN_Matrix and D1_MSN_Striosome), D2_MSN (D2_MSN_Matrix and D2_MSN_Striosome), and D1_D2_hybrid MSN.

We performed differential gene expression analysis among the 26 neuronal subtypes. The top DEGs of the EX_PALMD excitatory neuron include *CAMK2D* and *PDE1A*, which encode calcium/calmodulin-dependent protein kinase II delta and phosphodiesterase 1A proteins, respectively. The EX_HTR2C neuron expressed high levels of *HTR2C* (5-hydroxytryptamine receptor 2C protein), which is a G protein–coupled receptor (*GPCR*) that couples with Gq/G11 and mediates excitatory neural transmission [30]. This group of neurons also expressed high levels of *TRPM3* (transient receptor potential cation channel subfamily M member 3 protein), which may play a role in regulating the excitability of these neurons [31]. *CBLN4* is a member of a small secreted protein family that contains a C1Q domain, and members of this family participate in the regulation of neurexin signaling during synaptic development [32]. We defined 2 groups of excitatory neurons that showed high expression of the *CBLN4* gene, which we named EX_CBLN4-1 and EX_CBLN4-2. EX_CBLN4-1 was found to express top marker genes of *TLL1* and *NDST4*, which encode an astacin-like, zinc-dependent metalloprotease, and an N-deacetylase and N-sulfotransferase, respectively. EX_CBLN4-2, on the other hand, was identified as a type of cell that showed high expression of *NEFL, NEFM*, and *NEFH*, which encode the heavy, medium, and light chains that make up neurofilaments, respectively. High expression of the similar neurofilament-associated signals was also found in the mouse basal ganglia [14, 33, 34]. EX_ZMYM4 neurons expressed high levels of *ZMYM4*, a zinc finger protein, which plays a role in regulating cell morphology and cytoskeletal organization [35], while also expressing *FAM19A1*, a member of a conserved chemoattractant-like protein family abundant in the mouse and human central nervous system [36]. EX_APOC1B neurons expressed high levels of *APOC1B* and *APOE*, which encode apolipoprotein C-I and apolipoprotein E, respectively.

In addition to snRNA data, we mapped snRNA-seq neuronal cell types to snATAC-seq neuronal cell types by label transfer, as described above. In the snATAC-seq data, we identified 4 types of excitatory neuronal subtypes (EX_PALMD, EX_HTR2C, EX_CBLN4-1, and EX_ZMYM4) (Fig. 2C) and enriched with gene scores for marker genes of corresponding transcriptomic cell types (Fig. 2D). Our analysis of both snRNA-seq and snATAC-seq data revealed that almost all EX_PALMD, EX_HTR2C, and EX_ZMYM4 were present in the amygdala, while EX_CBLN4-1 and EX_CBLN4-2 were restricted to the substantia nigra. Additionally, we observed that 88.9% of the snRNA-seq EX_APOC1B neurons were found in the amygdala, while the remaining EX_APOC1B neurons were found in the striatal caudate nucleus.

We identified 2 distinct subpopulations of IN_SST neurons that exhibited different molecular features and epigenetic characteristics. The majority of IN_SST-1 cells were found in the amygdala, whereas IN_SST-2 neurons were primarily distributed in the striatum (Fig. 2A, C and Supplementary Fig. S2A). Two subtypes of SST neurons displayed consistent nuclear distribution patterns in both RNA-seq and ATAC-seq data. Moreover, the 2 types of IN_SST neurons exhibited heterogeneity in terms of gene expression and regulation in snRNA-seq and snATAC-seq data, respectively (Fig. 2E). IN_SST-1 expressed high levels of neurexophilin 1 (*NXPH1*), while *NPY* is the marker for IN_SST-2 [37]. Genes involved in regulating monoatomic ion transmembrane transport, such as *ASIC2, ADCYAP1R1, DPP10*, and *RASGRF2*, and genes related to glutamate receptor signaling pathways, such as *GRIA1, GRIA3, GRID1*, and *GRIK1*, are highly expressed in IN_SST-1, whereas the highly expressed genes in IN_SST-2, such as *NPY, LHX6*, and *RELN*, were related to the regulation of central nervous system neuron development and axon guidance (Supplementary Fig. S2B). To explore the different transcriptional regulation between IN_SST neuron subtypes, we used snATAC-seq data to calculate the activity of TF binding motifs in IN_SST-1 and IN_SST-2 using Chromvar. We found that motif activity and significantly increased targeted gene scores of *TFAP4, NHLH2, ASCL2, TCF21*, and *ZNF238* were present in IN_SST-1, while increased motif activity and targeted gene scores of *POU5F1, SNAI2, POU2F3, POU2F1*, and *NR1D1* were found in IN_SST-2 (Supplementary Fig. S2C). Next, we utilized the cRE–gene link and TF binding motif database to establish the TF-regulated gene network of IN_SST neurons. We identified cell type–specific DEGs in TF-targeted genes (Supplementary Fig. S2D). We found that for the TFs with enriched binding motifs in IN_SST-1, the majority of the DEGs linked to them are from IN_SST-1 (*P* < 0.001), while for the TFs with enriched binding motifs in IN_SST-2, the majority of the DEGs linked to them are from SST2 (*P* < 0.001). These findings suggested the regulatory role of these transcription factors on the differential gene expression and related functions of IN_SST subtypes.

IN_PVALB neurons, which exhibit pan-expression of the *PVALB* gene, can be further classified into 4 subtypes, designated as IN_PVALB1-4 in snRNA-seq and snATAC-seq. IN_PVALB-1 neurons were mainly distributed in the globus pallidus and substantia nigra, while IN_PVALB-2 were mainly found in the striatum (including the striatal putamen and the striatal caudate). Most of IN_PVALB-3 was distributed in the globus pallidus, followed by Pu. IN_PVALB-4 neurons were almost restricted to the amygdala. We then performed the differentially expressed gene analysis and the gene ontology enrichment analysis among PVALB subtypes (Supplementary Fig. S2E, G). We found that the enriched pathways in the DEGs of IN_PVALB-1, IN_PVALB-2, and IN_PVALB-4 largely overlapped, mainly including axon development, axonogenesis, and synapse organization. In addition to modulation of chemical synaptic transmission, IN_PVALB-3 was also involved in pathways such as ATP synthesis coupled electron transport, mitochondrial ATP synthesis coupled electron transport, and so on (Supplementary Fig. S2G). Next, we explored the differential chromatin accessibility of PVALB subtypes and noted their high cell type specificity (Supplementary Fig. S2F). In summary, PVALB subtypes exhibit significant differences in both the epigenome and transcriptome, suggesting that these subtypes may have distinct functions.

### Transcriptional and regulatory heterogeneity of MSN subtypes

GABAergic MSNs are a specific neuronal population located in the striatum of the basal ganglia, representing 95.0% of the neurons in this region [38]. MSNs can be divided into 2 main subtypes: DRD1-MSN and DRD2-MSN. DRD1-MSNs project directly to the interface nuclei between the basal ganglia and the rest of the brain, while DRD2-MSNs project to the intermediate basal ganglia nucleus, which is indirectly connected to the interface nuclei [39]. We found that macaque DRD1-MSN (46.4% of MSNs) selectively expressed *DRD1* and *TAC1* genes (Fig. 2B), which encode substance P, neurokinin A, neuropeptide K, and neuropeptide gamma and stimulate the output structure of the basal ganglia [38]. DRD2-MSN (53.6% of MSNs) specifically expressed the *DRD2* gene (Fig. 2B), encoding the D2 subtype of the dopamine receptor, which inhibits the output structure of MSNs [38]. Consistent with the transcriptional MSN subtypes found in macaque striatum, we defined DRD1_MSN_Matrix and DRD2_MSN_Matrix using *STXBP6*, as well as DRD1_MSN_Striosome and DRD2_MSN_Striosome using *KCNIP1*. Additionally, we defined the D1_D2_hybrid using *GRIK1*, which was expressed in both DRD1 and DRD2 in the snRNA-seq [4]. Similarly, in the matched MSN subtypes in snATAC-seq, we found that these marker genes had highly specific gene activity (Fig. 2F and Supplementary Fig. S3A). Genes with high-density cRE–gene–associated regions were referred to as domains of regulatory chromatin (DORCs), and these genes were enriched in some known super-enhancers, indicating their crucial regulatory significance in determining cell identity [40]. In light of this, we aimed to investigate which DEGs in the MSN subtype may have regulatory importance. We defined 36 DEGs with a large number (>10) of cREs as DORCs based on our established cRE–gene pairs (Fig. 2G). Consistent with previous studies, gene activity of most DORCs had the same cell type specificity as gene expression (Fig. 2H, I) [40]. Furthermore, we found that some DORCs, such as *CNR1, HOMER1, ADORA2A*, and *DRD2*, have been reported to be downregulated in patients with Huntington's disease in the striatum [41]. Our data showed that *CNR1* and *HOMER1* were highly expressed and active in DRD1-MSN, while *ADORA2A* and *DRD2* were highly expressed and active in DRD2-MSN. These results suggest that these DORCs, especially those downregulated in disease, have significant research implications. In addition to analysis of *cis*-regulatory elements in MSN subtypes, the regulatory function of TFs is of great importance in determining cell identity. Therefore, we systematically calculated the differential TF motif activity in MSN subtypes using snATAC-seq (Supplementary Fig. S3B). We found that the TF NPAS2 had high activity in the D1_D2_hybird (Fig. 2J). The core circadian protein NPAS2 has been reported to negatively regulate the nucleus accumbens, and disruption of NPAS2 produces augmented cocaine preference [42]. By establishing a TF regulatory network (Method), we found that genes regulated by NPAS2 were significantly more highly expressed in the D1_D2_hybrid MSN subtype in snRNA-seq compared to other MSN subtypes (Fig. 2J). These results further highlight the importance of NPAS2 in the striatum.

It is interesting to note that a similar group, known as eMSN, has been found in the mouse striatum, which shares similarities with the D1_D2_hybrid group [14]. When integrated snRNA-seq with mice MSN data, we found that our DRD1-MSN, DRD2-MSN, and D1_D2_hybrid MSNs all have high correlation with mouse MSN1, MSN2, and eMSN (Supplementary Fig. S3C). Compared with DRD1-MSN and DRD2-MSN, the D1_D2_hybrid subtype had the most differentially expressed genes (86 of 118 DEGs in mouse, 152 of 179 DEGs in macaque) (Supplementary Fig. S3D). The D1_D2_hybrid subtypes of the 2 species shared 22 common DEGs (*P* = 5.35 × 10^−25^ by hypergeometric test), including *PBX1, PBX3, TSHZ1*, and *OLFM3*, which were involved in sensory organ development (GO:0007423, *P* = 0.36 × 10^−4^) and visual system development (GO:0150063, *P* = 1.6 × 10^−3^). Macaque has 130 diverged DEGs, including genes involved in the neuroactive ligand–receptor interaction (e.g., *GRIK1, TACR1*, and *RXFP1*) and genes involved in cell morphology and neuron differentiation pathways (e.g., *APP, EDN1, EPHA4, EPHA5*, and *STMN1*), supporting the ideas that primate MSN neurons have higher complexity in signal transduction function (Supplementary Fig. S3E, F) [43].

### Molecular specialized astrocyte subtypes across basal ganglia

Astrocytes, located in the central nervous system (CNS), are responsible for a range of functions, including transmitting nerve signals, promoting synaptic genesis and transmission, and repairing damage to neurons [44–46]. Dysfunction of astrocytes is implicated in various neurodegenerative diseases [47–49]. Therefore, identifying and characterizing the subtypes of astrocytes can aid in a better understanding of their molecular basis for functions. Out of the 23 nonneuronal cell types in snRNA-seq, 5 astrocyte subtypes were identified (Fig. 3A, B and Supplementary Fig. S4A). We found nucleus-specific distribution of AST subtypes. For instance, AST4 was restricted to the substantia nigra, AST5 was mostly found in the amygdala, and AST2 only existed in the striatum, including the caudate nucleus and putamen (Fig. 3A and Supplementary Fig. S4C). Then, we performed the gene module analysis based on the top 5,000 variable features of AST and further correlated the 5 gene modules to the corresponding AST subtypes (Fig. 3C). Gene ontology analysis of enriched genes in each module revealed that module 1 (corresponding to AST2, Supplementary Fig. S4B) and module 5 (corresponding to AST1, Supplementary Fig. S4B) were related to known AST functional pathways, such as neural projection, transmembrane transport, synaptic transmission, and regulation of cell morphogenesis [50, 51].

Involvement of astrocytes in the immune response has been reported in previous studies [48, 49]. We found that the genes in module 4 (AST3), such as *CX3CR1, CSF1R*, and *IL18*, were related to inflammatory responses, supporting the idea that astrocytes were associated with inflammation. Similarly, we also found high expression of corresponding genes in AST subtypes in mice [31]. Astrocytes have been implicated in neurodegenerative disorders and may contribute to striatal neuron loss or dysfunction in Huntington's disease [50]. High expression of dynein axonemal heavy chain genes (including *DNAH3, DNAH5, DNAH7, DNAH6, DNAH9, DNAH11*) was found in module 2 (AST5) (Supplementary Fig. S4D). Mutations in these genes can cause striatal atrophy and impair axon growth of striatal neurons, possibly leading to Huntington's disease [52]. AST4 is a specific subtype of astrocytes only found in the substantia nigra. It expressed high levels of *ALDH1A1, DDC*, and *SLC6A3* genes, which are associated with the function of gene module 3 involved in dopaminergic neurogenesis (WP2855, *P* = 4.7 × 10^−11^) [53]. Additionally, AST4 expressed a high level of *MAP3K5, MAPK10*, and *TUBB3*, which are enriched in Alzheimer's disease–related pathways (hsa05010, *P* = 2.0 × 10^−07^) (Fig. 3D) [54, 55]. Consistently, we also identified a group of SN-specific epigenetic AST subtype AST3(A) in the snATAC-seq data (Supplementary Fig. S4C), which had a high correlation with transcriptomic AST4 cells in the snRNA-seq data (Fig. 3F). We found that the binding motif activities of transcription factors RFX4 and PBX3, specifically expressed in AST4, were significantly high in AST3(A) (Fig. 3E, G), suggesting that role of RFX4 and PBX3 in regulating specific transcriptomics of AST4 [56]. In addition, by overlaying the cRE–gene covariability map and TF binding motif data along the genome axis, we revealed a potential *cis*-regulatory relationship of pathogenic genes specific to AST4, such as *SLC39A12*, in neurodegenerative diseases such as Alzheimer's disease (Fig. 3H) [57].

### Transcriptional regulation on species-conserved and species-divergent genes in substantia nigra cells

**Figure 4:**
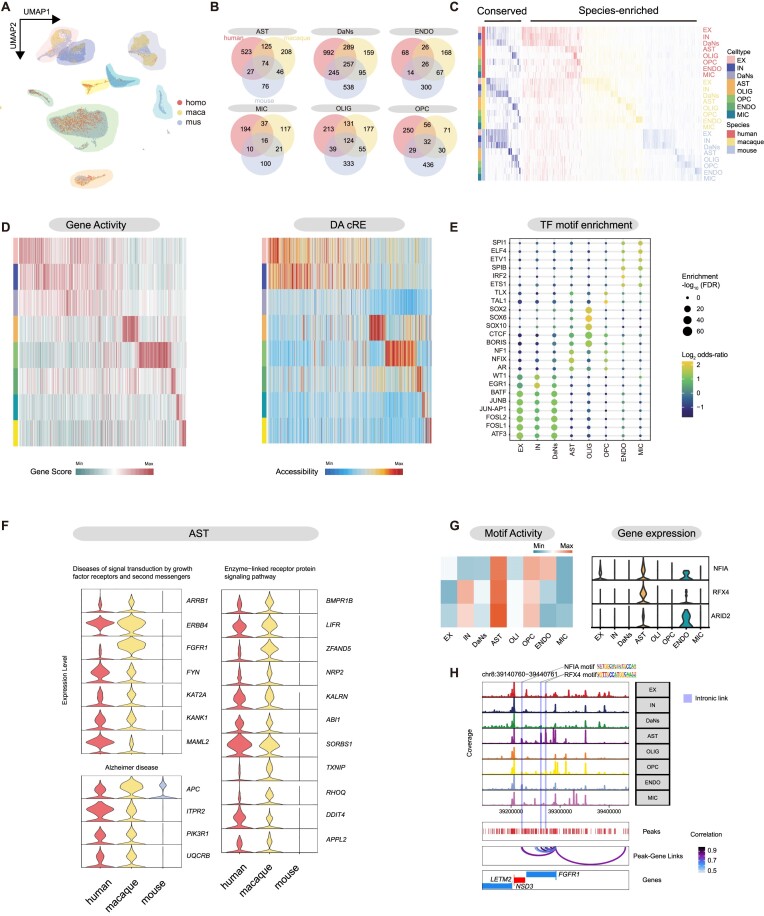
Cross-species comparison of cell type similarity and heterogeneity in the substantia nigra. (A) Coembedding of human, monkey, and mouse substantia nigra data, with colors representing different species. (B) Venn diagrams showing DEGs of SN cell types shared across species. (C) Heatmap illustrating the expression patterns of both cross-species conserved and species-specific DEGs. (D) Gene activity values of conserved DEGs across species (left panel) and their corresponding DA cRE activity values. (E) Transcription factors enriched in the DA cREs linked to conserved differentially expressed genes. (F) Violin plot representing AST-specific enrichment of differentially expressed genes in the SN related to functional pathways such as diseases of signal transduction by growth factor receptors and second messengers, Alzheimer's disease, and enzyme-linked receptor protein signaling pathway. (G) Heatmap showing AST-specific activity of TF motifs in SN (left) and violin plot displaying the specific expression of these TFs in AST (right). (H) Genome track visualization of the diseases of signal transduction by growth factor receptors and second messenger–related gene *FGFR1* locus. Inferred peak–gene links for distal or intronic regulatory elements containing transcription factor motif binding sites for NFIA and RFX4.

The SN is considered the primary input region of the basal ganglia, and its dysfunction is implicated in a set of neurological disorders, including Parkinson's disease, Huntington's disease, schizophrenia, and obsessive–compulsive disorder [58]. DaNs originating from the SN play a critical role in movement, cognition, emotion, and reward processes, and their dysfunction is a hallmark of neurodegenerative diseases [59]. To better understand the species-conserved and divergent transcriptomics in the SN cells between rodents and primates, we integrated single-cell datasets of excitatory neurons, inhibitory neurons, dopamine neurons, and nonneuronal cells from the SN of human, macaque, and mouse [10, 14] (Fig. 4A and Supplementary Fig. S5A, B). We demonstrated that the major cell types identified by SN region across species were conserved. For each species, we calculated DEGs in the major cell type and compared DEGs of the same cell type. Our analysis revealed that 16 to 257 cell type DEGs were conserved across species, and a greater number of cell type DEGs were shared between human and monkey (Fig. 4B, C). To further explore the regulatory mechanisms of these species-conserved DEGs, we used corresponding snATAC-seq data of macaque to depict the gene activity scores of species-conserved DEGs, as well as the linked DA cREs (Fig. 4D). To reveal potential TFs that may modulate these species-conservative genes, we identified binding motifs enriched in the cell type DA cREs linked to these conserved DEGs (Fig. 4E). For instance, motifs enriched in neurons included ATF3, FOSL1/2, JUNB, and BATF, while motifs enriched in astrocytes (ASTs) included NFIX and NF1, which are crucial for the maturation and growth of ASTs [60, 61]. Motifs enriched in OLIG were associated with the SOX10 function, which is necessary for the survival of oligodendrocytes that wrap axons to form myelin sheaths [62]. Finally, motifs enriched in endothelial cells (ENDO) and microglia (MIC) included ETS1, IRF2, SPIB/1, ELF4, and ETV1. In summary, we characterized these DEGs as cross-species conserved at the transcriptional level and identified the candidate transcription factor that may exert the transcriptional regulation on those genes [63].

DEGs shared by human and macaque but not found in mouse are considered primate-specific DEGs. In our analysis, we discovered that DaNs harbored the highest number of primate-specific DEGs (*n* = 289), followed by OLIG (*n* = 131), AST (*n* = 125), OPC (*n* = 56), MIC (*n* = 37), and ENDO (*n* = 26) (Fig. 4B). We then examined the pathways involved in the primate-specific DEGs, such as primate-specific DEGs in DaNs enriched in pathways involved in ion transmembrane transport, neurodegeneration, and Parkinson's disease (Supplementary Fig. S5C). Our prior research indicated that the SN-specific AST4 is involved in neurodegenerative diseases (Fig. 3D). We found that primate-specific DEGs were enriched in different pathways in the AST of the SN, including *APC, ITPR2*, and *PIK3R1*, which are related to Alzheimer's disease, as well as *ERBB4, FGFR1* and *FYN*, which are associated with diseases related to signal transduction by growth factor receptors and second messengers (Fig. 4F) [64, 65]. To gain insight into the regulatory mechanisms of primate-specific genes in the AST of the SN, we focused on key TFs involved in transcriptional regulation. We observed specific gene expression of transcription factors NFIA, RFX4, and ARID2 in the AST of the SN, which are associated with AST maturation [66], and found higher enrichment of their binding motifs in AST from the SN (Fig. 4G). By utilizing the potential regulatory relationships of the TF gene, an AST high-activity transcription factor regulatory network was constructed. Furthermore, the pathways related to the enrichment of primate-specific DEGs in AST cell types were identified and labeled within the regulatory network (Supplementary Fig. S5D). We observed that the *FGFR1* gene in the diseases of signal transduction by growth factor receptors and second-messenger pathway contained an NFIA and RFX4 motif binding site in its cRE. This revealed that the transcription factor NFIA and RFX4 may regulate the expression of the *FGFR1* gene through multiple motif binding sites (Fig. 4H).

### Cell type–specific *cis*-regulatory risk loci of human traits and diseases

Disease risk loci identified in genome-wide association studies (GWASs) show different degrees of enrichment in various cell type–specific regulatory elements. However, the lack of epigenetic data in previous basal ganglia datasets has resulted in a dearth of information on disease risk loci enriched in cell type–specific regulatory elements in the basal ganglia. To fill this gap, we mapped all coordinates of DA cREs from each subcluster to the orthologous coordinates in the human hg19 genome, then performed linkage–disequilibrium score regression (LDSC) analysis using GWAS summary statistics for human traits and diseases on the DA cREs [67] (Methods, Supplementary Table S4).

**Figure 5:**
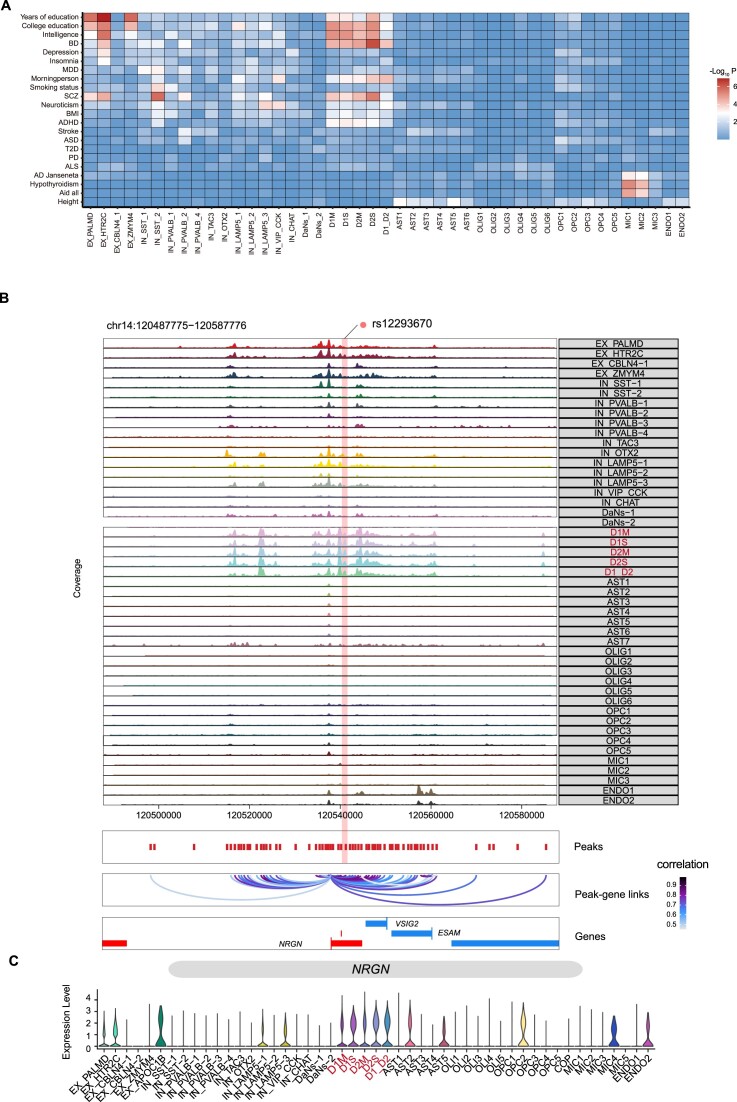
Cell type–specific regulatory landscape of GWAS loci in the basal ganglia. (A) Heatmap showing LDSC enrichment of GWAS traits and disorders in snATAC-seq clusters. BD, bipolar disorder; MDD, major depressive disorder; SCZ, schizophrenia; BMI, body mass index; ADHD, attention-deficit/hyperactivity disorder; ASD, autism spectrum disorder; T2D, type 2 diabetes; PD, Parkinson's disease; ALS, amyotrophic lateral sclerosis; AD, Alzheimer's disease. (B) rs12293670 GWAS locus and *cis*-regulatory architecture in snATAC-seq cell types. (C) Violin plot showing the expression levels of the *NRGN* gene across different subtypes in snRNA-seq.

We evaluated the enrichment of risk loci for neurological diseases and other human traits in basal ganglia cell types. We found that risk loci for human neurological diseases and related traits were mainly enriched in neuronal cells (Fig. 5A). IN_PVALB-2 neurons showed significant enrichment of sites for MDD (*P* = 3.1 × 10^−3^). PVALB neuron in the amygdala appeared to be particularly susceptible to the effects of chronic stress, which is considered the primary risk factor for MDD [68]. In addition, IN_CHAT neurons showed correlation with MDD (*P* = 3.2 × 10^−2^), in line with previous findings in mice that IN_CHAT regulates depression-like behavior [69]. We found significant enrichment of SCZ-associated sites in IN_SST-2 neurons (*P* = 1.8 × 10^−6^), and the reduction of SST neurons was found in patients with SCZ [70]. Notably, bipolar disorder (BD)–associated loci were significantly enriched in all subtypes of MSNs (*P* = 4.0 × 10^−5^, 4.1 × 10^−5^, 2.0 × 10^−5^, 5.1 × 10^−7^, and 8.6 10^−5^ for D1_MSN_Matrix, D1_MSN_Striosome, D2_MSN_Matrix, D2_MSN_Striosome, and D1_D2_hybrid, respectively). Transcriptome analysis in striatum tissue has connected the coexpressed gene module of BD patients and control to MSN neurons [71]. Our findings suggest that investigating neuronal subtypes is a promising avenue for studying neurological diseases.

Furthermore, we looked into the specific disease-associated loci that overlapped with cell type–enriched cREs. Significant enrichment of SCZ-associated sites was observed in D1_MSN_Matrix, D1_MSN_Striosome, D2_MSN_Matrix, D2_MSN_Striosome, and D1_D2_hybrid in MSN neurons (*P* = 1.1 × 10^−4^, 2.8 × 10^−4^, 5.8 × 10^−5^, 4.0 × 10^−6^, and 2.6 × 10^−3^ for D1_MSN_Matrix, D1_MSN_Striosome, D2_MSN_Matrix, D2_MSN_Striosome, and D1_D2_hybrid, respectively), in line with previous findings that genomic variants associated with SCZ map to MSN [9]. Subsequently, we found the enrichment of cREs in MSN subtypes that located in the SCZ-associated variant locus rs12293670 (Fig. 5B). SCZ risk variant rs12293670 has been attributed to the candidate gene *NRGN*, which exhibits specific expression in the human brain [72]. By overlaying peak–gene covariable maps with chromatin accessibility signal, we found the gene *NRGN* showed strong correlation with rs12293670 in MSN subtypes; moreover, we found the enriched gene expression of *NRGN* in transcriptomic MSN subtypes (Fig. 5C). Our results proposed medium spiny neurons as potential pathological and therapeutic targets for future SCZ studies.

By integrating human GWAS data and macaque cell type transcriptomic and chromatin accessibility data, we further attribute the risk loci and related genes to the specific cell type and provide the potential pathological and therapeutic candidates.

## Discussion

In this study, we utilized snRNA-seq and snATAC-seq techniques to profile the striatum, substantia nigra, globus pallidus, and amygdala in the macaque basal ganglia. While several studies have generated diverse datasets of the basal ganglia at the single-cell transcriptomic level [4, 10, 73], molecular architecture in basal ganglia has not been extensively explored at the level of sample diversity, multiomics, and transcriptional regulation of specialized molecular cell types in the complex basal ganglia region. Nonhuman primates are highly evolutionarily related to humans and possess comparable brain structures, which make them valuable animal models for studying human diseases [74]. In order to elucidate the pathogenesis of various diseases and prioritize potential therapeutic strategies, it is crucial to understand the cellular composition and molecular regulation of the macaque basal ganglia at both the transcriptomic and epigenomic levels. Our research represents the first comprehensive exploration and comparison of multiple basal ganglia regions in the macaque at the single-cell level of transcriptomics and epigenomics. In conclusion, our study provides a comprehensive data resource for nonhuman primate disease models related to basal ganglia areas, as it demonstrates the diverse and regional patterned regulatory network in basal ganglia cells.

The high-throughput single-cell sequencing technology provides an unprecedented opportunity for investigating the cellular composition and molecular features of the basal ganglia. By analyzing over 101,431 snRNA-seq and 170,608 snATAC-seq data from the macaque basal ganglia, we characterized the transcriptional and epigenetic features of neuronal cell types, including EX, IN, MSN, and DaNS, as well as nonneuronal cell types such as AST, OLIG, OPC, MIC, and ENDO. By further classifying major cell types based on different marker genes and accessible chromatin landscapes, we identified 49 subpopulations in snRNA-seq data and 47 subpopulations in snATAC-seq data. By converting chromatin accessibility data to gene activity data, we matched the neuronal subtypes from snATAC-seq to their corresponding counterparts in snRNA-seq neuronal subtypes and validated the consistency of cell identity through marker gene expression and activity. In this way, we were able to conduct in-depth analysis of the molecular basis of differential gene expression between these neuronal subtypes.

The basal ganglia primarily coordinate complex motor behaviors in the body by suppressing signal output. To gain a deeper understanding of the composition and regulatory mechanisms of inhibitory neurons in the basal ganglia, we further conducted a detailed comparative study at the molecular level to investigate the differences between subtypes of inhibitory neurons. We discovered SST heterogeneity in the amygdala-specific neuronal subtype and identified potential regulatory mechanisms that may account for differences in gene expression. We also identified PVALB subtypes that were region specific and demonstrated differences in transcriptional and epigenetic regulation, as well as significant functional differences between subtypes within the same neuron. Comparing the macaque and mouse MSNs [31], we discovered that our D1_D2_hybrid cells were the same population of cells as eMSN in mice, and eMSN was an “eccentric” MSN that significantly differed from the classic MSN in mice. We found that even within the same eMSN cell type, there were significant differences between species. Given the importance of MSNs and the specificity of eMSNs, this group should be included in further research.

Astrocytes are abundant glial cells in the central nervous system that provide nutrition and support for neurons. The study of astrocyte subtypes is crucial for understanding astrocyte-associated diseases due to their functional heterogeneity between and within brain regions [75]. Recently, the distinct subtypes of AST in the cortex and subcortical regions of mice have been revealed [76]. We classified our ASTs into 5 and 7 subtypes in snRNA-seq and snATAC-seq data, respectively. Gene module analysis revealed that a group of SN-specific transcriptomic AST4 is mainly associated with neurodegenerative diseases [77]. We also identified a cluster, AST3in our snATAC-seq data, that corresponds to AST4 in snRNA-seq data. Through integrated analysis of transcriptomics and epigenomics, we have gained a deeper understanding of the regulatory mechanisms underlying the SN-specific gene expression of this group of AST subtypes, providing a foundation for future disease research.

Based on the importance of SN in Parkinson's disease [78], we compared SN cell types among 3 species (humans, macaques, and mice) to gain insight into the fundamental molecular regulatory mechanisms diverged through evolution [79]. We also screened for DEGs specific to primates, which is essential for understanding the susceptibility to primate-specific and rare diseases [80]. We found that the DEGs conserved across species are mostly classical marker genes for cell types. Moreover, the DEGs conserved across species also exhibit significant differences in chromosomal activity between cell types. This suggests that in the future, the regulatory mechanisms of conserved DEGs across species can be studied through differences in transcription factor activity and chromatin accessibility between cell types [81]. Previous studies have reported the association of CNTN2 with OLIG differentiation [82]. Similarly, our prediction indicates that SOX10 regulates the conserved OLIG DEG CNTN2 gene, suggesting potential similarities in OLIG differentiation across different species.

We systematically analyzed primate-specific DEGs in AST cell types and found that highly active transcription factors, such as NFIA, RFX4, and ARID2, may regulate primate-specific DEGs enriched in different pathways (Supplementary Fig. S4D). Our proposal suggests that NFIA and RFX4 regulate PPFIA1 by controlling the cREs of the PPFIA1 gene. Additionally, based on the importance of DaNs, we performed Gene Ontology (GO) functional enrichment analysis of primate-specific DEGs in DaNs and obtained a series of genes associated with neurodegenerative diseases. This may provide further research directions for studying neurodegenerative diseases using nonhuman primate models.

Moreover, by combining epigenomic data with GWAS loci, disease risk can be linked to specific cell types. We found significant differences in the enrichment of different neuronal subtypes for disease, suggesting targeted investigation of specific cell types for different diseases. Additionally, by integrating our epigenomic and transcriptomic data, we identified a potential *cis*-regulatory relationship between *NRGN*, specifically expressed in MSNs, and SCZ GWAS loci. Linking diseases to relevant cell types and predicting regulatory sites for disease-associated genes can provide potential targets for future disease treatments.

Although we provided relatively comprehensive single-cell transcriptomic and epigenetic data of basal ganglia, there were still some limitations in our research. First, only 2 female monkeys were included in the analysis, so our analysis could not incorporate gender differences and might have overlooked basal ganglia–specific information in adult male macaques. Nevertheless, there was a good match between cell types in different regions of the 2 monkeys. Second, the high resolution of singleome snATAC-seq and snRNA-seq enables the generation of high-depth and high-throughput single-cell results. However, compared to multiome approaches such as snRNA-seq and snATAC-seq to study the same cells, there may be inherent sequencing technology biases and filtering criteria that result in differences in the number of cells obtained from singleome snRNA-seq and snATAC-seq in the same sample. Additionally, while algorithms can be used to match cell identities between singleome snRNA-seq and snATAC-seq, there may be limitations in these algorithms, potentially missing some cells in snATAC-seq that have less matching with snRNA-seq. However, the large number of cells with matching identities between snATAC-seq and snRNA-seq helps alleviate this issue. Furthermore, previous studies have shown a high overlap between the cRE–gene pairs established by singleome and multiome approaches [83]. This indicates that our transcriptional regulatory research is reliable. In the future, we anticipate that the use of high-depth multiome technologies will allow for the generation of more comprehensive transcriptional regulatory landscapes in the basal ganglia region.

In summary, our macaque basal ganglia cell atlas provides valuable insights into the comprehensive transcriptome and epigenome of the diverse and functional related cell populations in the macaque basal ganglia. This information will serve as a foundational resource for future preclinical investigations on neurological disorders related to basal ganglia.

## Methods

### Ethics statement

The study was carried out in accordance with regulations on animal research and was approved by the institutional review boards on the ethics committee of BGI (permit BGI-IRB A21025-T1).

### Sample preparation and single-cell nucleus isolation

Tissue samples were collected from the basal ganglia of two 72-month-old female crab-eating macaques (*M. fasicularis*) and immediately frozen in liquid nitrogen. The samples from both monkeys included the Cd, Pu, SN, and GP, while only 1 monkey (MK2) had a sample from the AMY. The collected samples were subsequently processed for single-nucleus RNA-seq and snATAC-seq analysis.

As previously described [1], the method for single-nucleus preparation involved placing the frozen monkey brain tissue block into 1 mL of prechilled Dounce homogenization buffer and homogenizing it using 10 loose strokes and 10 tight strokes with the Dounce homogenizer, which was immersed in ice. Then, 2 mL of homogenization buffer was added to the Dounce homogenizer, and the homogenate was filtered through a 40-μm cell strainer (Miltenyi Biotech) into a 15-mL conical tube. Finally, the sample was centrifuged at 900 *g* for 10 minutes to pellet the cell nuclei. The overall quality and quantity of the isolated nuclei were estimated using fluorescence microscopy.

### Library preparation and sequencing for scRNA-seq and snATAC-seq

To prepare the snRNA-seq library for the DNBelab C4 RNA-seq and C4 scATAC-seq analysis based on droplet technology, the DNBelab C series single-cell library preparation kit (MGI, #1000021082) and DNBelab C Series Single-Cell ATAC Library Prep Set (MGI, #1000021878) were employed.

As previously described [2], single-cell nuclear suspension was prepared from obtained mononuclear RNA samples, followed by 2 washes with phosphate-buffered saline (containing 0.04% bovine serum albumin). The nuclear suspension was then resuspended and filtered through a 40-μm cell strainer, and the cell suspension concentration was measured and recorded, followed by measurement and recording of the nuclear concentration. DNBelab C series single-cell library preparation kit (MGI, #1000021082) was used to prepare the nuclear suspension into droplets, which completed cell lysis and mRNA capture by magnetic beads in the droplets. Next, the single-cell magnetic beads were recovered using a lysis reagent recovery system (vacuum pump required), and the magnetic bead–captured mRNA was transcribed into cDNA. The cDNA was then subjected to double-stranded synthesis, followed by amplification and screening of the obtained cDNA and Oligo products. Subsequently, polymerase chain reaction (PCR) was used to barcode the Oligo products for subsequent preparation into Oligo on-machine libraries. Finally, the cDNA product was fragmented, end-repaired, connected, PCR-amplified, denatured, circularized, and digested to prepare a single-stranded DNA library. After library preparation, the library was sequenced using the DIPSEQ T1 sequencing platform of the China National GeneBank (Shenzhen).

To perform snATAC-seq, as previously described [4], preextracted nuclei were subjected to a transposition reaction, followed by droplet generation using a syringe according to the protocol of the DNBelab C Series Single-Cell ATAC Library Prep Set (MGI, #1000021878). Next, demulsification, enzyme treatment, and PCR amplification reactions were conducted, and finally, a circular library was constructed and subjected to sequencing.

### Processing and quality control of RNA-seq data

We performed filtering and demultiplexing of DNBelab C4 RNA-seq raw sequencing reads using PISA (RRID:SCR_015749) (version 0.7). Next, we employed STAR (RRID:SCR_004463) (version 2.6.1a) to demultiplex these reads. For alignment, we used a modified GTF file of the Macaca_fascicularis_5.0 genome, which contained both introns and exons. The aligned sequences were then sorted using sambamba (RRID:SCR_024328) (version 0.7). Finally, we obtained the UMI counts matrix of the cell to the gene. We removed cells that had unannotated genes, fewer than 3 detected genes, or mitochondrial expression exceeding 5%. Additionally, cells with fewer than 500 genes and those with the top 5% of the highest number of genes were filtered out.

To analyze the resulting matrix, we utilized the Seurat package (RRID:SCR_016341) (V 4.0.3) [5] for dimensionality reduction and clustering. We used monkeys as a batch and applied NormalizeData and FindVariableFeatures processes to 2 monkeys separately. We then selected 2,000 feature genes using the SelectIntegrationFeatures equation, identified anchor points using the FindIntegrationAnchors equation, and integrated the data using the IntegrateData equation. To reduce dimensionality and cluster, we employed ScaleData, RunPCA, RunUMAP, FindNeighbors, and FindClusters equations. For identifying differentially expressed genes, we used either FindAllMarkers or FindMarkers equations.

After obtaining the DEGs, we compared the GO enrichment between different clusters using the compareCluster equation. Alternatively, we used the Metascape (RRID:SCR_016620) to calculate the GO enrichment.

### Feature selection and module recognition in AST cell types using hotspot analysis

To identify the gene signature modules of AST, we used the hotspot package (V 0.9.0) of Python (3.8.12) [3] to perform Feature Selection and Module Recognition via Hotspot analysis on AST cell types. First, we identified the top 5,000 variable features in AST and removed the mitochondrial genes. Next, we applied the hotspot.Hotspot equation to create a hotspot object and set the neighborhood size to 30 by using the create_knn_graph function. The autocorrelations of each gene were then computed using the compute_autocorrelations function to identify the genes with the most informative variation. Next, we calculated the pairwise local correlation of features through hs_results.loc and compute_local_correlations functions. Finally, we generated the gene module clustering results by using the create_modules function.

### snATAC-seq data processing and quality control

We referred to previous literature and utilized the open-source PISA software workflow to process the snATAC-seq data of DNBelab C4 [84, 85]. We aligned the retained reads to the *M. fascicularis* genome and filtered out reads that aligned to mitochondrial or genomic scaffolds (including those starting with chrAQ, chrU, chrK, and those containing random segments), as well as reads with alignment quality less than 10 and PCR duplicates. The fragments obtained from each library in the aforementioned steps were used for downstream analysis.

### snATAC-seq data clustering and analysis

First, we performed initial clustering of the raw data using the ArchR package in R software (version 1.0.2) [17], retaining cells with more than 1,000 fragments and transcription start site (TSS) enrichment higher than 6. We calculated the doublet score for each cell using the addDoubletScores function, with the parameter filterRatio = 2 used to filter out cells that may be doublets. Before the initial clustering, we created a tiled matrix of 500-bp bins using the genome. Then, we used latent semantic indexing (LSI) to reduce the top 25,000 features of the tiled matrix to 30 dimensions, with 2 iterations. To ensure the accuracy of each iteration, we inputted all cells. Subsequently, batch correction was performed using the Harmony function with donors and brain regions, followed by identification of clusters using Seurat's shared nearest neighbour (SNN) graph clustering method at the default resolution of 0.8. Identified clusters were then used to call peaks with macs2, and different cell types' DA peaks were calculated using the getMarkerFeatures function with parameters “FDR ⇐ 0.01 & Log2 FC ≥ 2.” For the reclustering of the neuronal system and nonneuronal cells, we used the peak matrix generated by ArchR as input to Signac (version 1.1.0) for data normalization, feature selection, and dimensionality reduction analysis using the default workflow [86]. The data were then integrated by sample source and batch corrected using the RunHarmony function in the R package Harmony (version 0.1.0) [87]. Finally, a 2-dimensional clustering result was obtained through UMAP analysis using 30 dimensions.

### Co-clustering of snRNA-seq and snATAC-seq data

First, we extracted the cell gene score and peak matrix from the ArchR object of scATAC-seq. Then, we performed normalization, feature selection, and dimensionality reduction analysis on the scATAC-seq data using the Signac standard pipeline in R. For scRNA-seq data, we performed corresponding dimensionality reduction analysis using the standard pipeline in Seurat. Subsequently, we used the FindTransferAnchors function to calculate anchors between different omics cells using the gene score matrix from scATAC-seq and the gene expression matrix from scRNA-seq, with the top 2,000 VariableFeatures (calculated from scRNA-seq data) in common. To improve the accuracy of anchors, we set k.anchor to 20. Subsequently, these anchors were used to assign a predicted ID to each cell in scATAC-seq, and scATAC cells with a score greater than 0.6 were retained and given a predicted gene expression matrix. The data from the 2 datasets were then coembedded into a low-dimensional space with 30 dimensions using standard UMAP (RRID:SCR_018217) analysis.

### Linking gene expression from snRNA-seq to *cis*-regulatory elements from snATAC-seq

To improve the accuracy of the peak-to-gene linkages, we performed supervised integration by inputting scRNA-seq data by cell type into the ArchR object of scATAC-seq. Subsequently, we used the addPeak2GeneLinks function in ArchR to establish links between genes and peaks and then retained the links with a correlation greater than 0.45 and an FDR less than 0.01.

### TF binding motif activity calculation

We calculated the enrichment of TF binding motifs by inputting peak activity matrices containing different cell populations into the R package chromVAR (version 1.18.0) [88]. First, we calculated the GC bias using the BSgenome.Mfascicularis.NCBI.5.0 genome, and then we downloaded the human TF binding motif database (human_pwms_v2) from the chromVARmotifs R package. We used the matchMotifs function to select peaks that retained TF binding motifs. Next, we calculated the activity of TF binding motifs for each cell using the computeDeviations function and extracted the bias-corrected deviations matrix for downstream analysis. We retained TFs with a variability greater than 1.5 by computing the variability for each TF. We then used the cell type–specific TF binding motif enrichment matrix to perform Wilcoxon tests to detect TFs that were enriched in different cell types.

### Constructing a TF regulatory network

If a cRE or DA cRE associated with a gene contains a binding motif for a certain TF, it is defined that this TF may regulate the gene. If the cRE or DA cRE falls within the promoter, intron, exon, or distal region of the gene, different modes of regulation are named accordingly. Finally, by inputting the TF–gene associations of different cell types into Cytoscape, a regulatory network is constructed. The color of the edges indicates the different modes of regulation of the transcription factors, while the color of the nodes represents the transcription factors and differentially expressed genes in different cell types.

### Predicting the transcription factor enrichment of DA cRE

Input the DA cRE sets of different cell types into Homer (version 4.11) to calculate the activity of TF binding motifs [89]. Retain the results in the knownResults output file and use the Benjamini–Hochberg test to assess the enrichment levels of different TFs.

### Evaluating GWAS enrichment using cell type–specific open regions

We used LDSC to analyze the genetic variation in differentially accessible regions of different cell types and its correlation with GWAS results. First, we retained DA cRE with FDR ⇐ 0.1 and Log2 FC ≥ 0.5, and filtered out cell types with fewer than 100 DA cRE. We then used the liftover software to convert genome data to human hg19 genome data. To prepare for cluster-specific peak analysis for LDSC, we used the make_annotation.py script and then calculated linkage disequilibrium (LD) scores of single-nucleotide polymorphisms in differentially accessible peaks using the ldsc.py script with 1000 Genomes phase 3 data. Then, we downloaded GWAS summary statistics data from the UK Biobank database and publications. Finally, we input HapMap3 single-nucleotide polymorphisms and corresponding 1000G_EUR_Phase3_baseline data and used the standard process to calculate cell type–specific genetic variation.

## Supplementary Material

giad095_GIGA-D-23-00121_Original_Submission

giad095_GIGA-D-23-00121_Revision_1

giad095_Response_to_Reviewer_Comments_Original_Submission

giad095_Reviewer_1_Report_Original_Submission Trygve Bakken -- 6/16/2023 Reviewed

giad095_Reviewer_1_Report_Revision_1 Trygve Bakken -- 8/16/2023 Reviewed

giad095_Reviewer_2_Report_Original_Submission Yiqiao Wang -- 7/5/2023 Reviewed

giad095_Reviewer_3_Report_Original_Submission Dijun Chen -- 7/7/2023 Reviewed

giad095_Supplemental_Files

## Data Availability

The raw FASTQ data for both snRNA-seq and snATAC-seq generated in this study are available in the EBI ENA under bioproject PRJNA970949 and the CNGB Nucleotide Sequence Archive using the accession code CNP0003589. All additional supporting data are available in the *GigaScience* database, GigaDB [90].

## Additional Files

**Supplementary Fig. S1**. Quality assessment of snRNA-seq and snATAC-seq data. (A) Bar graph representing the proportion of subtypes in snRNA-seq, with colors consistent with Fig. 1A. (B) Box plot of plot of unique molecular identifiers counts (UMI) (up), detected gene number (middle), and proportion of mt (mitochondrial) genes of snRNA-seq cells in each snRNA-seq cell type. (C) Bar graph representing the proportion of subtypes in snATAC-seq, with colors consistent with Fig. 1B. (D) Box plot showing the TSS enrichment, which is calculated as the average abundance of read counts in the 50 bp upstream and downstream of the TSS, divided by the average accessibility of the TSS flanking positions (±1,900–2,000 bp) (top). The TSS ± 2 kb ratio, which represents the proportion of peaks located within 2 kb from the gene TSS site, is shown in the middle panel. The fragment counts (bottom) of snATAC-seq cells in each snATAC-seq cluster are also displayed. (E) UMAP plot showing the comparison of snRNA-seq before and after batch correction (top), with the color indicating the donor source. The snATAC-seq plot (bottom) also shows the comparison before and after batch correction with the donor source consistent with snRNA-seq. The amygdala region is only present in monkey2 in both datasets, while monkey1 is the exclusive possessor of the SN region in snATAC-seq. (F) UMAP plot displaying the region of origin for snRNA-seq (top) and snATAC-seq (bottom), with colors indicating different regions. (G) Heatmaps showing differential open chromatin activity of 429,541 snATAC-seq DA cREs identified by bias-matched differential testing (FDR < 0.01 and Log2 FC ≥ 2) across 47 cell types.

**Supplementary Fig. S2**. Heterogeneity analysis of neuronal subtypes. (A) UMAP plot displays the subclassification of basal ganglia neurons from snRNA-seq (left) and snATAC-seq (right), with colors represent different region of cells. (B) Gene Ontology terms enriched among genes with distinct expression pattern between IN_SST-1 and IN_SST-2. (C) Violin plots representing the motif enrichment and targeted gene scores of selected TFs that were significantly upregulated in IN_SST-1 and IN_SST-2. (D) TF regulatory networks showing the predicted target DEGs for transcription factors TFAP4, NHLH2, ASCL2, TCF21, and ZNF238 in IN_SST-1 subtype inhibitory neurons and the predicted target DEGs for transcription factors NR1D1, POU2F3, POU2F1, POU5F1, and SNAI2 in IN_SST-2 subtype inhibitory neurons. (E) Heatmap showing the top 10 most enriched genes in each PVALB type. (F) Heatmap of subtype-specific activity patterns of DA cREs in IN_PVALB neurons from snATAC-seq data, marked with DEGs linked to corresponding snRNA-seq cell types. (G) Dot plot showing enriched pathways for the IN_PVALB subtype DEGs.

**Supplementary Fig. S3**. Comparative analysis of MSN neurons across species. (A) Top: MSN reclustering UMAP plot of snRNA-seq (left) and snATAC-seq (right). Middle: KCNIP1 gene expression (left) and gene activity (right). Bottom: STXBP6 gene expression (left) and gene activity (right). (B) TF motif scores for each MSN subtype cluster. (C) Heatmap displaying the correlation between macaque and mouse MSNs, with macaque DRD2-MSN4 showing the strongest correlation with mouse eMSNs. (D) Bar graph showing the number of DEGs in macaque and mouse MSNs, with macaque D1_MSN including D1-MSN_Matrix and D1-MSN_Striosome, while D2_MSN comprises D2-MSN_Matrix and D2-MSN_Striosome. Mouse eMSN refers to D1_D2_hybrid. (E) Volcano plot displaying the DEGs in macaque and mouse D1_D2_hybrid. (F) Gene Ontology terms enriched among DEGs specific to macaque and mouse D1_D2_hybrid.

**Supplementary Fig. S4**. Heterogeneity of nonneuronal cells. (A) DEGs (left) and regional distribution (right) of nonneuronal cell subtypes including OPC, MIC, OLIG, and ENDO. (B) Visualization of 5 distinct gene modules in AST subtype on UMAP (left) and highlights of AST subtype. (C) Histogram depicting regional distribution of AST subtypes in snRNA-seq (left) and snATAC-seq (right). (D) Specific expression of Huntington's disease–related genes in the AST5 cluster.

**Supplementary Fig. S5**. Cross-species conservation and primate specificity of SN cell types. (A) UMAP plot from Fig. 4A, grouped by species and colored by within-species clusters. (B) Proportion of nuclei that overlap between macaque (rows, color by Fig. 4C) and human or mouse clusters in the integrated space. (C) Violin plot showing enrichment of DEGs specific to DaN neurons in the primate brain for functional pathways related to brain development, actin filament–based processes, enzyme-linked receptor protein signaling pathway, and cardiac muscle contraction. (D) Transcription factor regulatory network diagram depicting NFIA, ARID2, and RFX4; highly active transcription factors in SN AST cell types; and their predicted target DEGs. The colors represent DEG enrichment in different pathways.

**Supplementary Table S1**. Single-nucleus RNA-seq sample annotation and quality information,

**Supplementary Table S2**. Single-nucleus ATAC-seq sample annotation and quality information.

## Abbreviations

AMY: amygdala; ASD: autism spectrum disorder; AST: astrocyte; Cd: caudate; cREs: *cis*-regulatory elements; DA cREs: differential accessible cRE; DEG: differentially expressed gene; ENDO: endothelial cell; EX: excitatory neuron; GP: globus pallidum; GO: Gene Ontology; GWAS: genome-wide association studies; IN: inhibitory neuron; LDSC: linkage-disequilibrium score regression; MDD: major depressive disorder; MIC: microglia; MSN: medium spiny neurons; OPC: oligodendrocyte progenitor cell; OLIG: oligodendrocyte; Pu: putamen; SCZ: schizophrenia; SN: substant.
