# Supplementary material for: Deciphering the distinct transcriptomic and gene regulatory map in adult macaque basal ganglia cells
Source: Gigascience. 2023 Dec 13;12:giad095. doi: 10.1093/gigascience/giad095 (PMC10716911; doi:10.1093/gigascience/giad095)
Supplement: giad095_GIGA-D-23-00121_Revision_1 [file giad095_giga-d-23-00121_revision_1.pdf]

GigaScience

Deciphering the distinct transcriptomic and gene regulatory map in adult macaque basal ganglia cells

--Manuscript Draft--

**Manuscript Number:**	GIGA-D-23-00121R1
**Full Title:**	Deciphering the distinct transcriptomic and gene regulatory map in adult macaque basal ganglia cells
**Article Type:**	Data Note
**Funding Information:**	National Key Research and Development Program (No.2022YEF0203200) — Not applicable
**Abstract:**	Background<br><br>The basal ganglia are a complex of interconnected subcortical structures located beneath the mammalian cerebral cortex. The degeneration of dopaminergic neurons in the basal ganglia is the primary pathological feature of Parkinson's disease. Due to a lack of integrated analysis of multi-omics datasets across multiple basal ganglia brain regions, very little is known about the regulatory mechanisms of this area.<br><br>Findings<br><br>We utilized high-throughput transcriptomic and epigenomic analysis to profile over 270,000 single-nucleus cells to create a cellular atlas of the basal ganglia, characterizing the cellular composition of four regions of basal ganglia in adult macaque brain, including the striatum (STR), substantia nigra (SN), globus pallidum (GP), and amygdala (AMY). We found a distinct epigenetic regulation on gene expression of neuronal and non-neuronal cells across regions in basal ganglia. We identified a cluster of SN-specific astrocyte associated with neurodegenerative diseases, and further explored the conserved and primate-specific transcriptomics in SN cell types across human, macaque and mouse. Finally, we integrated our epigenetic landscape of basal ganglia cells with human disease heritability, and identified a regulatory module consisting of candidate cis-regulatory elements that are specific to medium spiny neurons and associated with schizophrenia (SCZ).<br><br>Conclusions<br><br>In general, our macaque basal ganglia atlas provides valuable insights into the comprehensive transcriptome and epigenome of the most important and populous cell populations in the macaque basal ganglia. We have defined over 50 cell types with transcriptomic and epigenomic profiles, some of which exhibit region-specificity, and characterized the molecular relationships underlying these brain regions.
**Corresponding Author:**	Ying Lei<br>BGI-Shenzhen: BGI Group<br>shenzhen, CHINA
**Corresponding Author Secondary Information:**	
**Corresponding Author's Institution:**	BGI-Shenzhen: BGI Group
**Corresponding Author's Secondary Institution:**	
**First Author:**	Yunong Sun
**First Author Secondary Information:**	
**Order of Authors:**	Yunong Sun<br>Zihao Li<br>Lingjun Ding

Powered by Editorial Manager® and ProduXion Manager® from Aries Systems Corporation

	Jing Yang
	Jinrong Huang
	Mengnan Cheng
	Liang Wu
	Zhenkun Zhuang
	Cheng Chen
	Yunqi Huang
	Zhiyong Zhu
	Siyuan Jiang
	Fubaoqian Huang
	Chunqing Wang
	Longqi Liu
	Shiping Liu
	Ying Lei
**Order of Authors Secondary Information:**	
**Response to Reviewers:**	We have compiled our responses to the reviewer and editor comments in the attached Point-by-point response document.
**Additional Information:**	
**Question**	**Response**
Are you submitting this manuscript to a special series or article collection?	No
**Experimental design and statistics**<br><br>Full details of the experimental design and statistical methods used should be given in the Methods section, as detailed in our Minimum Standards Reporting Checklist. Information essential to interpreting the data presented should be made available in the figure legends.<br><br>Have you included all the information requested in your manuscript?	Yes
**Resources**<br><br>A description of all resources used, including antibodies, cell lines, animals and software tools, with enough information to allow them to be uniquely identified, should be included in the Methods section. Authors are strongly	Yes

encouraged to cite Research Resource Identifiers (RRIDs) for antibodies, model organisms and tools, where possible.<br><br>Have you included the information requested as detailed in our Minimum Standards Reporting Checklist?	
**Availability of data and materials**<br><br>All datasets and code on which the conclusions of the paper rely must be either included in your submission or deposited in publicly available repositories (where available and ethically appropriate), referencing such data using a unique identifier in the references and in the "Availability of Data and Materials" section of your manuscript.<br><br>Have you have met the above requirement as detailed in our Minimum Standards Reporting Checklist?	Yes

Manuscript

Click here to access/download;Manuscript;Manuscript-Oct 10_final.docx

Deciphering the distinct transcriptomic and gene regulatory map in adult macaque basal ganglia cells

Zihao Li[1,2,5], Yunong Sun[1,2,5], Lingjun Ding[2], Jing Yang[2], Jinrong Huang[3], Mengnan Cheng[2], Liang Wu[3], Zhenkun Zhuang[2], Cheng Chen[1,2], Yunqi Huang[1,2], Zhiyong Zhu[1,2], Siyuan Jiang[1,2], Fubaoqian Huang[2,4], Chunqing Wang[1,3], Shiping Liu[2,3], Longqi Liu[1,2,3]✉, Ying Lei[3,]✉.

Affiliations

[1] College of Life Sciences, University of Chinese Academy of Sciences, Beijing 100049, China

[2] BGI Research, Hangzhou 310030, China

[3] BGI Research, Shenzhen 518083, China

[4] School of Biology and Biological Engineering, South China University of Technology, Guangzhou 510006, China

[5] These authors contributed equally.

✉Corresponding authors: leiying1@genomics.cn (Y.L.), liulongqi@genomics.cn (L.L.)

Zihao Li [0009-0003-3811-8274];

Yunong Sun [0009-0000-1538-8376];

Lingjun Ding [0000-0002-9183-7062];

Jing Yang [0000-0002-7973-5717];

Jinrong Huang [0000-0001-8085-9939];

Liang Wu [0000-0002-6784-0181];

Zhenkun Zhuang [0009-0001-5258-9203];

Longqi Liu [0000-0002-5828-5542];

Shiping Liu [0000-0003-2158-8424];
Ying Lei [0000-0002-4349-3074].

Abstract

Background

The basal ganglia are a complex of interconnected subcortical structures located beneath the mammalian cerebral cortex. The degeneration of dopaminergic neurons in the basal ganglia is the primary pathological feature of Parkinson's disease. Due to a lack of integrated analysis of multi-omics datasets across multiple basal ganglia brain regions, very little is known about the regulatory mechanisms of this area.

Findings

We utilized high-throughput transcriptomic and epigenomic analysis to profile over 270,000 single-nucleus cells to create a cellular atlas of the basal ganglia, characterizing the cellular composition of four regions of basal ganglia in adult macaque brain, including the striatum (STR), substantia nigra (SN), globus pallidum (GP), and amygdala (AMY). We found a distinct epigenetic regulation on gene expression of neuronal and non-neuronal cells across regions in basal ganglia. We identified a cluster of SN-specific astrocyte associated with neurodegenerative diseases, and further explored the conserved and primate-specific transcriptomics in SN cell types across human, macaque and mouse. Finally, we integrated our epigenetic landscape of basal ganglia cells with human disease heritability, and identified a regulatory module consisting of candidate *cis*-regulatory elements that are specific to medium spiny neurons and associated with schizophrenia (SCZ).

Conclusions

In general, our macaque basal ganglia atlas provides valuable insights into the comprehensive

transcriptome and epigenome of the most important and populous cell populations in the macaque basal ganglia. We have defined over 50 cell types with transcriptomic and epigenomic profiles, some of which exhibit region-specificity, and characterized the molecular relationships underlying these brain regions.

Key words: Basal ganglia; single cell; snATAC-seq; snRNA-seq

Introduction

The basal ganglia and related nuclei are critical for motor control, motor learning, cognitive functions and emotional response[1]. The heterogeneous neuron distribution is underlying these diversities of physiological functions, exemplified by the best known medium spiny neurons in striatum and dopaminergic neurons in substantia nigra area. The functional organization of basal ganglia, especially the motor circuit is tightly related to the neurodegenerative diseases, such as Parkinson’s disease[2]. Single cell technology has dissected the cell taxonomy of basal ganglia and related areas in mouse and primate[3, 4]. Single-cell technologies have shown significant in assessing cell type-specific gene expression differences in several brain diseases, including Alzheimer's disease (AD), autism spectrum disorder (ASD), multiple sclerosis, and major depressive disorder (MDD) [5-8]. Identifying cell type-specific gene expression is crucial for associating cell identities and functions, and for associating/linking cell types and the genetic variation underlying psychiatric disorders such as SCZ [9]. While substantial progress has been made in understanding cell type heterogeneity across and within different regions of the basal ganglia, most reports were limited to single transcriptomics in a few brain regions, such as SN [10],

AMY [3], and STR [11]. However, the subregional heterogeneities in cell types, transcriptomics and epigenomics in basal ganglia have not been illustrated. Moreover, the regulatory state for specific cell types in basal ganglia and their correlation with neurological diseases still hasn't been well defined. It is important to note that the regulatory mechanisms behind the different cell types in different regions of the basal ganglia are vastly different [12], genome-wide chromatin accessibility sequencing is more sensitive in assessing cis-regulatory elements and disease-associated genetic risk loci [13] compared to single gene expression measurements. The specific cell types and regulatory mechanisms in different regions of the basal ganglia, as well as the association between cell types and disease risk, are still not well understood at present.

To better understand gene regulatory landscape of non-human primate basal ganglia, tissues of the basal ganglia regions (including SN, AMY, STR, and GP) were sampled from -two 72-month-old female macaque (Macaca fascicularis), followed by single-nucleus RNA-seq (snRNA-seq) and single-nucleus ATAC-seq (snATAC-seq). We defined 49 cell types by snRNA-seq and 47 cell types by snATAC-seq within the basal ganglia regions, uncovered their molecular features, and revealed the regulatory elements underlying the differences in gene expression among the cell types. We discovered region-specific subtypes of neurons and elucidated the heterogeneity of gene expression and regulatory mechanisms among these region-specific neurons. Additionally, we identified a group of SN-specific astrocyte subtypes associated with neurodegenerative diseases and revealed their transcriptional signatures. Simultaneously, we predicted the regulatory patterns of transcription factors significantly activated in these astrocyte subtypes on neurodegenerative disease-related genes. Furthermore, we systematically analyzed the cross-species conservation and primate-specific DEGs across different cell types of the SN region, and revealed a correlation between primate-

specific DEGs and neurodegenerative diseases. Finally, we identified celltype-specific chromatin-accessible sites co-localized with human trait-associated single nucleotide polymorphisms and plotted disease-associated open regions and gene enrichment related to human neurological diseases in the non-human primate (NHP) basal ganglia on a topographic map. Overall, our results provide a systematic analysis of the lineage of cellular composition, transcription, and regulation in various regions of the macaque basal ganglia. This study fills a gap in the basal ganglia epigenetic data and significantly expands our current understanding of the molecular basis of basal ganglia cell types. Comprehensive analysis of single-cell basal ganglia epigenomic data can help to understand the regulatory mechanisms of key genes in different cell types, study the cell-type preference of disease risk loci, and facilitate the identification of therapeutic targets for diseases. The basal ganglia cell atlas presented here offers a valuable resource for future research on model species.

Results

Single-nucleus transcriptional and chromatin accessibility profiling in macaque basal ganglia

The basal ganglia striatal caudate (Cd), putamen (Pu), AMY, SN and GP of two female macaque were obtained for single-nucleus RNA-seq (snRNA-seq) and single-nucleus ATAC-seq (snATAC-seq) sequencing, and the transcriptomic and epigenomic data were generated from the same tissue. After quality-control filtering, we obtained a total of 101,431 nuclei for snRNA-seq (20,104 from Pu, 36,451 from Cd, 31,219 from AMY, 9,386 from SN, and 4,271 from GP) (Methods, Supplementary Figure S1A, B and F and Supplementary Table S1) and 170,608 nuclei for snATAC-

seq (42,027 from Pu, 41,026 from Cd, 20,995 from AMY, 24,221 from SN, and 42,339 from GP) (Methods, Supplementary Figure S1C, D and F and Supplementary Table S1). To minimize differences between sample sources in snRNA-seq, we performed batch correction between samples. We then used uniform manifold approximation and projection (UMAP) to reduce the dimensionality of the snRNA-seq data. Subsequently, we employed original Louvain clustering to identify unique clusters of cell types. (Figure 1A and Supplementary Figure S1E). Based on the expression of marker genes for different cell types in previous studies, we have defined 17 major cell types and 49 subtypes[4, 10, 14-16]. These include excitatory neurons (EX, *SLC17A6*+/*SLC17A7*+), inhibitory neurons (IN, *GAD1*+/*GAD2*+, IN_SST interneurons (*SST*+), IN_PVALB interneurons (*PVALB*+), IN_TAC3 interneurons (*TAC3*+), IN_OTX2 interneurons (*OTX2*+/*CHRNA3*+), IN_LAMP5 interneurons (*LAMP5*+), IN_VIP_CCK interneurons (*VIP*+)), IN_CHAT interneurons (*CHAT*+)), medium spiny neurons (MSN, *PPP1R1B*+), dopaminergic neurons (DaNs, *TH*+), astrocytes (AST, *AGT*+), oligodendrocytes (OLIG, *MOG*+), oligodendrocyte precursor cells (OPC, *PDGFRA*+), microglia (MIC, *C1QA*+), and endothelial cells (ENDO, *FLT1*+). (Figure 1C)

For snATAC-seq, we processed the data using the ArchR software package [17] to obtain a low-dimensional result through an iterative approach. Then a consensus set of 657,930 accessible peaks representing potential cis-regulatory elements (cREs) based on preliminary clustering results was obtained. To ensure consistency between the two technical sources in cell types, we extracted peak and gene score matrices and used SeuratV4 to establish anchors between cells from different technical sources by gene score and gene expression matrices, then integrated the two types of dataset by mapping snATAC-seq cells to the low-dimensional space of snRNA-seq (Figure 1E and

F).We annotated the major cell types for snATAC-seq using the labels from snRNA-seq results excluding the cells with prediction scores below 0.6. We utilized the Signac package to perform UMAP reduction and batch correction on the data, followed by the utilization of smart local moving (SLM) clustering for analyzing the snATAC-seq datasets (Figure 1B and Supplementary Figure S1E). The predicted cell types were validated by the increased accessibility in promoter of markers genes for the corresponding major cell types (Figure 1D).

Next, we performed an unsupervised clustering analysis of major cell types in snRNA-seq and snATAC-seq data. In total 49 subtypes revealed by snRNA-seq and 47 subtypes revealed by snATAC-seq based on differences in marker gene expression or chromatin accessibility. (Supplementary Figure 1A and B, Figure 2, Figure 3, Supplementary Figure 3 and Supplementary Figure 4). We further identified a differential accessible cRE (DA cRE) set for the snATAC-seq subtypes. We found that the differences between neuronal subtypes were greater than those between non-neuronal subtypes (Supplementary Figure S1G). Using ArchR, we linked distal cRE accessibility to gene expression to identify 109,506 cRE-gene pairs representing potential enhancer-gene interactions (Methods, Supplementary Table S3). The co-variation of cRE accessibility and gene expression distinguished cell types identified in snRNA-seq and snATAC-seq (Figure 1G). Clustering of cRE accessibility revealed cell type-specific variability, confirming the similarity of neuronal subclusters and heterogeneity across neuronal and non-neuronal subtypes, and indicating dynamic modes of gene regulation across inhibitory neuron clusters.

Next, we used Chromvar to calculate the enrichment level of transcription factor (TF) binding motifs

in chromatin open regions of each snATAC-seq cell and evaluated the cell type-enrichment of TF binding motifs (Figure 1H). The TFs with enriched binding motif in these cell types are functionally related to their respective cell types. For example, NEUROD2, enriched in EX, is an effecter TF expressed in the cortical projection neuron lineage during the peak of cortical excitatory neurogenesis [18], and is crucial for the development of the AMY [19]. JUNB and FOS families, enriched in MSN, are associated with MSN desensitization [20]. EMX1 and EMX2, enriched in IN_CHAT, and in the absence of EMX2 and PAX6, EMX1 might have inhibited basal ganglia morphogenesis [21]. The transcription factors ASCL2, and TCF3, which are enriched in the IN_PVALB, IN_SST, and IN_TAC cell types, are involved in the development, proliferation, and differentiation of neurons and neural progenitor cells[22, 23], suggesting a crucial role in controlling the formation and function of these specific types of inhibitory neurons. The AST cell type is enriched with the MSX2 binding motif, where this TF expressed in a time-dependent manner in glial cells after injury [24], while the OPC-enriched BBX may be involved in central nervous system development [25], and SOX9 regulates oligodendrocyte differentiation [26]. It has been suggested that the SPIB TF may be an important regulatory factor for MIC sensing function [27], and FOXF2 has been shown to be involved in the development and maintenance of the ENDO blood-brain barrier [28]. It is worth noting that NR4A2 is highly expressed in DaNs. The expression of nr4a2 is crucial for the early differentiation of dopamine neurons, and its expression in adulthood is necessary for dopamine to carry out its function[29].

Heterogeneity of gene expression and transcriptional regulation in basal ganglia neurons

Based on the gene expression heterogeneity among neurons, we further investigated the feature of neuron subtypes in basal ganglia. The 83,206 neuronal cells revealed by snRNA-seq were categorized into 26 subtypes, as shown in Figure 2A, B, and D. These comprise 6 subtypes of excitatory neurons, namely EX_PALMD, EX_HTR2C, EX_CBLN4 (including two subtypes, EX_CBLN4-1 and EX_CBLN4-2), EX_ZMYM4, and EX_APOC1B. Additionally, there are 13 subtypes of inhibitory neurons: IN_SST (IN_SST-1 and IN_SST-2), IN_PVALB (IN_PVALB-1-4), IN_TAC3, IN_OTX2_CHRNA3, IN_LAMP5 (IN_LAMP5-1-3), IN_VIP_CCK and cholinergic neurons (IN_CHAT). Moreover, the data also revealed the presence of 2 subtypes of dopaminergic neurons (DaNs, which included DaNs-1 and DaNs-2), and 5 subtypes of medium spiny neurons D1_MSN (D1_MSN_Matrix and D1_MSN_Striosome), D2_MSN (D2_MSN_Matrix and D2_MSN_Striosome) and D1_D2_hybrid MSN.

We performed differential gene expression analysis among the 26 neuronal subtypes. The top DEGs of the EX_PALMD excitatory neuron include *CAMK2D* and *PDE1A*, which encode calcium/calmodulin dependent protein kinase II delta1 and phosphodiesterase 1A proteins, respectively. The EX_HTR2C neuron expressed high levels of *HTR2C*, (5-hydroxytryptamine receptor 2C protein), which is a G protein-coupled receptor (*GPCR*) that couples with Gq/G11 and mediates excitatory neural transmission [30]. This group of neurons also expressed high levels of *TRPM3*, (transient receptor potential cation channel subfamily M member 3 protein), that may play a role in regulating the excitability of these neurons [31]. *CBLN4* is a member of a small secreted protein family that contains a C1Q domain, and members of this family participate in the regulation

of neurexin signaling during synaptic development [32]. We defined two groups of excitatory neurons that showed high expression of the *CBLN4* gene, which we named EX_CBLN4-1 and EX_CBLN4-2. EX_CBLN4-1 was found to express top markers genes of *TLL1* and *NDST4*, which encode an astacin-like, zinc-dependent, metalloprotease, and an N-deacetylase and N-sulfotransferase, respectively. EX_CBLN4-2, on the other hand, was identified as a type of cell that showed high expression of *NEFL*, *NEFM*, and *NEFH*, which encode the heavy, medium, and light chains that make up neurofilaments. High expression of the similar neurofilament-associated signals was also found in the mouse basal ganglia [14, 33, 34]. EX_ZMYM4 neurons expressed high levels of *ZMYM4*, a zinc finger protein, plays a role in regulating cell morphology and cytoskeletal organization [35], while also expressing *FAM19A1*, a member of a conserved chemoattractant-like protein family abundant in the mouse and human central nervous system [36]. EX_APOC1B neurons expressed high levels of *APOC1B* and *APOE*, which encode Apolipoprotein C-I and Apolipoprotein E, respectively.

In addition to snRNA data, we mapped snRNA-seq neuronal cell types to snATAC-seq neuronal cell types by label-transfer, as described above. In the snATAC-seq data, we identified four types of excitatory neuronal subtypes (EX_PALMD, EX_HTR2C, EX_CBLN4−1 and EX_ZMYM4) (Figure 2C), and enriched with gene scores for marker genes of corresponding transcriptomic cell types (Figure 2D). Our analysis on both snRNA-seq and snATAC-seq data revealed that almost all EX_PALMD, EX_HTR2C, and EX_ZMYM4 were present in the amygdala, while EX_CBLN4-1 and EX_CBLN4-2 were restricted to the substantia nigra. Additionally, we observed that 88.9% of the snRNA-seq EX_APOC1B neuron were found in the amygdala, while the remaining

EX_APOC1B neurons were found in the striatal caudate nucleus.

We identified two distinct subpopulations of IN_SST neurons which exhibited different molecular features and epigenetic characteristics. The majority of IN_SST-1 cells were found in the amygdala, whereas IN_SST-2 neurons were primarily distributed in the striatum (Figure 2A, Figure 2C and Supplementary Figure S2A). Two subtypes of SST neurons displayed consistent nuclear distribution patterns in both RNA-seq and ATAC-seq data. Moreover, the two types of IN_SST neurons exhibit heterogeneity in terms of gene expression and regulation in snRNA-seq and snATAC-seq data, respectively (Figure 2E). IN_SST-1 expressed high levels of neurexophilin-1 (*NXPH1*), while *NPY* is the marker for IN_SST-2 [37]. Genes involved in regulating monoatomic ion transmembrane transport, such as *ASIC2*, *ADCYAP1R1*, *DPP10*, and *RASGRF2*, and genes related to glutamate receptor signaling pathways, such as *GRIA1*, *GRIA3*, *GRID1*, and *GRIK1*, are highly expressed in IN_SST-1. Whereas, the highly expressed genes in IN_SST-2, such as *NPY*, *LHX6* and *RELN*, were related to the regulation of central nervous system neuron development and axon guidance (Supplementary Figure S2B). To explore the different transcriptional regulation between IN_SST neuron subtypes, we used snATAC-seq data to calculate the activity of TF binding motifs in IN_SST-1 and IN_SST-2 using Chromvar. We found that motif activity and significantly increased targeted gene scores of *TFAP4*, *NHLH2*, *ASCL2*, *TCF21*, and *ZNF238* were present in IN_SST-1, while increased motif activity and targeted gene scores of *POU5F1*, *SNAI2*, *POU2F3*, *POU2F1*, and *NR1D1* were found in IN_SST-2 (Supplementary Figure S2C). Next, we utilized cRE-gene link and TF binding motif database to establish the TFs regulated genes network of IN_SST neurons. We identified cell type-specific DEGs in TF-targeted genes (Supplementary Figure S2D). We found that

for the TFs with enriched binding motifs in IN_SST-1, the majority of the DEGs linked to them are from IN_SST-1 (*P* value < 0.001), while for the TFs with enriched binding motifs in IN_SST-2, the majority of the DEGs linked to them are from SST2 (*P* value < 0.001). These findings suggested that the regulatory role of these transcription factors on the differential gene expression and related functions of IN_SST subtypes.

IN_PVALB neurons exhibit pan expression of the PVALB gene, can be further classified into 4 subtypes, designated as IN_PVALB1-4 in snRNA-seq and snATAC-seq. IN_PVALB-1 neurons were mainly distributed in the globus pallidus and substantia nigra, while IN_PVALB-2 were mainly found in the striatum (including the striatal putamen and the striatal caudate). Most of IN_PVALB-3 is distributed in globus pallidus, followed by Pu. IN_PVALB-4 neurons were almost restricted to the amygdala. We then performed the differentially expressed gene analysis and the gene ontology enrichment analysis among PVALB subtypes (Supplementary Figure S2E and S2G). We found that the enriched pathways in the DEGs of IN_PVALB-1, IN_PVALB-2, and IN_PVALB-4 largely overlap, mainly including axon development, axonogenesis, and synapse organization. In addition to modulation of chemical synaptic transmission, IN_PVALB-3 is also involved in pathways such as ATP synthesis coupled electron transport, mitochondrial ATP synthesis coupled electron transport, etc. (Supplementary Figure S2G) Next, we explored the differential chromatin accessibility of PVALB subtypes and noted their high cell type specificity (Supplementary Figure S2F). In summary, PVALB subtypes exhibit significant differences in both the epigenome and transcriptome, suggesting that these subtypes may have distinct functions.

Transcriptional and regulatory heterogeneity of MSN subtypes.

GABAergic medium spiny neurons (MSNs) are specific neuronal population located in the striatum of the basal ganglia, representing 95.0% of the neurons in this region [38]. MSNs can be divided into two main subtypes: DRD1-MSN and DRD2-MSN. DRD1-MSNs project directly to the interface nuclei between the basal ganglia and the rest of the brain, while DRD2-MSNs project to the intermediate basal ganglia nucleus, which is indirectly connected to the interface nuclei [39]. We found that macaque DRD1-MSN (46.4% of MSN) selectively expressed *DRD1* and *TAC1* genes (Figure 2B), which encode substance P, neurokinin A, neuropeptide K, and neuropeptide gamma, and stimulate the output structure of the basal ganglia [38]. DRD2-MSN (53.6% of MSN) specifically expressed the *DRD2* gene (Figure 2B), encoding the D2 subtype of the dopamine receptor, which inhibits the output structure of MSNs [38]. Consistent with the transcriptional MSN subtypes found in macaque striatum, we defined DRD1_MSN_Matrix and DRD2_MSN_Matrix using *STXBP6*, and DRD1_MSN_Striosome and DRD2_MSN_Striosome using *KCNIP1*. Additionally, we defined D1_D2_hybrid using *GRIK1*, which was expressed in both DRD1 and DRD2 in the snRNA-seq.[4] Similarly, in the matched MSN subtypes in snATAC-seq, we found that these marker genes had highly specific gene activity. (Figure 2F and Supplementary Figure S3A). Genes with high-density cRE-gene-associated regions were referred as domains of regulatory chromatin (DORCs), and these genes were enriched in some known super-enhancers, indicating their crucial regulatory significance in determining cell identity[40]. In light of this, we aimed to investigate which DEGs in the MSN subtype may have regulatory importance. We defined 36 DEGs

with a large number (>10) of cREs as DORC based on our established cRE-gene pairs, (Figure 2G). Consistent with previous studies, gene activity of most DORCs had the same cell-type specificity as gene expression (Figure 2H and Figure 2I)[40]. Furthermore, we found that some DORCs, such as *CNR1*, *HOMER1*, *ADORA2A*, and *DRD2*, have been reported to be downregulated in Huntington's disease patients in the striatum[41]. Our data showed that *CNR1* and *HOMER1* were highly expressed and active in DRD1-MSN, while *ADORA2A* and *DRD2* were highly expressed and active in DRD2-MSN. These results suggest that these DORCs, especially those downregulated in disease, have significant research implications. In addition to analysis of cis-regulatory elements in MSN subtypes, the regulatory function of TFs is of great importance in determining cell identity. Therefore, we systematically calculated the differential TF motifs activity in MSN subtypes using snATAC-seq (Supplementary Figure S3B). We found that the TF NPAS2 had high activity in D1_D2_hybird (Figure 2J). The core circadian protein NPAS2 has been reported to negatively regulate the nucleus accumbens, and disruption of NPAS2 produces augmented cocaine preference[42]. By establishing a TF regulatory network (Method), We found that genes regulated by NPAS2 were significantly more highly expressed in the D1_D2_hybrid MSN subtype in snRNA-seq compared to other MSN subtypes(Figure 2J). These results further highlight the importance of NPAS2 in the striatum.

It is interesting to note that a similar group, known as eMSN, has been found in the mouse striatum, which shares similarities with the D1_D2_hybrid group [14]. When integrated snRNA-seq with mice MSN data, we found that our DRD1-MSN, DRD2-MSN, and D1_D2_hybrid MSN all have high correlation with mouse MSN1, MSN2, and eMSN (Supplementary Figure S3C). Compared with DRD1-MSN and DRD2-MSN, the D1_D2_hybrid subtype had the most

differentially expressed genes (86 of 118 DEGs in mouse, 152 of 179 DEGs in macaque) (Supplementary Figure S3D). The D1_D2_hybrid subtypes of the two species shared 22 common DEG ($P = 5.35\times10^{-25}$ by hypergeometric test), including *PBX1*, *PBX3*, *TSHZ1* and *OLFM3*, which were involved in sensory organ development (GO:0007423, $P = 0.36\times10^{-4}$) and visual system development (GO:0150063, $P = 1.6\times10^{-3}$). Macaque has 130 diverged DEGs, including genes involved in the neuroactive ligand-receptor interaction (e.g. *GRIK1*, *TACR1*, and *RXFP1*), and genes , involved in cell morphology and neuron differentiation pathways (e.g. *APP*, *EDN1*, *EPHA4*, *EPHA5*, and *STMN1*), supporting the ideas that primate MSN neurons have higher complexity in signal transduction function (Supplementary Figure S3E and F)[43].

Molecular specialized astrocyte subtypes across basal ganglia

Astrocytes, located in the central nervous system (CNS), are responsible for a range of functions including transmitting nerve signals, promoting synaptic genesis and transmission, and repairing damage to neurons[44-46]. Dysfunction of astrocytes is implicated in various neurodegenerative diseases[47-49]. Therefore, identifying and characterizing the subtypes of astrocytes can aid a better understanding of their molecular basis for functions. Out of the 23 non-neuronal cell types in snRNA-seq, 5 astrocyte subtypes were identified (Figure 3A, B, and Supplementary Figure S4A). We found nucleus specific distribution of AST subtypes. For instance, AST4 was restricted to the substantia nigra, AST5 was mostly found in the amygdala, and AST2 only existed in the striatum, including the caudate nucleus and putamen (Figure 3A and Supplementary Figure S4C). Then, we

performed the gene module analysis based on top 5000 variable features of AST, and further correlated the 5 gene modules to the corresponding AST subtypes (Figure 3C). Gene ontology analysis of enriched genes in each module revealed that module 1 (corresponding to AST2, Supplementary Figure S4B) and module 5 (corresponding to AST1, Supplementary Figure S4B) were related to known AST functional pathways, such as neural projection, transmembrane transport, synaptic transmission, and regulation of cell morphogenesis, etc. [50, 51].

Involvement of astrocytes in the immune response has been reported in previous studies [48, 49]. We found that the genes in module 4 (AST3), such as *CX3CR1*, *CSF1R* and *IL18*, were related to inflammatory responses, supporting the idea that astrocytes were associated with inflammation. Similarly, we also found high expression of corresponding genes in AST subtypes in mice [31]. Astrocytes have been implicated in neurodegenerative disorders and may contribute to striatal neuron loss or dysfunction in Huntington's disease (HD) [50]. High expression of dynein axonemal heavy chain genes (including *DNAH3*, *DNAH5*, *DNAH7*, *DNAH6*, *DNAH9*, *DNAH11*) were found in module 2 (AST5) (Supplementary Figure S4D). Mutations in these genes can cause striatal atrophy and impair axon growth of striatal neurons, possibly leading to Huntington's disease [52].AST4 is a specific subtype of astrocytes only found in the substantia nigra. It expressed high levels of *ALDH1A1*, *DDC*, and *SLC6A3* genes, which are associated with the function of gene module 3 involved in dopaminergic neurogenesis (WP2855, P = 4.7×10^{-11})[53]. Additionally, AST4 expressed high level of *MAP3K5*, *MAPK10*, and *TUBB3*, which are enriched in Alzheimer's disease-related pathways (hsa05010, P = 2.0×10^{-07}) (Figure 3D)[54, 55]. Consistently, we also identified a group of substantia nigra-specific epigenetic AST subtype AST3(A) in the snATAC-seq data

(Supplementary Figure S4C), which had a high correlation with transcriptomic AST4 cells in the snRNA-seq data (Figure 3F). We found that the binding motif activities of transcription factors RFX4 and PBX3, specifically expressed in AST4, were significantly high in AST3(A) (Figure 3E, G), suggesting that role of RFX4 and PBX3 in regulating specific transcriptomics of AST4[56]. In addition, by overlaying the cRE-gene co-variability map and TF binding motif data along the genome axis, we revealed a potential *cis*-regulatory relationship of pathogenic genes specific to AST4, such as *SLC39A12*, in neurodegenerative diseases such as Alzheimer's disease (Figure 3H)[57].

Transcriptional regulation on species-conserved and species-divergent genes in substantia nigra cells

The SN is considered as the primary input region of the basal ganglia, and its dysfunction is implicated in a set of neurological disorders, including Parkinson's disease, Huntington's disease, schizophrenia, and obsessive-compulsive disorder [58]. DaNs originating from the SN play a critical role in movement, cognition, emotion, and reward processes, and their dysfunction is a hallmark of neurodegenerative diseases [59]. To better understand the species-conserved and divergent transcriptomics in the SN cells between rodents and primates, we integrated single-cell datasets of excitatory neurons, inhibitory neurons, dopamine neurons, and non-neuronal cells from the SN of human, macaque and mouse [10, 14] (Figure 4A, and Supplementary Figure S5A, B). We demonstrated that the major cell types identified by SN region across species were conserved. For each species, we calculated DEGs in the major cell type, and compared DEGs of the same cell type.

Our analysis revealed that 16 to 257 cell type-DEGs are conserved across species, and a greater number of cell type-DEGs is shared between human and monkey (Figure 4B, C). To further explore the regulatory mechanisms of these species-conserved DEGs, we used corresponding snATAC-seq data of macaque to depict the gene activity scores of species-conserved DEGs, as well as the linked differentially accessible cis-regulatory elements (DA cREs) (Figure 4D). To reveal potential TF that may modulate these species-conservative genes, we identified binding motifs enriched in the cell type DA cREs linked to these conserved DEGs. (Figure 4E). For instance, motifs enriched in neurons included ATF3, FOSL1/2, JUNB, and BATF, while motifs enriched in astrocytes (AST) included NFIX and NF1, which are crucial for the maturation and growth of AST [60, 61]. Motifs enriched in OLIG were associated with the SOX10 function, which is necessary for the survival of oligodendrocytes that wrap axons to form myelin sheaths [62]. Finally, motifs enriched in endothelial cells (ENDO) and microglia (MIC) included ETS1, IRF2, SPIB/1, ELF4, and ETV1. In summary, we characterized these DEGs as cross-species conserved at the transcriptional level, and identified the candidate transcription factor that may exert the transcriptional regulation on those genes [63].

DEGs shared by human and macaque but not found in mouse are considered as primate-specific DEGs. In our analysis, we discovered that DaNs harbored the highest number of primate-specific DEGs (n = 289), followed by OLIG (n = 131), AST (n = 125), OPC (n = 56), MIC (n = 37and ENDO (n = 26) (Figure 4B). We then examined the pathways involved in the primate-specific DEGs, such as primate-specific DEGs in DaNs were enriched in pathways involved in ion transmembrane transport, neurodegeneration and Parkinson's disease (Supplementary Figure S5C). As our prior

research indicated that the substantia nigra-specific AST4 is involved in neurodegenerative diseases (Figure 3D). We found that primate-specific DEGs were enriched in different pathways in AST of SN, including *APC*, *ITPR2*, and *PIK3R1*, which are related to Alzheimer's disease, as well as *ERBB4*, *FGFR1* and *FYN*, which are associated with diseases related to signal transduction by growth factor receptors and second messengers (Figure 4F)[64, 65]. To gain insight into the regulatory mechanisms of primate-specific genes in the AST of SN, we focused on key TFs involved in transcriptional regulation. We observed specific gene expression of transcription factors NFIA, RFX4, and ARID2 in the AST of SN, which are associated with AST maturation [66], and found higher enrichment of their binding motifs in AST from SN (Figure 4G). By utilizing the potential regulatory relationships of the TF gene, an AST high-activity transcription factor regulatory network was constructed. Furthermore, the pathways related to the enrichment of primate-specific DEGs in AST cell types were identified and labeled within the regulatory network. (Supplementary Figure S5D). We observed that the *FGFR1* gene in the diseases of signal transduction by growth factor receptors and second messengers pathway contained an NFIA and RFX4 motif binding site in its cRE. This revealed that the transcription factor NFIA and RFX4 may regulate the expression of the *FGFR1* gene through multiple motif binding sites (Figure 4H).

Cell-type specific *cis*-regulatory risk loci of human traits and diseases

Disease risk loci identified in genome-wide association studies (GWAS) show different degrees of enrichment in various cell type-specific regulatory elements. However, the lack of epigenetic data in previous basal ganglia datasets has resulted in a dearth of information on disease risk loci enriched

in cell type-specific regulatory elements in the basal ganglia. To fill this gap, we mapped all coordinates of DA cREs from each subcluster to the orthologous coordinates in the human hg19 genome, then performed linkage-disequilibrium score regression (LDSC) analysis using GWAS summary statistics for human traits and diseases on the DA cREs [67](Methods, Supplementary Table S4).

We evaluated the enrichment of risk loci for neurological diseases and other human traits in basal ganglia cell types. We found that risk loci for human neurological diseases and related traits was mainly enriched in neuronal cells (Figure 5A). IN_PVALB-2 neurons showed significant enrichment of sites for major depressive disorder (MDD) (*P* value = 3.1×10^{-3}). PVALB neuron in the amygdala appears to be particularly susceptible to the effects of chronic stress which is considered as the primary risk factor for MDD [68]. In addition, IN_CHAT neurons showed correlation with MDD (*P* value = 3.2×10^{-2}), in line with previous findings in mice that IN_CHAT regulates depression-like behavior [69]. We found significant enrichment of SCZ-associated sites in IN_SST-2 neurons (*P* value = 1.8×10^{-6}), and the reduction of SST neurons was found in SCZ patients [70]. Notably, BD-associated loci were significantly enriched in all subtypes of MSNs (*P* value = 4.0×10^{-5}, 4.1×10^{-5}, 2.0×10^{-5}, 5.1×10^{-7} and 8.6×10^{-5} for D1_MSN_Matrix, D1_MSN_Striosome, D2_MSN_Matrix, D2_MSN_Striosome and D1_D2_hybrid). Transcriptome analysis in striatum tissue have connected the co-expressed gene module of BD patients and control to MSN neurons [71]. Our findings suggest that investigating neuronal subtypes is a promising avenue for studying neurological diseases.

Furthermore, we looked into the specific disease-associated loci that overlapped with cell type enriched cREs. Significant enrichment of SCZ-associated sites was observed in D1_MSN_Matrix, D1_MSN_Striosome, D2_MSN_Matrix, D2_MSN_Striosome and D1_D2_hybrid in MSN neurons (*P* value = 1.1×10^{-4}, 2.8×10^{-4}, 5.8×10^{-5}, 4.0×10^{-6} and 2.6×10^{-3} for D1_MSN_Matrix, D1_MSN_Striosome, D2_MSN_Matrix, D2_MSN_Striosome and D1_D2_hybrid), in line with previous findings that genomic variants associated with SCZ map to MSN[9]. Subsequently, we found the enrichment of cREs in MSN subtypes that located in the SCZ-associated variant locus rs12293670 (Figure 5B). SCZ risk variant rs12293670 has been attributed to the candidate gene *NRGN*, which exhibits specific expression in the human brain [72]. By overlaying peak-gene co-variable maps with chromatin accessibility signal, we found the gene *NRGN* showed strong correlation with rs12293670 in MSN subtypes, moreover, we found the enriched gene expression of *NRGN* in transcriptomic MSN subtypes (Figure 5C). Our results proposed medium spiny neurons as potential pathological and therapeutic targets for the future SCZ studies.

By integrating human GWAS data and macaque cell type transcriptomic and chromatin accessibility data, we further attribute the risk loci and related genes to the specific cell type and provide the potential pathological and therapeutic candidates.

Discussion

In this study, we utilized snRNA-seq and snATAC-seq techniques to profile the striatum, substantia nigra, globus pallidus, and amygdala in the macaque basal ganglia. While several studies have

generated diverse datasets of the basal ganglia at the single-cell transcriptomic level [4, 10, 73], molecular architecture in basal ganglia have not been extensively explored at the level of sample diversity, multi-omics, and transcriptional regulation of specialized molecular cell types in the complex basal ganglia region. Non-human primates are highly evolutionarily related to humans and possess comparable brain structures, which make them valuable animal models for studying human diseases [74]. In order to elucidate the pathogenesis of various diseases and prioritize potential therapeutic strategies, it is crucial to understand the cellular composition and molecular regulation of the macaque basal ganglia at both the transcriptomic and epigenomic levels. Our research represents the first comprehensive exploration and comparison of multiple basal ganglia regions in the macaque at the single-cell level of transcriptomics and epigenomics. In conclusion, our study provides a comprehensive data resource for non-human primate disease models related to basal ganglia areas, as it demonstrates the diverse and regional patterned regulatory network in basal ganglia cells.

The high-throughput single-cell sequencing technology provides an unprecedented opportunity for investigating the cellular composition and molecular features of the basal ganglia. By analyzing over 101,431 snRNA-seq and 170,608 snATAC-seq data from the macaque basal ganglia, we characterized the transcriptional and epigenetic features of neuronal cell types, including EX, IN, MSN, and DaNS, as well as non-neuronal cell types such as AST, OLIG, OPC, MIC, and ENDO. By further classifying major cell types based on different marker genes and accessible chromatin landscapes, we identified 52 subpopulations in snRNA-seq data and 50 subpopulations in snATAC-seq data. By converting chromatin accessibility data to gene activity data, we matched the neuronal

subtypes from snATAC-seq to their corresponding counterparts in snRNA-seq neuronal subtypes, and validated the consistency of cell identity through marker gene expression and activity. In this way, we were able to conduct in-depth analysis of the molecular basis of differential gene expression between these neuronal subtypes.

The basal ganglia primarily coordinate complex motor behaviors in the body by suppressing signal output. To gain a deeper understanding of the composition and regulatory mechanisms of inhibitory neurons in the basal ganglia, we further conducted a detailed comparative study at the molecular level to investigate the differences between subtypes of inhibitory neurons. We discovered SST heterogeneity in the amygdala-specific neuronal subtype and identified potential regulatory mechanisms that may account for differences in gene expression. We also identified PVALB subtypes that were region-specific and demonstrated differences in transcriptional and epigenetic regulation, as well as significant functional differences between subtypes within the same neuron. Comparing the macaque and mouse MSNs [31], we discovered that our D1_D2_hybrid cells were the same population of cells as eMSN in mice, eMSN was a “eccentric” MSN that significantly differed from the classic MSN in mice. We found that even within the same eMSN cell type, there were significant differences between species. Given the importance of MSNs and the specificity of eMSNs, this group should be included in further research.

Astrocytes are abundant glial cells in the central nervous system that provide nutrition and support for neurons. The study of astrocyte subtypes is crucial for understanding astrocyte-associated diseases due to their functional heterogeneity between and within brain regions[75]. Recently, the

distinct subtypes of AST in the cortex and subcortical regions of mice have been revealed [76]. We classified our ASTs into five and seven subtypes in snRNA-seq and snATAC-seq data, respectively. Gene module analysis revealed that a group of SN-specific transcriptomic AST4 is mainly associated with neurodegenerative diseases [77]. We also identified a cluster, AST3in our snATAC-seq data, that corresponds to AST4 in snRNA-seq data. Through integrated analysis of transcriptomics and epigenomics, we have gained a deeper understanding of the regulatory mechanisms underlying the SN-specific gene expression of this group of AST subtypes, providing a foundation for future disease research.

Based on the importance of SN in Parkinson's disease [78], we compared SN cell types among three species (humans, macaques, and mice) to gain insight into the fundamental molecular regulatory mechanisms diverged through evolution [79]. We also screened for DEGs specific to primates, which is essential for understanding the susceptibility to primate-specific and rare diseases [80]. We found that the DEGs conserved across species are mostly classical marker genes for cell types. Moreover, the DEGs conserved across species also exhibit significant differences in chromosomal activity between cell types. This suggests that in the future, the regulatory mechanisms of conserved DEGs across species can be studied through differences in transcription factor activity and chromatin accessibility between cell types [81]. Previous studies have reported the association of CNTN2 with OLIG differentiation [82]. Similarly, our prediction indicates that SOX10 regulates the conserved OLIG DEG CNTN2 gene, suggesting potential similarities in OLIG differentiation across different species.

We systematically analyzed primate-specific DEGs in AST cell types and found that highly active transcription factors, such as NFIA, RFX4, and ARID2, may regulate primate-specific DEGs enriched in different pathways (Supplementary Figure S4D). Our proposal suggests that NFIA and RFX4 regulate PPFIA1 by controlling the cREs of the PPFIA1 gene. Additionally, based on the importance of DaNs, we performed GO functional enrichment analysis of primate-specific DEGs in DaNs and obtained a series of genes associated with neurodegenerative diseases. This may provide further research directions for studying neurodegenerative diseases using non-human primate models.

Moreover, by combining epigenomic data with GWAS loci, disease risk can be linked to specific cell types. We found significant differences in the enrichment of different neuronal subtypes for disease, suggesting targeted investigation of specific cell types for different diseases. Additionally, by integrating our epigenomic and transcriptomic data, we identified a potential cis-regulatory relationship between *NRGN*, specifically expressed in MSNs, and SCZ GWAS loci. Linking diseases to relevant cell types and predicting regulatory sites for disease-associated genes can provide potential targets for future disease treatments.

Although we provided relatively comprehensive single-cell transcriptomic and epigeneticdata of basal ganglia, there were still some limitations in our research. Firstly, only two female monkeys were included in the analysis, so our analysis could not incorporate gender differences and might have overlooked basal ganglia-specific information in adult male macaques. Nevertheless, there was a good match between cell types in different regions of the two monkeys. Secondly, the high

resolution of singleome snATAC-seq and snRNA-seq enables the generation of high-depth and high-throughput single-cell results. However, compared to multiome approaches such as snRNA-seq and snATAC-seq to study the same cells, there may be inherent sequencing technology biases and filtering criteria that result in differences in the number of cells obtained from singleome snRNA-seq and snATAC-seq in the same sample. Additionally, while algorithms can be used to match cell identities between singleome snRNA-seq and snATAC-seq, there may be limitations in these algorithms, potentially missing some cells in snATAC-seq that have less matching with snRNA-seq. However, the large number of cells with matching identities between snATAC-seq and snRNA-seq helps alleviate this issue. Furthermore, previous studies have shown a high overlap between the cREs-gene pairs established by singleome and multiome approaches[83]. This indicates that our transcriptional regulatory research is reliable. In the future, we anticipate that the use of high-depth multiome technologies will allow for the generation of more comprehensive transcriptional regulatory landscapes in the basal ganglia region.

In summary, our macaque basal ganglia cell atlas provides valuable insights into the comprehensive transcriptome and epigenome of the diverse and functional related cell populations in the macaque basal ganglia. This information will serve as a foundational resource for future preclinical investigations on neurological disorders related to basal ganglia.

Methods

Ethics statement

The study was carried out in accordance with regulations on animal research and was approved by

the institutional review boards on the ethics committee of BGI (permit BGI-IRB A21025-T1)

Sample Preparation and Single-Cell Nucleus Isolation

Tissue samples were collected from the basal ganglia of two 72-month-old female crab-eating macaques (Macaca fasicularis) and immediately frozen in liquid nitrogen. The samples from both monkeys included the caudate nucleus (Cd), putamen (Pu), substantia nigra (SN), and globus pallidus (GP), while only one monkey (MK2) had a sample from the amygdala (AMY). The collected samples were subsequently processed for single-nucleus RNA-seq and snATAC-seq analysis.

As previously described [1], the method for single-nucleus preparation involved placing the frozen monkey brain tissue block into 1 mL of pre-chilled Dounce homogenization buffer and homogenizing it using 10 loose strokes and 10 tight strokes with the Dounce homogenizer, which was immersed in ice. Then, 2 mL of homogenization buffer was added to the Dounce homogenizer, and the homogenate was filtered through a 40-μm cell strainer (Miltenyi Biotech) into a 15-mL conical tube. Finally, the sample was centrifuged at 900 g for 10 minutes to pellet the cell nuclei. The overall quality and quantity of the isolated nuclei were estimated using fluorescence microscopy.

Library Preparation and Sequencing for scRNA-seq and snATAC-seq

To prepare the snRNA-seq library for the DNBelab C4 RNA-seq and C4 scATAC-seq analysis based on droplet technology, the DNBelab C series single-cell library preparation kit (MGI, #1000021082) and DNBelab C Series Single-Cell ATAC Library Prep Set (MGI, #1000021878) were employed.

As previously described [2], single-cell nuclear suspension was prepared from obtained mononuclear RNA samples, followed by 2 washes with PBS (containing 0.04% BSA). The nuclear suspension was then resuspended, filtered through a 40 μm cell strainer, and the cell suspension concentration was measured and recorded, followed by measurement and recording of the nuclear concentration. DNBelab C series single-cell library preparation kit (MGI, #1000021082) was used to prepare the nuclear suspension into droplets, which completed cell lysis and mRNA capture by magnetic beads in the droplets. Next, the single-cell magnetic beads were recovered using a lysis reagent recovery system (vacuum pump required), and the magnetic bead-captured mRNA was transcribed into cDNA. The cDNA was then subjected to double-stranded synthesis, followed by amplification and screening of the obtained cDNA and Oligo products. Subsequently, PCR was used to barcode the Oligo products for subsequent preparation into Oligo on-machine libraries. Finally, the cDNA product was fragmented, end-repaired, connected, PCR-amplified, denatured, circularized, and digested to prepare a single-stranded DNA library. After library preparation, the library was sequenced using the DIPSEQ T1 sequencing platform of the China National GeneBank (Shenzhen).

To perform snATAC-seq, as previously described [4], pre-extracted nuclei were subjected to a transposition reaction, followed by droplet generation using a syringe according to the protocol of the DNBelab C Series Single-Cell ATAC Library Prep Set (MGI, #1000021878). Next, demulsification, enzyme treatment, and PCR amplification reactions were conducted, and finally, a circular library was constructed and subjected to sequencing.

Processing and Quality Control of RNA-Seq Data

We performed filtering and demultiplexing of DNBelab C4 RNA-seq raw sequencing reads using PISA (RRID:SCR_015749) (version 0.7). Next, we employed STAR (RRID:SCR_004463) (version 2.6.1a) to demultiplex these reads. For alignment, we used a modified GTF file of the Macaca_fascicularis_5.0 genome, which contained both introns and exons. The aligned sequences were then sorted using sambamba (RRID:SCR_024328) (version 0.7). Finally, we obtained the UMI counts matrix of the cell to the gene. We removed cells that had unannotated genes, less than 3 detected genes, or mitochondrial expression exceeding 5%. Additionally, cells with less than 500 genes and those with the top 5% of the highest number of genes were filtered out.

To analyze the resulting matrix, we utilized the Seurat package (RRID:SCR_016341) (V 4.0.3) [5] for dimensionality reduction and clustering. We used monkeys as a batch and applied NormalizeData and FindVariableFeatures processes to two monkeys separately. We then selected 2000 feature genes using the SelectIntegrationFeatures equation, identified anchor points using the FindIntegrationAnchors equation, and integrated the data using the IntegrateData equation. To reduce dimensionality and cluster, we employed ScaleData, RunPCA, RunUMAP, FindNeighbors, and FindClusters equations. For identifying differentially expressed genes, we used either FindAllMarkers or FindMarkers equations.

After obtaining the differentially expressed genes (DEGs), we compared the Gene Ontology (GO) enrichment between different clusters using the compareCluster equation. Alternatively, we used the Metascape (RRID:SCR_016620) to calculate the GO enrichment.

Feature Selection and Module Recognition in AST Cell Types using Hotspot Analysis

To identify the gene signature modules of AST, we used the hotspot package (V 0.9.0) of Python

(3.8.12) [3] to perform Feature Selection and Module Recognition via Hotspot analysis on AST cell types. First, we identified the top 5000 variable features in AST and removed the mitochondrial genes. Next, we applied the hotspot.Hotspot equation to create a hotspot object and set the neighborhood size to 30 by using the create_knn_graph function. The autocorrelations of each gene were then computed using the compute_autocorrelations function to identify the genes with the most informative variation. Next, we calculated the pairwise local correlation of features through hs_results.loc and compute_local_correlations functions. Finally, we generated the gene module clustering results by using the create_modules function.

snATAC-seq data processing and quality control

We referred to previous literature and utilized the open-source PISA software workflow to process the snATAC-seq data of DNBelab C4 [84, 85]. We aligned the retained reads to the Macaca fascicularis genome, filtered out reads that aligned to mitochondrial or genomic scaffolds (including those starting with chrAQ, chrU, chrK, and those containing random segments), as well as reads with alignment quality less than 10 and PCR duplicates. The fragments obtained from each library in the aforementioned steps are used for downstream analysis.

snATAC-seq data clustering and analysis

First, we performed initial clustering of the raw data using the ArchR package in R software (version 1.0.2) [17], retaining cells with more than 1000 fragments and TSS enrichment higher than 6. We calculated the doublet score for each cell using the addDoubletScores function, with the parameter filterRatio = 2 used to filter out cells that may be doublets. Before the initial clustering, we created

a tiled matrix of 500bp bins using the genome. Then, we used LSI to reduce the top 25,000 features of the tiled matrix to 30 dimensions, with two iterations. To ensure the accuracy of each iteration, we inputted all cells. Subsequently, batch correction was performed using the Harmony function with donors and brain regions, followed by identification of clusters using Seurat's SNN graph clustering method at the default resolution of 0.8. Identified clusters were then used to call peaks with macs2, and different cell types' DA peaks were calculated using the getMarkerFeatures function with parameters "FDR <= 0.01 & Log2FC >= 2". For the re-clustering of the neuronal system and non-neuronal cells, we used the peak matrix generated by ArchR as input to Signac (version 1.1.0) for data normalization, feature selection, and dimensionality reduction analysis using the default workflow[86]. The data was then integrated by sample source and batch corrected using the RunHarmony function in the R package Harmony (version 0.1.0) [87]. Finally, a two-dimensional clustering result was obtained through UMAP analysis using 30 dimensions.

Co-clustering of snRNA-seq and snATAC-seq data.

First, we extracted the cell gene score and peak matrix from the ArchR object of scATAC-seq. Then, we performed normalization, feature selection, and dimensionality reduction analysis on the scATAC-seq data using the Signac standard pipeline in R. For scRNA-seq data, we performed corresponding dimensionality reduction analysis using the standard pipeline in Seurat. Subsequently, we used the FindTransferAnchors function to calculate anchors between different omics cells using the gene score matrix from scATAC-seq and the gene expression matrix from scRNA-seq, with the top 2000 VariableFeatures (calculated from scRNA-seq data) in common. To improve the accuracy of anchors, we set k.anchor to 20. Subsequently, these anchors were used to assign a predicted ID

to each cell in scATAC-seq, and scATAC cells with a score greater than 0.6 were retained and given a predicted gene expression matrix. The data from the two datasets were then co-embedded into a low-dimensional space with 30 dimensions using standard UMAP (RRID:SCR_018217) analysis.

Linking gene expression from snRNA-seq to cis-regulatory elements from snATAC-seq

To improve the accuracy of the peak-to-gene linkages, we performed supervised integration by inputting scRNA-seq data by cell type into the ArchR object of scATAC-seq. Subsequently, we used the addPeak2GeneLinks function in ArchR to establish links between genes and peaks, and then retained the links with a correlation greater than 0.45 and an FDR less than 0.01.

TF binding motif activity calculation

We calculated the enrichment of TF binding motifs by inputting peak activity matrices containing different cell populations into the R package chromVAR (version 1.18.0)[88]. First, we calculated the GC bias using the BSgenome.Mfascicularis.NCBI.5.0 genome, and then we downloaded the human TF binding motif database (human_pwms_v2) from the chromVARmotifs R package. We used the matchMotifs function to select peaks that retained TF binding motifs. Next, we calculated the activity of TF binding motifs for each cell using the computeDeviations function and extracted the bias-corrected deviations matrix for downstream analysis. We retained TFs with a variability greater than 1.5 by computing the variability for each TF. We then used the cell type-specific TF binding motif enrichment matrix to perform Wilcoxon tests to detect TFs that were enriched in different cell types.

Constructing a TF regulatory network

If a cis-regulatory elements (cRE) or differential accessible cis-regulatory elements (DA-cRE) associated with a gene contains a binding motif for a certain TF, it is defined that this TF may regulate the gene. If the cRE or DA-cRE falls within the promoter, intron, exon, or distal region of the gene, different modes of regulation are named accordingly. Finally, by inputting the TF-gene associations of different cell types into Cytoscape, a regulatory network is constructed. The color of the edges indicates the different modes of regulation of the transcription factors, while the color of the nodes represents the transcription factors and differentially expressed genes in different cell types.

Predicting the transcription factor enrichment of DA cRE

Input the DA-cRE sets of different cell types into Homer (version 4.11) to calculate the activity of TF binding motifs[89]. Retain the results in the knownResults output file and use the Benjamini-Hochberg test to assess the enrichment levels of different TFs.

Evaluating GWAS enrichment using cell type-specific open regions

We used LDSC to analyze the genetic variation in differentially accessible regions of different cell types and its correlation with GWAS results. First, we retained DA cRE with FDR <= 0.1 and Log2FC >= 0.5, and filtered out cell types with less than 100 DA cRE. We then used the liftover software to convert genome data to human hg19 genome data. To prepare for cluster-specific peak analysis for LDSC, we used the make_annotation.py script, and then calculated LD scores of SNPs in differentially accessible peaks using the ldsc.py script with 1000 Genomes phase 3 data. Then,

we downloaded GWAS summary statistics data from the UK Biobank database and publications. Finally, we input HapMap3 SNPs and corresponding 1000G_EUR_Phase3_baseline data and used the standard process to calculate cell type-specific genetic variation.

Data Availability

The raw FASTQ data for both snRNA-seq and snATAC-seq generated in this study are available in the EBI ENA under bioproject PRJNA970949 and the CNGB Nucleotide Sequence Archive using the accession code CNP0003589. All additional supporting data are available in the *GigaScience* database, GigaDB [90].

Abbreviations

AD: Alzheimer's disease; AMY: amygdala; ASD: autism spectrum disorder; AST: astrocyte; Cd: caudate; cREs: cis-regulatory elements; DA cREs: differential accessible cRE; DEG: differentially expressed gene; ENDO: endothelial cell; EX: excitatory neuron; GP: globus pallidum; GO: Gene Ontology; GWAS: genome-wide association studies; HD: Huntington's disease; IN: inhibitory neuron; LDSC: linkage-disequilibrium score regression; MDD: major depressive disorder; MIC: microglia; MSN: medium spiny neurons; NHP: Non-human primate; OPC: oligodendrocyte progenitor cell; OLIG: oligodendrocyte; Pu: putamen; SCZ: schizophrenia; SLM: smart local moving; SN: substant

Competing Interests

The authors declare that they have no competing interests.

Funding

This work was supported by National Key Research and Development Program (No.2022YEF0203200), National Science and Technology Innovation 2030 Major Program (Grant No. STI2030-2021ZD0200100), National Key Research and Development Program (2018YFA0801400and 2021YFA0805100).

Authors' contributions

Y.L., L.L and P.S conceived and designed the study. Y.S., and Z.L wrote the manuscript and Y.L., L.D and J.Y contributed to the discussion and revision of the manuscript. M.C contributed to sample collection. L.W., Z.Z., J.H and C.C participated in guiding and providing suggestions for the study. Y.H., Z.Z., S.J., F.H and C.W Provided technical support. All authors read and approved the final manuscript.

Acknowledgements

We sincerely thank China National Gene Bank for providing technical support.

Main figures

Figure. 1 single-cell transcriptomic and epigenomic characterization of cellular diversity in the basal ganglia

A, B, UMAP plot showing the clustering of 101,431nuclei from snRNA-seq (A) and 170,608 nuclei from snATAC-seq (B). Each point represents a nucleus and the colors indicate different cell types. There are 52 cell subtypes in snRNA-seq and 50 cell subtypes in snATAC-seq

C, Expression of cell type marker genes for the main cell types is shown. The color represents the

expression level and the size represents the percentage expression value

D, Integrative Genomics Viewer (IGV) plot showing the read density of cell type-specific marker genes on snATAC-seq cell types in C

E, UMAP plot showing the low-dimensional co-embedding clustering of snRNA-seq and snATAC-seq. The main cell types are marked with different colors

F, Integrated UMAP in F and separated by data type

G, Heatmap showing the chromatin accessibility and gene expression of 109,506 significantly linked cRE-gene pairs. It is represented by a one-to-one heatmap with cREs activity on the left and its linked gene expression value on the right. cREs and genes can link to each other mutually. Hence, each cRE and gene may appear repeatedly in the corresponding rows of the heatmap. The cRE-gene link is clustered by k-means (k=25)

H, Heatmap showing the motif activity of TFs in different cell types in snATAC-seq

Figure. 2 Transcriptional regulation heterogeneity of neuron-type-specific and region-specific genes in the basal ganglia.

A, UMAP plot displays the sub-classification of basal ganglia neurons from snRNA-seq, with colors representing different subtypes of cells.

B, UMAP plot showing the expression of classical marker genes for neurons in snRNA-seq data.

C, UMAP plot displays the sub-classification of basal ganglia neurons from snATAC-seq, with colors representing different subtypes of cells.

D, Dot plot illustrating the expression (left) and activity (right) patterns of differentially expressed genes (DEGs) in neuronal subtypes, and novel markers identified here

E, Volcano plot representing the DEGs between IN_SST subtypes from snRNA-seq (left), and the differential accessible chromatin (DA) cREs ($|\text{Log2 FC}| > 1.5$ and $\text{FDR} < 0.01$) between IN_SST

subtypes from snATAC-seq (right), where each point represents a gene or a DA cREs, respectively. The points marked with corresponding subtypes indicate the DEGs or DA cREs linked to them

F, Violin plots showing MSN subtype-specific marker gene expression.

G, The number of significantly correlated peaks (FDR < 0.01) for each MSN subtype DEG (FDR < 0.01).

H, The gene score of DORCs in subtypes of MSN, with red indicating downregulated genes in patients with Huntington's disease.

I, The gene expression of *ADORA2A* (upper panel) and *ADORA2A* gene score (lower panel) colored in UMAP plot.

J, The upper panel shows the UMAP plot of snATAC-seq MSN recluster colored by NPAS2 motif variability, while the lower panel displays Tn5 bias-subtracted TF footprinting for NPAS2 by snATAC-seq MSN subcluster (left) and NPAS2 target gene expression in MSN subcluster (right).

Figure.3 Substantia nigra-specific astrocyte subpopulations

A, UMAP plot representing subtypes of ASTs from snRNA-seq, where colors represent different cell types (left), and their regional distribution (right)

B, The violin plot shows the differential gene expression patterns of the AST subtypes in snRNA-seq

C, 2404 significantly auto-correlated genes (FDR <0.05) were clustered into five modules based on pairwise correlation

D, Violin plot showing the enrichment of genes related to Dopaminergic Neurogenesis and Alzheimer's Disease in the AST4 subtype

E, The violin plot shows the expression patterns of selected transcription factors with specific expression in AST4 across AST subtypes

F, Heatmap displaying the correlation between AST subtypes from snRNA-seq and snATAC-seq, revealing a correlation between AST4 from snRNA-seq and AST_c from snATAC-seq

G, Heatmap showing the enrichment of transcription factor motifs in AST cell subtypes from snATAC-seq

H, Visualization of the predicted peak-gene pairs containing transcription factor motif binding sites for RFX4 and PBX3 in the locus of the Alzheimer's Disease-related gene *SLC39A12*

Figure.4 Cross-species comparison of cell type similarity and heterogeneity in the substantia nigra.

A, Co-embedding of human, monkey, and mouse substantia nigra data, with colors representing different species

B, Venn diagrams showing DEGs of SN celltypes shared across species

C, Heatmap illustrating the expression patterns of both cross-species conserved and species-specific DEGs

D, Gene activity values of conserved DEGs across species (left panel) and their corresponding DA cREs activity values

E, Transcription factors enriched in the DA cREs linked to conserved differentially expressed genes

F, Violin plot representing AST-specific enrichment of differentially expressed genes in the SN related to functional pathways such as Diseases of signal transduction by growth factor receptors and second messengers, Alzheimer disease, and Enzyme-linked receptor protein signaling pathway

G, Heatmap showing AST-specific activity of TF motifs in SN (left) and Violin plot displaying the specific expression of these TFs in AST (right)

H, Genome track visualization of the Diseases of signal transduction by growth factor receptors and second messengers-related gene *FGFR1* locus. Inferred peak-gene links for distal or Intronic regulatory elements which containing transcription factor motif binding sites for NFIA and RFX4

Figure.5 Cell-type specific regulatory landscape of GWAS loci in the basal ganglia

A, Heatmap showing LDSC enrichment of GWAS traits and disorders in snATAC-seq clusters. BD, bipolar disorder; MDD, major depressive disorder; SCZ, schizophrenia; BMI, body mass index; ADHD, attention deficit hyperactivity disorder; autism spectrum disorder; T2D, type 2 diabetes; PD, Parkinson’s disease; ALS, amyotrophic lateral sclerosis; AD, Alzheimer’s disease

B, rs12293670 GWAS locus and cis-regulatory architecture in snATAC-seq cell types

C, Violin plot showing the expression levels of the *NRGN* gene across different subtypes in snRNA-seq

Supplementary Figures

Supplementary Figure. 1 Quality assessment of snRNA-seq and snATAC-seq data

A, Bar graph representing the proportion of subtypes in snRNA-seq, with colors consistent with Figure 1A

B, Box plot of plot of unique molecular identifiers counts (UMI) (up), detected gene number (middle) and Proportion of mt (Mitochondrial) genes of snRNA-seq cells in each snRNA-seq celltype.

C, Bar graph representing the proportion of subtypes in snATAC-seq, with colors consistent with Figure 1B

D, Box plot showing the TSS enrichment, which is calculated as the average abundance of read

counts in the 50 bp upstream and downstream of the TSS, divided by the average accessibility of the TSS flanking positions (+/- 1900 – 2000 bp) (top). The TSS ± 2 kb ratio, which represents the proportion of peaks located within 2 kb from the gene TSS site, is shown in the middle panel. The fragment counts (bottom) of snATAC-seq cells in each snATAC-seq cluster are also displayed

E, UMAP plot showing the comparison of snRNA-seq before and after batch correction (top), with the color indicating the donor source. The snATAC-seq plot (bottom) also shows the comparison before and after batch correction with the donor source consistent with snRNA-seq. The amygdala region is only present in monkey2 in both datasets, while monkey1 is the exclusive possessor of the SN region in snATAC-seq

F, UMAP plot displaying the region of origin for snRNA-seq (top) and snATAC-seq (bottom), with colors indicating different regions

G, Heatmaps showing differential open chromatin activity of 429,541 snATAC-seq DA cREs identified by bias-matched differential testing (FDR<0.01 and log 2 FC >= 2) across 47 cell types

Supplementary Figure. 2 Heterogeneity analysis of neuronal subtypes.

A, UMAP plot displays the sub-classification of basal ganglia neurons from snRNA-seq (left) and snATAC-seq (right), with colors represent different region of cells.

B, Gene ontology terms enriched among genes with distinct expression pattern between IN_SST-1 and IN_SST-2

C, Violin plots representing the motif enrichment and targeted gene scores of selected TFs that were significantly upregulated in IN_SST-1 and IN_SST-2

D, TF regulatory networks showing the predicted target DEGs for transcription factors TFAP4,

NHLH2, ASCL2, TCF21, and ZNF238 in IN_SST-1 subtype inhibitory neurons and the predicted target DEGs for transcription factors NR1D1, POU2F3, POU2F1, POU5F1, and SNAI2 in IN_SST-2 subtype inhibitory neurons

E, Heatmap showing the top ten most enriched genes in each PVALB type

F, Heatmap of subtype-specific activity patterns of DA cREs in IN_PVALB neurons from snATAC-seq data, marked with DEGs linked to corresponding snRNA-seq cell types

G, Dot plot showing enriched pathways for the IN_PVALB subtype DEGs

Supplementary Figure. 3 Comparative analysis of MSN neurons across species

A, Top: MSN re-clustering UMAP plot of snRNA-seq (left) and snATAC-seq (right). Middle: KCNIP1 gene expression (left) and gene activity (right). Bottom: STXBP6 gene expression (left) and gene activity (right)

B, TF motif scores for each MSN subtype cluster

C, Heatmap displaying the correlation between macaque and mouse MSNs, with macaque DRD2-MSN4 showing the strongest correlation with mouse eMSNs

D, Bar graph showing the number of DEGs in macaque and mouse MSNs, with macaque D1_MSN including D1-MSN_Matrix, D1-MSN_Striosome, while D2_MSN comprises D2-MSN_Matrix, D2-MSN_Striosome. mouse eMSN refers to D1_D2_hybrid

E, Volcano plot displaying the DEGs in macaque and mouse D1_D2_hybrid

F, Gene ontology terms enriched among DEGs specific to macaque and mouse D1_D2_hybrid

Supplementary Figure. 4 Heterogeneity of non-neuronal cells

A, DEGs (left) and regional distribution (right) of non-neuronal cell subtypes including OPC, MIC, OLIG, and ENDO

B, Visualization of five distinct gene modules in AST subtype on UMAP (left) and highlights of AST subtype

C, Histogram depicting regional distribution of AST subtypes in snRNA-seq (left) and snATAC-seq (right)

D, Specific expression of Huntington's disease-related genes in AST5 cluster

Supplementary Figure. 5 Cross-species conservation and primate specificity of SN cell types

A, UMAP plot from Figure 4A, grouped by species and colored by within-species clusters

B, Proportion of nuclei that overlap between macaque (rows, color by Fig. 4C) and human or mouse clusters in the integrated space

C, Violin plot showing enrichment of DEGs specific to DaNs neurons in the primate brain for functional pathways related to brain development, actin filament-based processes, enzyme-linked receptor protein signaling pathway, and cardiac muscle contraction

D, Transcription factor regulatory network diagram depicting NFIA, ARID2, and RFX4, highly active transcription factors in SN AST cell types, and their predicted target DEGs. The colors represent DEG enrichment in different pathways

References

1. Lanciego JL, Luquin N and Obeso JA. Functional neuroanatomy of the basal ganglia. Cold Spring Harb Perspect Med. 2012;2 12:a009621. doi:10.1101/cshperspect.a009621.
2. DeLong M and Wichmann T. Update on models of basal ganglia function and dysfunction.

Parkinsonism Relat Disord. 2009;15 Suppl 3 0 3:S237-40. doi:10.1016/S1353-8020(09)70822-3.

3. Wu YE, Pan L, Zuo Y, Li X and Hong W. Detecting Activated Cell Populations Using Single-Cell RNA-Seq. Neuron. 2017;96 2:313-29 e6. doi:10.1016/j.neuron.2017.09.026.
4. Zhang L, Cheng Y, Wu S, Lu Y, Xue Z, Chen X, et al. Molecular taxonomy of the primate amygdala via single-nucleus RNA sequencing analysis. Sci Bull (Beijing). 2021;66 14:1379-83. doi:10.1016/j.scib.2021.01.017.
5. Mathys H, Davila-Velderrain J, Peng Z, Gao F, Mohammadi S, Young JZ, et al. Single-cell transcriptomic analysis of Alzheimer's disease. Nature. 2019;570 7761:332-7. doi:10.1038/s41586-019-1195-2.
6. Nagy C, Maitra M, Tanti A, Suderman M, Theroux JF, Davoli MA, et al. Single-nucleus transcriptomics of the prefrontal cortex in major depressive disorder implicates oligodendrocyte precursor cells and excitatory neurons. Nat Neurosci. 2020;23 6:771-81. doi:10.1038/s41593-020-0621-y.
7. Schirmer L, Velmeshev D, Holmqvist S, Kaufmann M, Werneburg S, Jung D, et al. Neuronal vulnerability and multilineage diversity in multiple sclerosis. Nature. 2019;573 7772:75-82. doi:10.1038/s41586-019-1404-z.
8. Velmeshev D, Schirmer L, Jung D, Haeussler M, Perez Y, Mayer S, et al. Single-cell genomics identifies cell type-specific molecular changes in autism. Science. 2019;364 6441:685-9. doi:10.1126/science.aav8130.
9. Skene NG, Bryois J, Bakken TE, Breen G, Crowley JJ, Gaspar HA, et al. Genetic identification of brain cell types underlying schizophrenia. Nat Genet. 2018;50 6:825-33. doi:10.1038/s41588-018-0129-5.
10. Agarwal D, Sandor C, Volpato V, Caffrey TM, Monzon-Sandoval J, Bowden R, et al. A single-cell atlas of the human substantia nigra reveals cell-specific pathways associated with neurological disorders. Nat Commun. 2020;11 1:4183. doi:10.1038/s41467-020-17876-0.
11. Gokce O, Stanley GM, Treutlein B, Neff NF, Camp JG, Malenka RC, et al. Cellular Taxonomy of the Mouse Striatum as Revealed by Single-Cell RNA-Seq. Cell Rep. 2016;16 4:1126-37. doi:10.1016/j.celrep.2016.06.059.
12. Lake BB, Chen S, Sos BC, Fan J, Kaeser GE, Yung YC, et al. Integrative single-cell analysis of transcriptional and epigenetic states in the human adult brain. Nat Biotechnol. 2018;36 1:70-80. doi:10.1038/nbt.4038.
13. Corces MR, Buenrostro JD, Wu B, Greenside PG, Chan SM, Koenig JL, et al. Lineage-specific and single-cell chromatin accessibility charts human hematopoiesis and leukemia evolution. Nat Genet. 2016;48 10:1193-203. doi:10.1038/ng.3646.
14. Saunders A, Macosko EZ, Wysoker A, Goldman M, Krienen FM, de Rivera H, et al. Molecular Diversity and Specializations among the Cells of the Adult Mouse Brain. Cell. 2018;174 4:1015-30 e16. doi:10.1016/j.cell.2018.07.028.
15. Krienen FM, Goldman M, Zhang Q, R CHDR, Florio M, Machold R, et al. Innovations present in the primate interneuron repertoire. Nature. 2020;586 7828:262-9. doi:10.1038/s41586-020-2781-z.
16. He J, Kleyman M, Chen J, Alikaya A, Rothenhoefer KM, Ozturk BE, et al. Transcriptional and anatomical diversity of medium spiny neurons in the primate striatum. Curr Biol.

2021;31 24:5473-86 e6. doi:10.1016/j.cub.2021.10.015.
17. Granja JM, Corces MR, Pierce SE, Bagdatli ST, Choudhry H, Chang HY, et al. ArchR is a scalable software package for integrative single-cell chromatin accessibility analysis. Nat Genet. 2021;53 3:403-11. doi:10.1038/s41588-021-00790-6.
18. Bayam E, Sahin GS, Guzelsoy G, Guner G, Kabakcioglu A and Ince-Dunn G. Genome-wide target analysis of NEUROD2 provides new insights into regulation of cortical projection neuron migration and differentiation. BMC Genomics. 2015;16:681. doi:10.1186/s12864-015-1882-9.
19. Lin CH, Hansen S, Wang Z, Storm DR, Tapscott SJ and Olson JM. The dosage of the neuroD2 transcription factor regulates amygdala development and emotional learning. Proc Natl Acad Sci U S A. 2005;102 41:14877-82. doi:10.1073/pnas.0506785102.
20. Tam RW and Keung AJ. Human Pluripotent Stem Cell-Derived Medium Spiny Neuron-like Cells Exhibit Gene Desensitization. Cells. 2022;11 9 doi:10.3390/cells11091411.
21. Muzio L, Di Benedetto B, Stoykova A, Boncinelli E, Gruss P and Mallamaci A. Conversion of cerebral cortex into basal ganglia in Emx2(-/-) Pax6(Sey/Sey) double-mutant mice. Nat Neurosci. 2002;5 8:737-45. doi:10.1038/nn892.
22. Liu Z, Wang X, Jiang K, Ji X, Zhang YA and Chen Z. TNFalpha-induced Up-regulation of Ascl2 Affects the Differentiation and Proliferation of Neural Stem Cells. Aging Dis. 2019;10 6:1207-20. doi:10.14336/AD.2018.1028.
23. Jiang H, Du M, Li Y, Zhou T, Lei J, Liang H, et al. ID proteins promote the survival and primed-to-naive transition of human embryonic stem cells through TCF3-mediated transcription. Cell Death Dis. 2022;13 6:549. doi:10.1038/s41419-022-04958-8.
24. Chen J, Leong SY and Schachner M. Differential expression of cell fate determinants in neurons and glial cells of adult mouse spinal cord after compression injury. Eur J Neurosci. 2005;22 8:1895-906. doi:10.1111/j.1460-9568.2005.04348.x.
25. Chen T, Zhou L, Yuan Y, Fang Y, Guo Y, Huang H, et al. Characterization of Bbx, a member of a novel subfamily of the HMG-box superfamily together with Cic. Dev Genes Evol. 2014;224 4-6:261-8. doi:10.1007/s00427-014-0476-x.
26. Stolt CC, Lommes P, Sock E, Chaboissier MC, Schedl A and Wegner M. The Sox9 transcription factor determines glial fate choice in the developing spinal cord. Genes Dev. 2003;17 13:1677-89. doi:10.1101/gad.259003.
27. Saddala MS, Yang X, Tang S and Huang H. Transcriptome-wide analysis reveals core sets of transcriptional regulators of sensome and inflammation genes in retinal microglia. Genomics. 2021;113 5:3058-71. doi:10.1016/j.ygeno.2021.07.001.
28. Reyahi A, Nik AM, Ghiami M, Gritli-Linde A, Ponten F, Johansson BR, et al. Foxf2 Is Required for Brain Pericyte Differentiation and Development and Maintenance of the Blood-Brain Barrier. Dev Cell. 2015;34 1:19-32. doi:10.1016/j.devcel.2015.05.008.
29. Kadkhodaei B, Ito T, Joodmardi E, Mattsson B, Rouillard C, Carta M, et al. Nurr1 is required for maintenance of maturing and adult midbrain dopamine neurons. J Neurosci. 2009;29 50:15923-32. doi:10.1523/JNEUROSCI.3910-09.2009.
30. Suss ST, Olbricht LM, Herlitze S and Spoida K. Constitutive 5-HT2C receptor knock-out facilitates fear extinction through altered activity of a dorsal raphe-bed nucleus of the stria terminalis pathway. Transl Psychiatry. 2022;12 1:487. doi:10.1038/s41398-022-02252-x.
31. Burglen L, Van Hoeymissen E, Qebibo L, Barth M, Belnap N, Boschann F, et al. Gain-of-

function variants in the ion channel gene TRPM3 underlie a spectrum of neurodevelopmental disorders. Elife. 2023;12 doi:10.7554/eLife.81032.

32. Chacon PJ, del Marco A, Arevalo A, Dominguez-Gimenez P, Garcia-Segura LM and Rodriguez-Tebar A. Cerebellin 4, a synaptic protein, enhances inhibitory activity and resistance of neurons to amyloid-beta toxicity. Neurobiol Aging. 2015;36 2:1057-71. doi:10.1016/j.neurobiolaging.2014.11.006.

33. Li D, Zhao W, Zhang X, Lv H, Li C and Sun L. NEFM DNA methylation correlates with immune infiltration and survival in breast cancer. Clin Epigenetics. 2021;13 1:112. doi:10.1186/s13148-021-01096-4.

34. Maalmi H, Strom A, Petrera A, Hauck SM, Strassburger K, Kuss O, et al. Serum neurofilament light chain: a novel biomarker for early diabetic sensorimotor polyneuropathy. Diabetologia. 2023;66 3:579-89. doi:10.1007/s00125-022-05846-8.

35. Moon SW, Son HJ, Chae J, Yoo NJ, An CH and Lee SH. Expression and Mutation Alterations of ZMYM4 Gene in Gastric and Colonic Cancers. Appl Immunohistochem Mol Morphol. 2021;29 8:570-5. doi:10.1097/PAI.0000000000000939.

36. Lei X, Liu L, Terrillion CE, Karuppagounder SS, Cisternas P, Lay M, et al. FAM19A1, a brain-enriched and metabolically responsive neurokine, regulates food intake patterns and mouse behaviors. FASEB J. 2019;33 12:14734-47. doi:10.1096/fj.201901232RR.

37. Urban-Ciecko J and Barth AL. Somatostatin-expressing neurons in cortical networks. Nat Rev Neurosci. 2016;17 7:401-9. doi:10.1038/nrn.2016.53.

38. Yager LM, Garcia AF, Wunsch AM and Ferguson SM. The ins and outs of the striatum: role in drug addiction. Neuroscience. 2015;301:529-41. doi:10.1016/j.neuroscience.2015.06.033.

39. Gerfen CR and Surmeier DJ. Modulation of striatal projection systems by dopamine. Annu Rev Neurosci. 2011;34:441-66. doi:10.1146/annurev-neuro-061010-113641.

40. Ma S, Zhang B, LaFave LM, Earl AS, Chiang Z, Hu Y, et al. Chromatin Potential Identified by Shared Single-Cell Profiling of RNA and Chromatin. Cell. 2020;183 4:1103-16 e20. doi:10.1016/j.cell.2020.09.056.

41. Kuhn A, Goldstein DR, Hodges A, Strand AD, Sengstag T, Kooperberg C, et al. Mutant huntingtin's effects on striatal gene expression in mice recapitulate changes observed in human Huntington's disease brain and do not differ with mutant huntingtin length or wild-type huntingtin dosage. Hum Mol Genet. 2007;16 15:1845-61. doi:10.1093/hmg/ddm133.

42. Parekh PK, Logan RW, Ketchesin KD, Becker-Krail D, Shelton MA, Hildebrand MA, et al. Cell-Type-Specific Regulation of Nucleus Accumbens Synaptic Plasticity and Cocaine Reward Sensitivity by the Circadian Protein, NPAS2. J Neurosci. 2019;39 24:4657-67. doi:10.1523/JNEUROSCI.2233-18.2019.

43. Smeets WJ, Marin O and Gonzalez A. Evolution of the basal ganglia: new perspectives through a comparative approach. J Anat. 2000;196 (Pt 4) Pt 4:501-17. doi:10.1046/j.1469-7580.2000.19640501.x.

44. Linnerbauer M, Wheeler MA and Quintana FJ. Astrocyte Crosstalk in CNS Inflammation. Neuron. 2020;108 4:608-22. doi:10.1016/j.neuron.2020.08.012.

45. Martin-Fernandez M, Jamison S, Robin LM, Zhao Z, Martin ED, Aguilar J, et al. Synapse-specific astrocyte gating of amygdala-related behavior. Nat Neurosci. 2017;20 11:1540-8. doi:10.1038/nn.4649.

46. Whalley K. Reprogramming astrocytes for repair. Nat Rev Neurosci. 2019;20 11:647. doi:10.1038/s41583-019-0227-0.

47. Yun SP, Kam TI, Panicker N, Kim S, Oh Y, Park JS, et al. Block of A1 astrocyte conversion by microglia is neuroprotective in models of Parkinson's disease. Nat Med. 2018;24 7:931-8. doi:10.1038/s41591-018-0051-5.

48. Khakh BS, Beaumont V, Cachope R, Munoz-Sanjuan I, Goldman SA and Grantyn R. Unravelling and Exploiting Astrocyte Dysfunction in Huntington's Disease. Trends Neurosci. 2017;40 7:422-37. doi:10.1016/j.tins.2017.05.002.

49. Habib N, McCabe C, Medina S, Varshavsky M, Kitsberg D, Dvir-Szternfeld R, et al. Disease-associated astrocytes in Alzheimer's disease and aging. Nat Neurosci. 2020;23 6:701-6. doi:10.1038/s41593-020-0624-8.

50. Colombo E and Farina C. Astrocytes: Key Regulators of Neuroinflammation. Trends Immunol. 2016;37 9:608-20. doi:10.1016/j.it.2016.06.006.

51. Diaz-Castro B, Bernstein AM, Coppola G, Sofroniew MV and Khakh BS. Molecular and functional properties of cortical astrocytes during peripherally induced neuroinflammation. Cell Rep. 2021;36 6:109508. doi:10.1016/j.celrep.2021.109508.

52. Braunstein KE, Eschbach J, Rona-Voros K, Soylu R, Mikrouli E, Larmet Y, et al. A point mutation in the dynein heavy chain gene leads to striatal atrophy and compromises neurite outgrowth of striatal neurons. Hum Mol Genet. 2010;19 22:4385-98. doi:10.1093/hmg/ddq361.

53. Carmichael K, Evans RC, Lopez E, Sun L, Kumar M, Ding J, et al. Function and Regulation of ALDH1A1-Positive Nigrostriatal Dopaminergic Neurons in Motor Control and Parkinson's Disease. Front Neural Circuits. 2021;15:644776. doi:10.3389/fncir.2021.644776.

54. Yan Y, Tulasne D, Browaeys E, Cailliau K, Khayath N, Pierce RJ, et al. Molecular cloning and characterisation of SmSLK, a novel Ste20-like kinase in Schistosoma mansoni. Int J Parasitol. 2007;37 14:1539-50. doi:10.1016/j.ijpara.2007.06.001.

55. Jayapalan S, Subramanian D and Natarajan J. Computational identification and analysis of neurodegenerative disease associated protein kinases in hominid genomes. Genes Dis. 2016;3 3:228-37. doi:10.1016/j.gendis.2016.04.004.

56. Zhang D, Stumpo DJ, Graves JP, DeGraff LM, Grissom SF, Collins JB, et al. Identification of potential target genes for RFX4_v3, a transcription factor critical for brain development. J Neurochem. 2006;98 3:860-75. doi:10.1111/j.1471-4159.2006.03930.x.

57. Davis DN, Strong MD, Chambers E, Hart MD, Bettaieb A, Clarke SL, et al. A role for zinc transporter gene SLC39A12 in the nervous system and beyond. Gene. 2021;799:145824. doi:10.1016/j.gene.2021.145824.

58. Sonne J, Reddy V and Beato MR. Neuroanatomy, Substantia Nigra. StatPearls. Treasure Island (FL); 2023.

59. Poewe W, Seppi K, Tanner CM, Halliday GM, Brundin P, Volkmann J, et al. Parkinson disease. Nat Rev Dis Primers. 2017;3:17013. doi:10.1038/nrdp.2017.13.

60. Matuzelski E, Bunt J, Harkins D, Lim JWC, Gronostajski RM, Richards LJ, et al. Transcriptional regulation of Nfix by NFIB drives astrocytic maturation within the developing spinal cord. Dev Biol. 2017;432 2:286-97. doi:10.1016/j.ydbio.2017.10.019.

61. Bajenaru ML, Zhu Y, Hedrick NM, Donahoe J, Parada LF and Gutmann DH. Astrocyte-

specific inactivation of the neurofibromatosis 1 gene (NF1) is insufficient for astrocytoma formation. Mol Cell Biol. 2002;22 14:5100-13. doi:10.1128/MCB.22.14.5100-5113.2002.
62. Takada N, Kucenas S and Appel B. Sox10 is necessary for oligodendrocyte survival following axon wrapping. Glia. 2010;58 8:996-1006. doi:10.1002/glia.20981.
63. Connolly NP, Shetty AC, Stokum JA, Hoeschele I, Siegel MB, Miller CR, et al. Cross-species transcriptional analysis reveals conserved and host-specific neoplastic processes in mammalian glioma. Sci Rep. 2018;8 1:1180. doi:10.1038/s41598-018-19451-6.
64. Palomer E, Buechler J and Salinas PC. Wnt Signaling Deregulation in the Aging and Alzheimer's Brain. Front Cell Neurosci. 2019;13 doi:ARTN 227
10.3389/fncel.2019.00227.
65. Pires-daSilva A and Sommer RJ. The evolution of signalling pathways in animal development. Nat Rev Genet. 2003;4 1:39-49. doi:10.1038/nrg977.
66. Bunt J, Osinski JM, Lim JW, Vidovic D, Ye Y, Zalucki O, et al. Combined allelic dosage of Nfia and Nfib regulates cortical development. Brain Neurosci Adv. 2017;1:2398212817739433. doi:10.1177/2398212817739433.
67. Finucane HK, Reshef YA, Anttila V, Slowikowski K, Gusev A, Byrnes A, et al. Heritability enrichment of specifically expressed genes identifies disease-relevant tissues and cell types. Nat Genet. 2018;50 4:621-9. doi:10.1038/s41588-018-0081-4.
68. Guadagno A, Verlezza S, Long H, Wong TP and Walker CD. It Is All in the Right Amygdala: Increased Synaptic Plasticity and Perineuronal Nets in Male, But Not Female, Juvenile Rat Pups after Exposure to Early-Life Stress. J Neurosci. 2020;40 43:8276-91. doi:10.1523/JNEUROSCI.1029-20.2020.
69. Warner-Schmidt JL, Schmidt EF, Marshall JJ, Rubin AJ, Arango-Lievano M, Kaplitt MG, et al. Cholinergic interneurons in the nucleus accumbens regulate depression-like behavior. Proc Natl Acad Sci U S A. 2012;109 28:11360-5. doi:10.1073/pnas.1209293109.
70. Song YH, Yoon J and Lee SH. The role of neuropeptide somatostatin in the brain and its application in treating neurological disorders. Exp Mol Med. 2021;53 3:328-38. doi:10.1038/s12276-021-00580-4.
71. Pacifico R and Davis RL. Transcriptome sequencing implicates dorsal striatum-specific gene network, immune response and energy metabolism pathways in bipolar disorder. Mol Psychiatry. 2017;22 3:441-9. doi:10.1038/mp.2016.94.
72. Pardinas AF, Holmans P, Pocklington AJ, Escott-Price V, Ripke S, Carrera N, et al. Common schizophrenia alleles are enriched in mutation-intolerant genes and in regions under strong background selection. Nat Genet. 2018;50 3:381-9. doi:10.1038/s41588-018-0059-2.
73. Martin A, Calvigioni D, Tzortzi O, Fuzik J, Warnberg E and Meletis K. A Spatiomolecular Map of the Striatum. Cell Rep. 2019;29 13:4320-33 e5. doi:10.1016/j.celrep.2019.11.096.
74. Emborg ME. Nonhuman primate models of Parkinson's disease. ILAR J. 2007;48 4:339-55. doi:10.1093/ilar.48.4.339.
75. Khakh BS and Sofroniew MV. Diversity of astrocyte functions and phenotypes in neural circuits. Nat Neurosci. 2015;18 7:942-52. doi:10.1038/nn.4043.
76. Batiuk MY, Martirosyan A, Wahis J, de Vin F, Marneffe C, Kusserow C, et al. Identification of region-specific astrocyte subtypes at single cell resolution. Nat Commun. 2020;11 1:1220. doi:10.1038/s41467-019-14198-8.

77. Kuter K, Olech L and Glowacka U. Prolonged Dysfunction of Astrocytes and Activation of Microglia Accelerate Degeneration of Dopaminergic Neurons in the Rat Substantia Nigra and Block Compensation of Early Motor Dysfunction Induced by 6-OHDA. Mol Neurobiol. 2018;55 4:3049-66. doi:10.1007/s12035-017-0529-z.
78. Segura-Aguilar J, Paris I, Munoz P, Ferrari E, Zecca L and Zucca FA. Protective and toxic roles of dopamine in Parkinson's disease. J Neurochem. 2014;129 6:898-915. doi:10.1111/jnc.12686.
79. Tosches MA, Yamawaki TM, Naumann RK, Jacobi AA, Tushev G and Laurent G. Evolution of pallium, hippocampus, and cortical cell types revealed by single-cell transcriptomics in reptiles. Science. 2018;360 6391:881-8. doi:10.1126/science.aar4237.
80. Vallender EJ, Hotchkiss CE, Lewis AD, Rogers J, Stern JA, Peterson SM, et al. Nonhuman primate genetic models for the study of rare diseases. Orphanet J Rare Dis. 2023;18 1:20. doi:10.1186/s13023-023-02619-3.
81. Jhanwar S, Malkmus J, Stolte J, Romashkina O, Zuniga A and Zeller R. Conserved and species-specific chromatin remodeling and regulatory dynamics during mouse and chicken limb bud development. Nat Commun. 2021;12 1:5685. doi:10.1038/s41467-021-25935-3.
82. Zoupi L, Savvaki M, Kalemaki K, Kalafatakis I, Sidiropoulou K and Karagogeos D. The function of contactin-2/TAG-1 in oligodendrocytes in health and demyelinating pathology. Glia. 2018;66 3:576-91. doi:10.1002/glia.23266.
83. Trevino AE, Müller F, Andersen J, Sundaram L, Kathiria A, Shcherbina A, et al. Chromatin and gene-regulatory dynamics of the developing human cerebral cortex at single-cell resolution. Cell. 2021;184 19:5053-69.e23. doi:10.1016/j.cell.2021.07.039.
84. Lei Y, Cheng M, Li Z, Zhuang Z, Wu L, Sun Y, et al. Spatially resolved gene regulatory and disease-related vulnerability map of the adult Macaque cortex. Nat Commun. 2022;13 1:6747. doi:10.1038/s41467-022-34413-3.
85. Shi Q, Liu S, Kristiansen K and Liu L. The FASTQ+ format and PISA. Bioinformatics. 2022;38 19:4639-42. doi:10.1093/bioinformatics/btac562.
86. Stuart T, Srivastava A, Madad S, Lareau CA and Satija R. Single-cell chromatin state analysis with Signac. Nat Methods. 2021;18 11:1333-41. doi:10.1038/s41592-021-01282-5.
87. Korsunsky I, Millard N, Fan J, Slowikowski K, Zhang F, Wei K, et al. Fast, sensitive and accurate integration of single-cell data with Harmony. Nat Methods. 2019;16 12:1289-96. doi:10.1038/s41592-019-0619-0.
88. Schep AN, Wu B, Buenrostro JD and Greenleaf WJ. chromVAR: inferring transcription-factor-associated accessibility from single-cell epigenomic data. Nat Methods. 2017;14 10:975-8. doi:10.1038/nmeth.4401.
89. Heinz S, Benner C, Spann N, Bertolino E, Lin YC, Laslo P, et al. Simple combinations of lineage-determining transcription factors prime cis-regulatory elements required for macrophage and B cell identities. Mol Cell. 2010;38 4:576-89. doi:10.1016/j.molcel.2010.05.004.
90. Li Z, Sun Y, Ding L, Yang J, Huang J, Cheng M, et al. Supporting data for "Deciphering the distinct transcriptomic and gene regulatory map in adult macaque basal ganglia cells" GigaScience Database. 2023. http://dx.doi.org/10.5524/102441.

Figure 1

Click here to access/download;Figure;Fig1.pdf

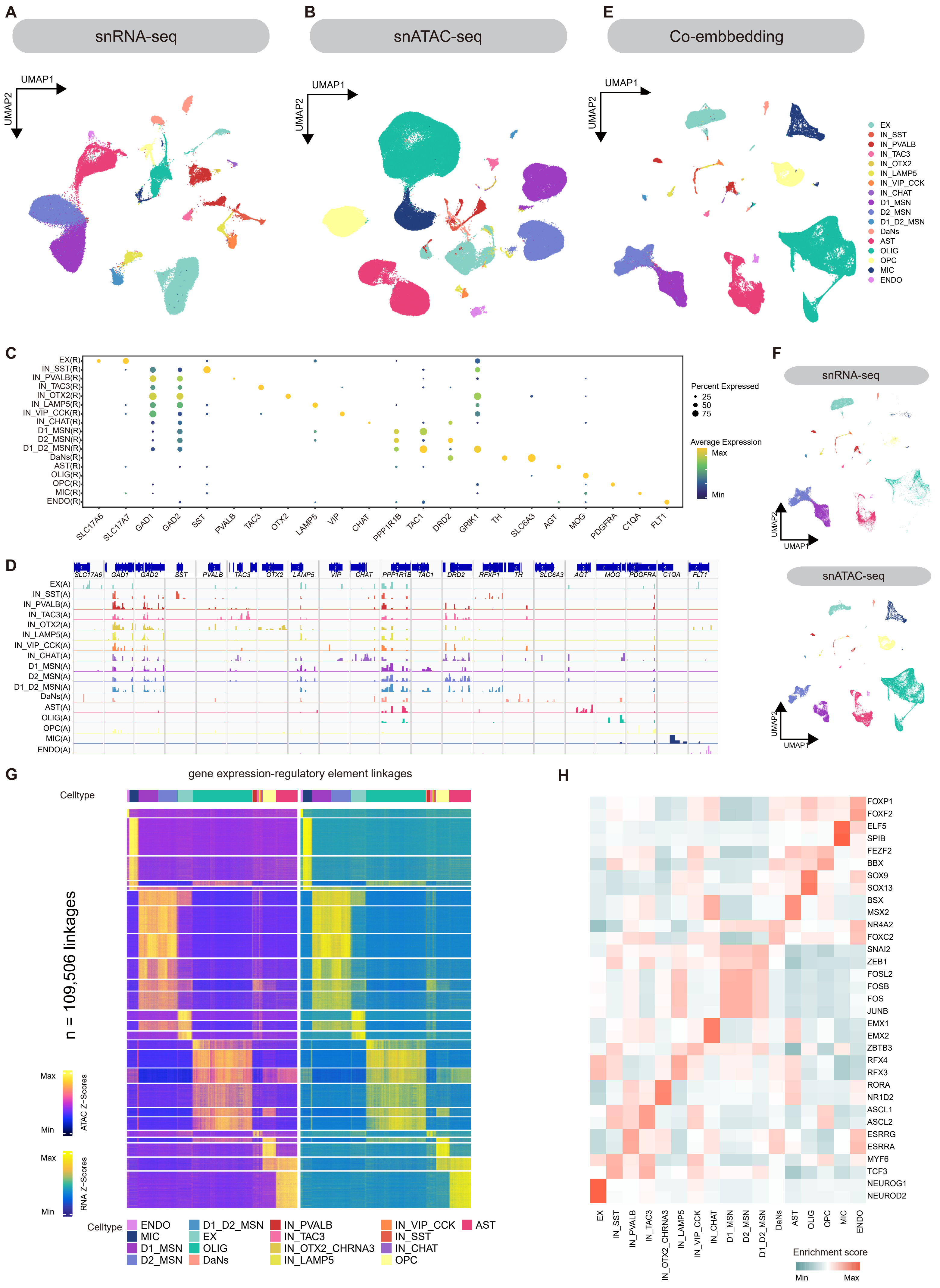

Figure 2

Click here to access/download;Figure;Fig2.pdf

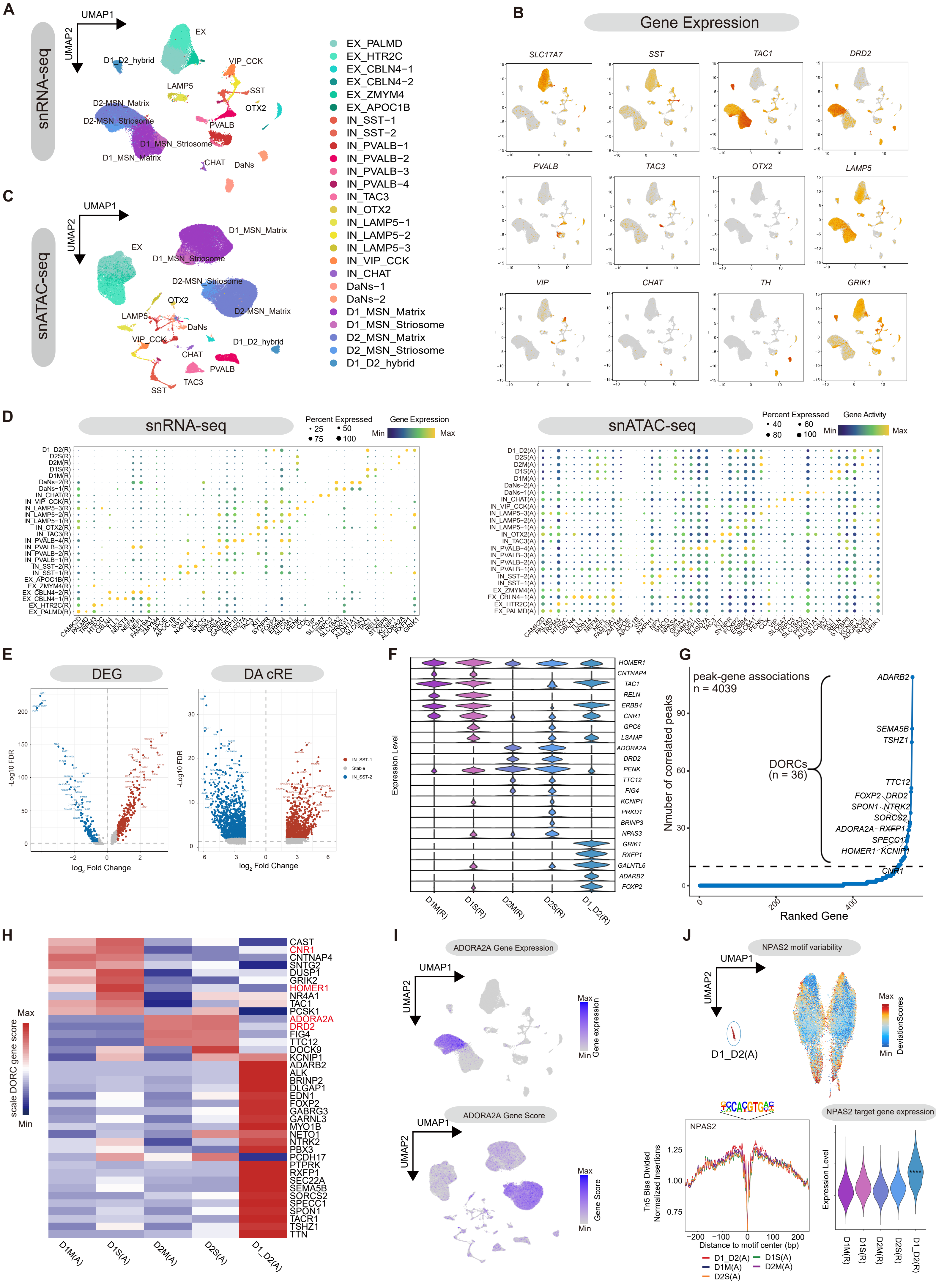

Figure 3

Click here to access/download;Figure;Fig3.pdf

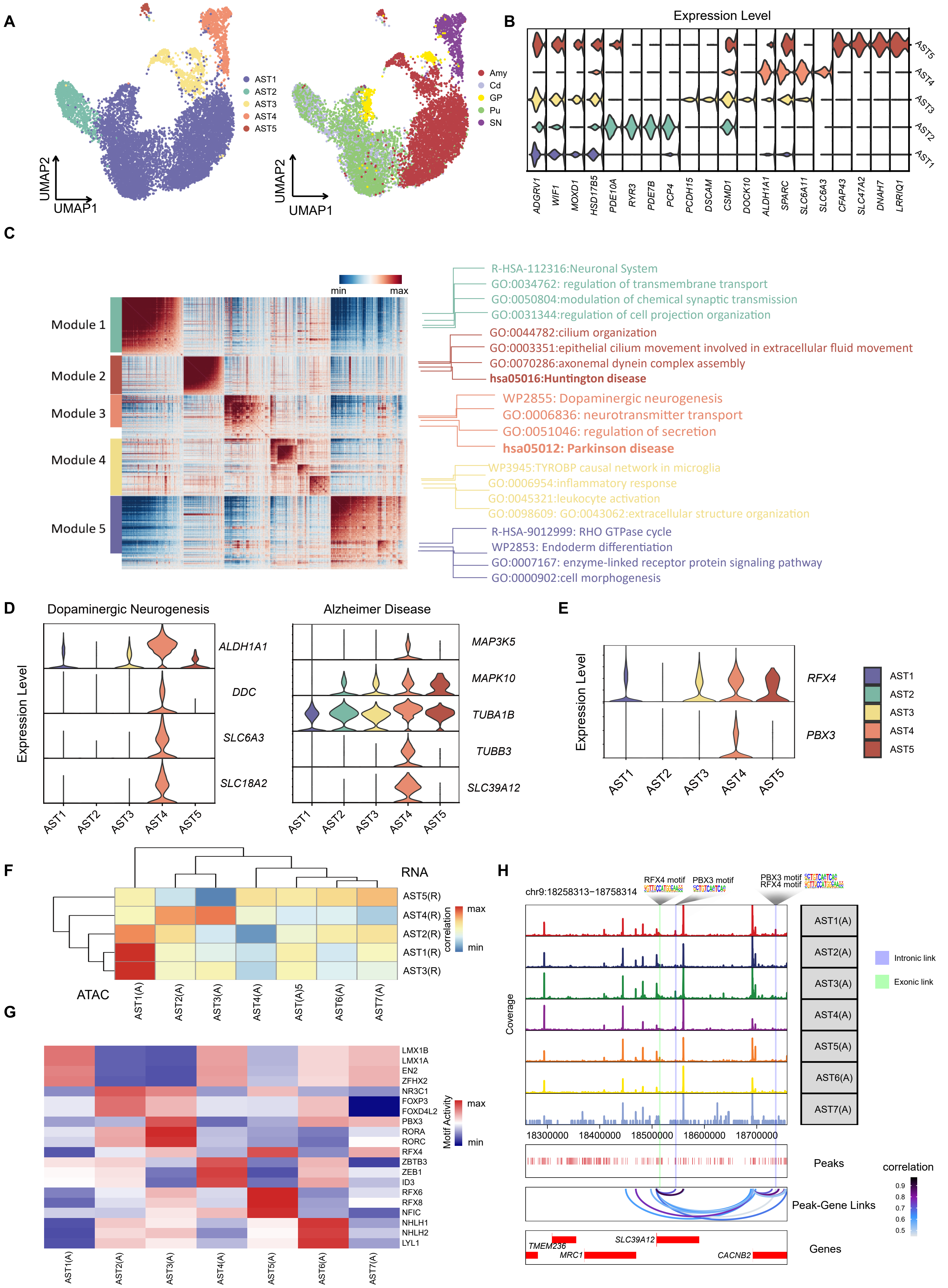

Figure 4

Click here to access/download;Figure;Fig4.pdf

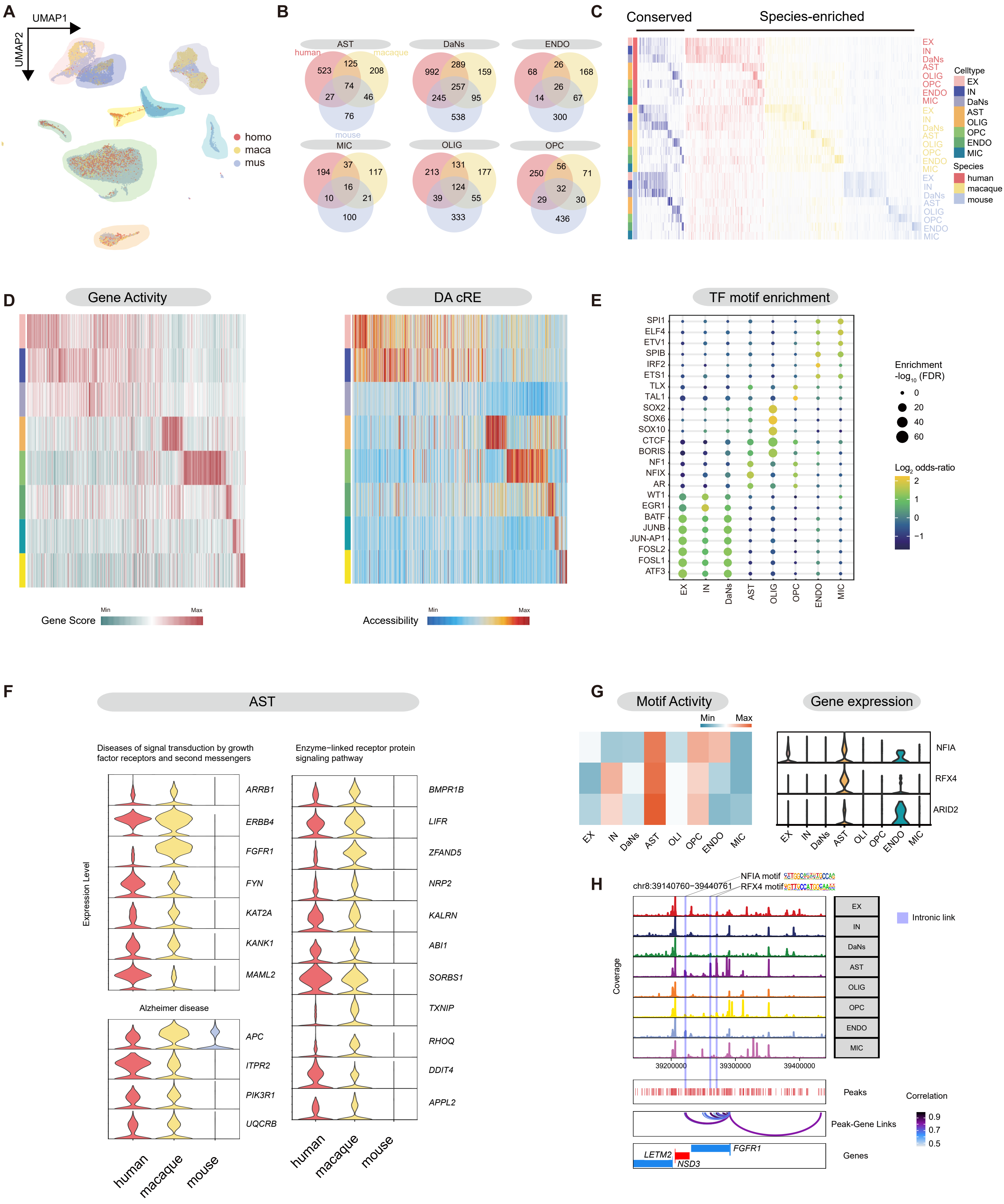

Figure 5

Click here to access/download;Figure;Fig5.pdf

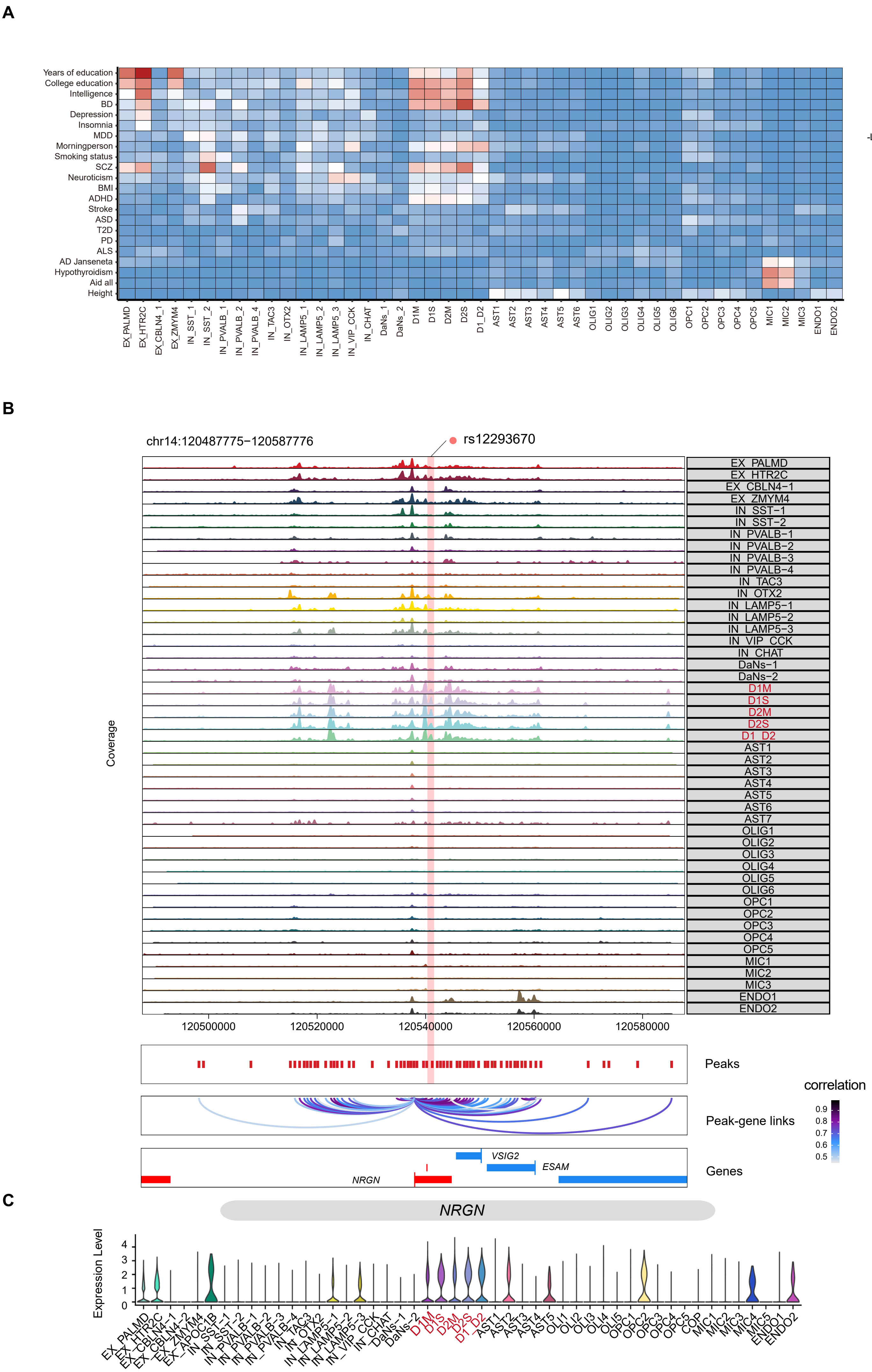

Supplementary Figure S1

Click here to access/download;Figure;FigS1.pdf

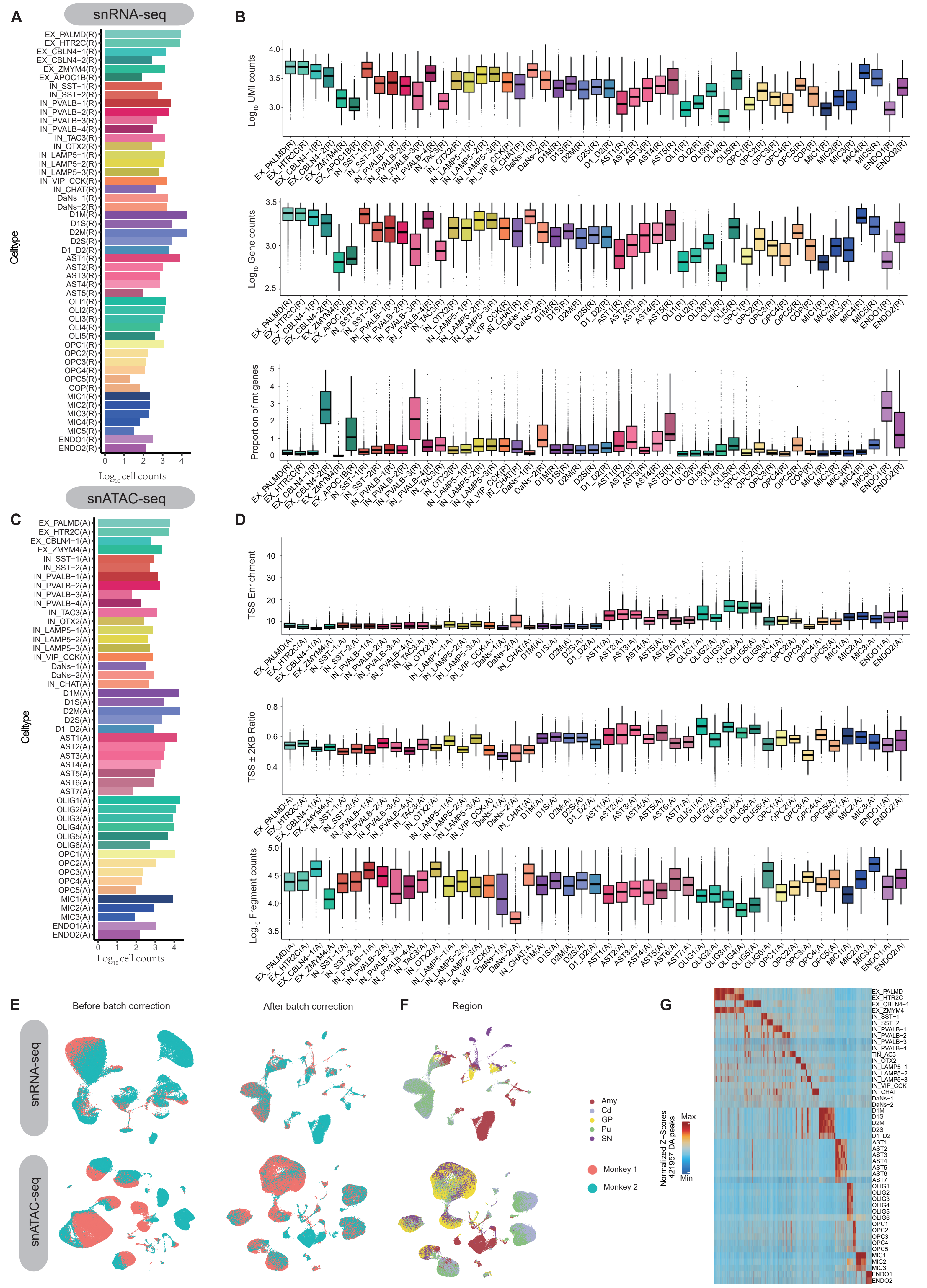

Supplementary Figure S2

Click here to access/download;Figure;FigS2.pdf

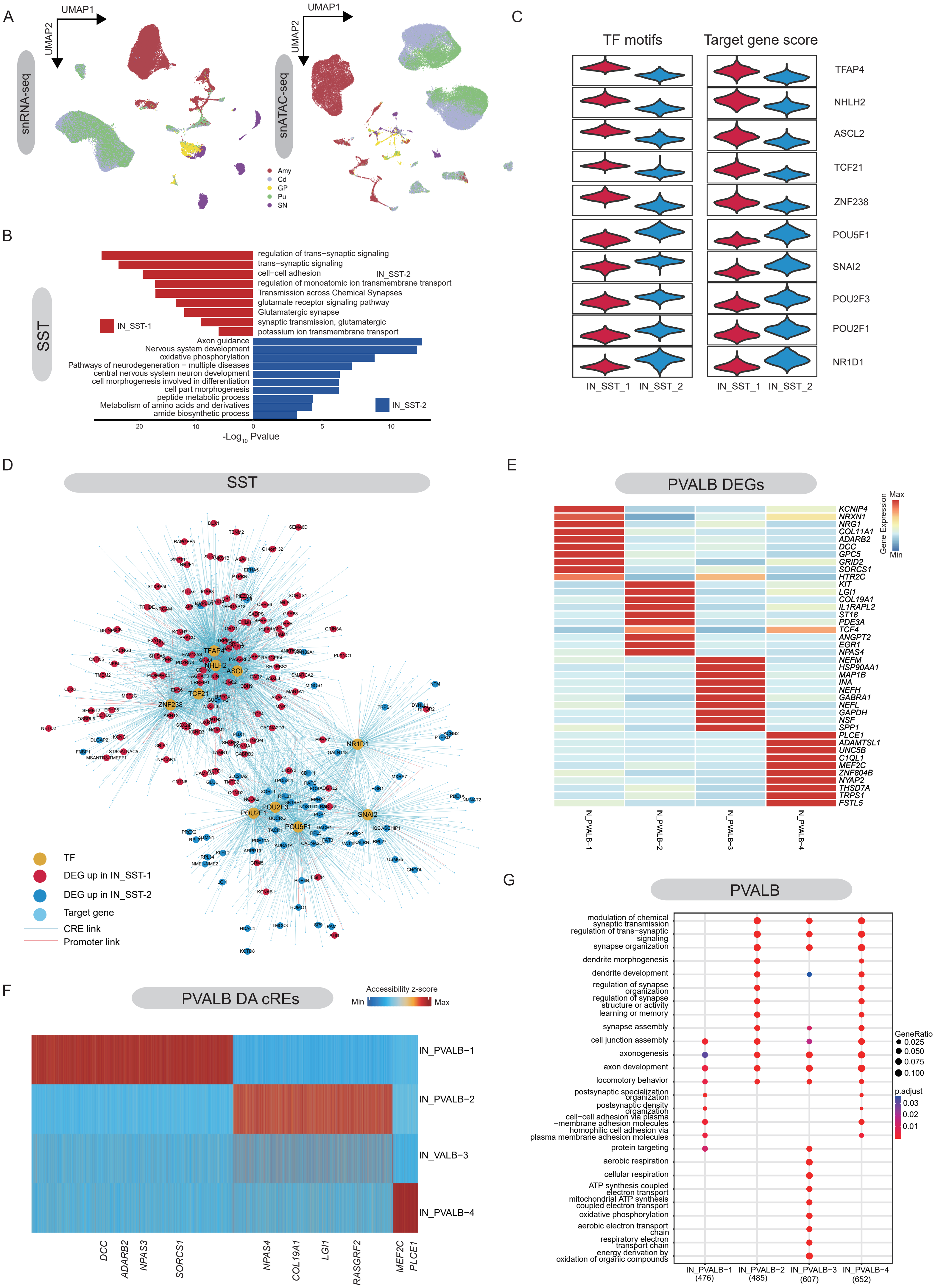

Supplementary Figure S3

Click here to access/download;Figure;FigS3.pdf

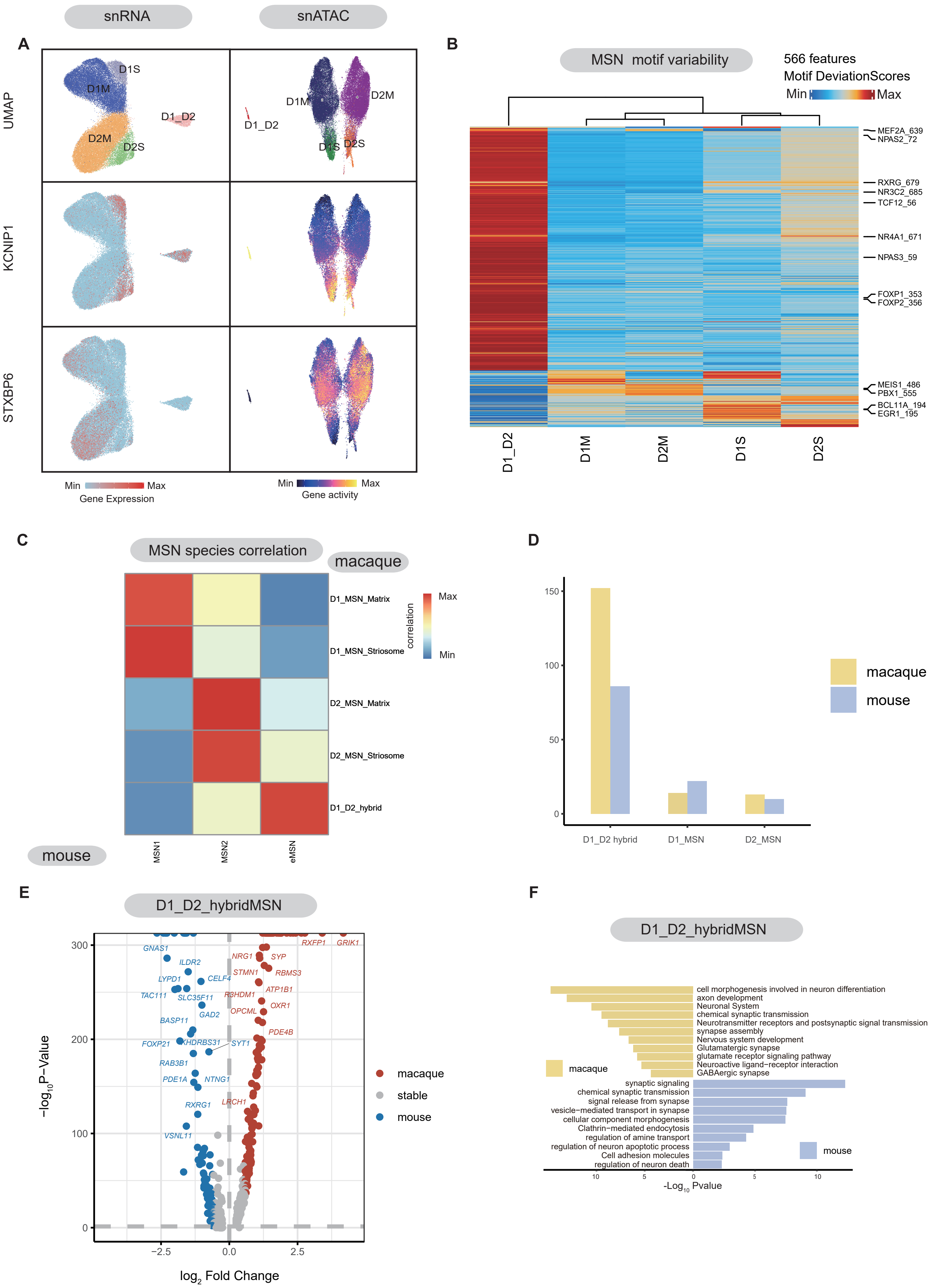

Supplementary Figure S4

Click here to access/download;Figure;FigS4.pdf

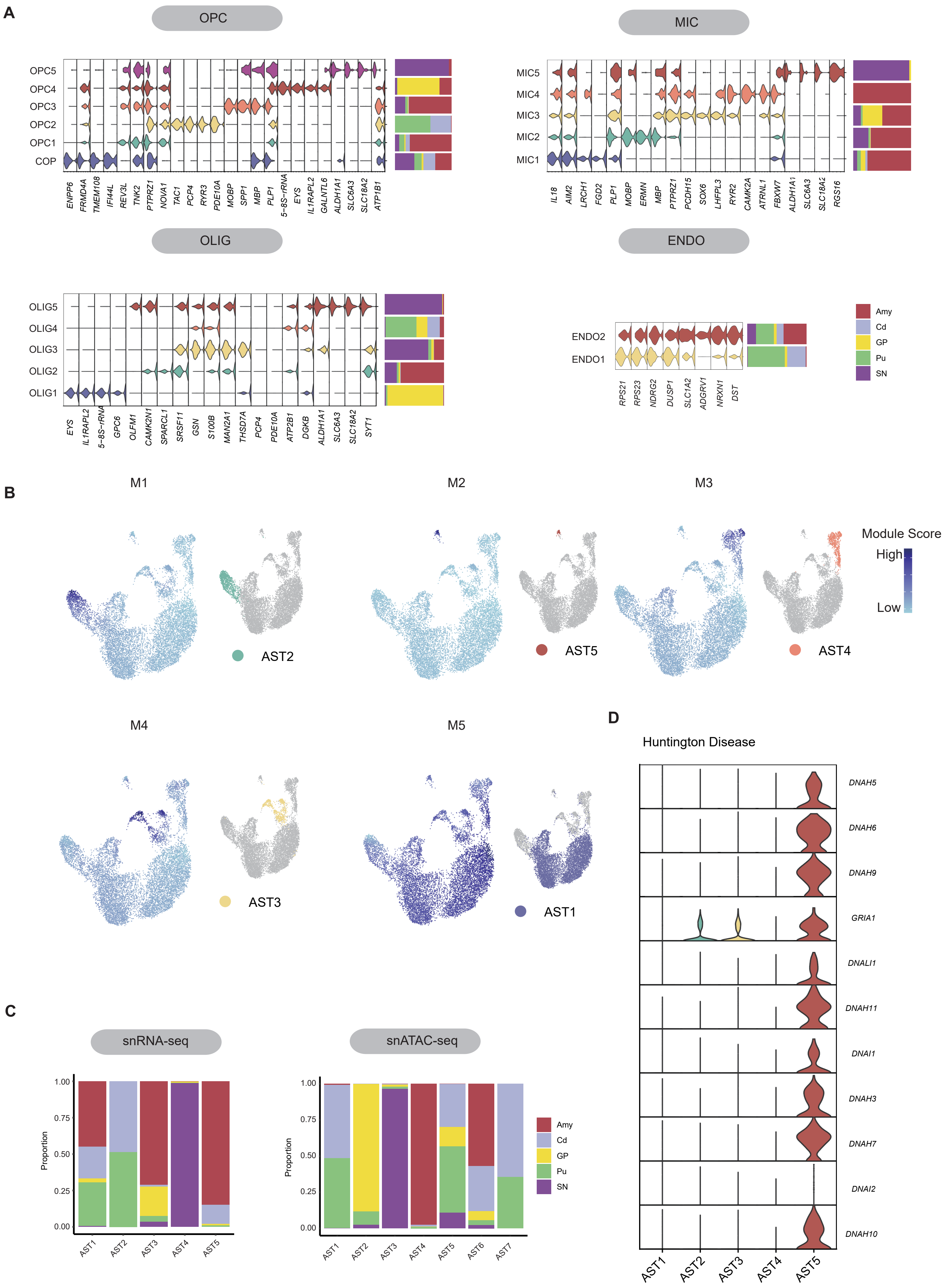

Supplementary Figure S5

Click here to access/download;Figure;FigS5.pdf

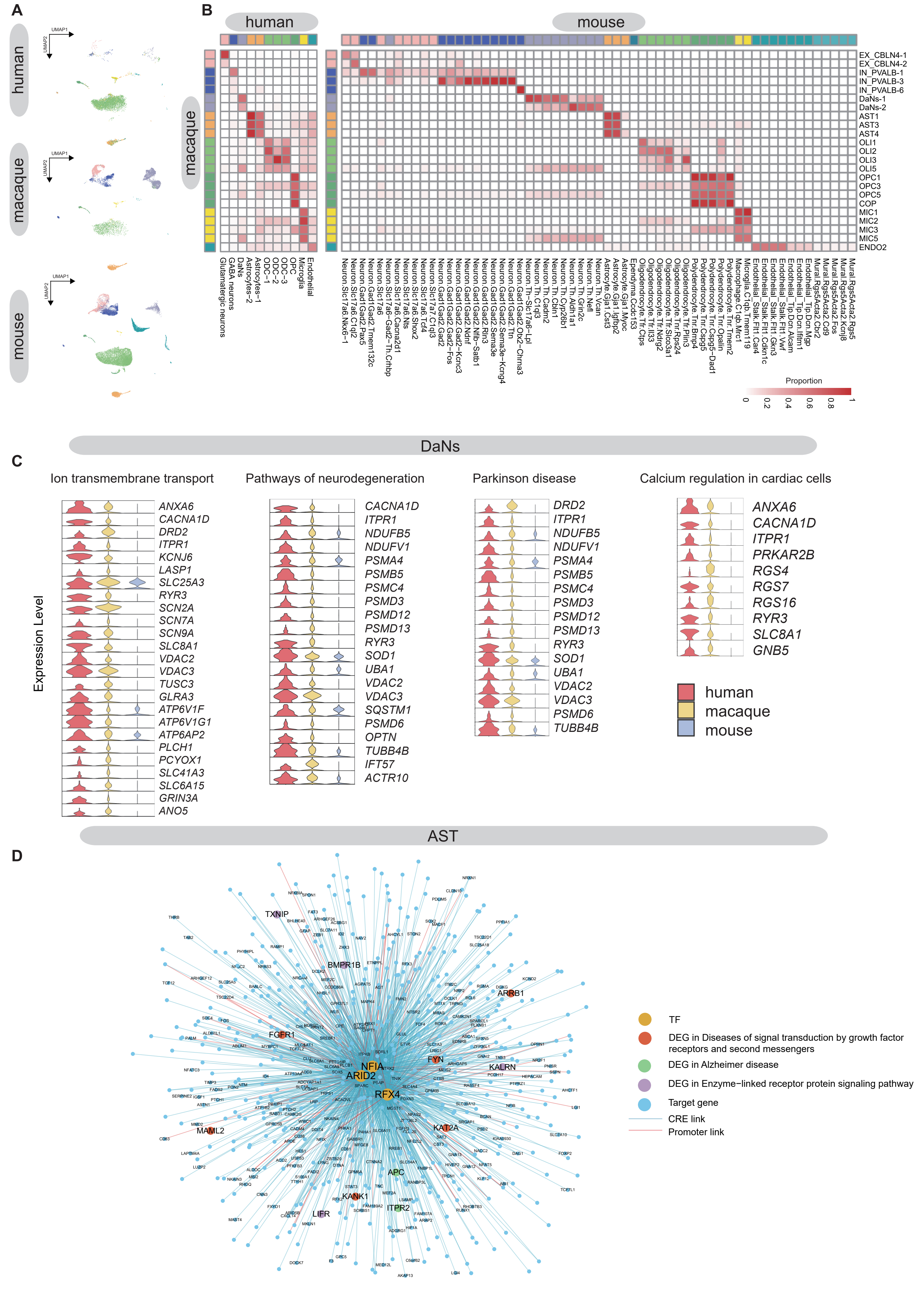

Supplementary Tbale S1

Click here to access/download
Supplementary Material
Supplementary S1.xlsx

Supplementary Tbale S2

Click here to access/download
Supplementary Material
Supplementary S2.xlsx

Click here to access/download
Supplementary Material
Point-by-point response.docx

Personal Cover Click here to access/download;Personal Cover;response Cover Letter.docx

华大生命科学研究院
BGI • research

Aug 08 th , 2023

Dear Hong fang,

Thank you very much for the decision letter regarding our manuscript 'Deciphering the distinct transcriptomic and gene regulatory map in adult macaque basal ganglia cells' (GIGA-D-23-00121) by Sun et al. We were delighted to see that the overall assessment by all three reviewers was positive and that you are interested in the possibility of publishing our work in GigaScience.

The comments by all three experts were excellent and very constructive, and they have been very helpful for us to improve the manuscript. As reviewer #1, we have redefined our cell types based on the definition of cell types from previous studies of the basal ganglia, in order to make our analysis more comprehensive and reliable. As suggested by Reviewer #2, we have explained the differences in major cell types between snATAC and snRNA and their minor impact on the analysis. We have revised the manuscript to fully address the great majority of the reviewers' concerns and provide the point-to-point response to reviewers' comments. All changes to the manuscript have been highlighted in red so that they can be easily tracked. Accordingly, we anticipate that our work will attract broad audience.

We look forward to hearing back from you!

Sincerely,
Ying Lei on behalf of all authors.
