# Supplementary material for: Deciphering the distinct transcriptomic and gene regulatory map in adult macaque basal ganglia cells
Source: Gigascience. 2023 Dec 13;12:giad095. doi: 10.1093/gigascience/giad095 (PMC10716911; doi:10.1093/gigascience/giad095)
Supplement: giad095_Response_to_Reviewer_Comments_Original_Submission [file giad095_response_to_reviewer_comments_original_submission.pdf]

Author's Response To Reviewer Comments

We have compiled our responses to the reviewer and editor comments in the attached Point-by-point response document.
