# Supplementary material for: Deciphering the distinct transcriptomic and gene regulatory map in adult macaque basal ganglia cells
Source: Gigascience. 2023 Dec 13;12:giad095. doi: 10.1093/gigascience/giad095 (PMC10716911; doi:10.1093/gigascience/giad095)
Supplement: giad095_Reviewer_1_Report_Original_Submission — Trygve Bakken -- 6/16/2023 Reviewed [file giad095_reviewer_1_report_original_submission.pdf]

Reviewer Report

Title: Deciphering the distinct transcriptomic and gene regulatory map in adult macaque basal ganglia cells

Version: Original Submission **Date:** 6/16/2023

Reviewer name: Trygve Bakken

Reviewer Comments to Author:

In the manuscript titled "Deciphering the distinct transcriptomic and gene regulatory map in adult macaque basal ganglia cells," the authors present a multi-modal (snRNA and snATAC) cellular atlas of four regions (STR, SN, GP, AMY) from the macaque basal ganglia. Comparative analysis with previous studies in human and mouse identify transcriptomically conserved cell types across species. Integrating epigenetic profiling with disease heritability in humans identifies cell types specifically vulnerable to schizophrenia. This study provides a useful data resource for the field, but it is limited by the lack of integration with state-of-the field cell types that have been published for these regions.Major concerns:1. The authors present a taxonomy of cell types identified through clustering of snRNA-seq profiled in this study and used marker genes to identify major cell types. Several issues arise with how the authors went about determining cell types:* References for marker genes used to identify the major cell types need to be provided in text.* The authors have ignored previous studies of basal ganglia in the primates and rodents which have already expanded the cell type repertoire of the basal ganglia. For example, unreferenced prior studies have identified spatial resolved types including medium spiny neurons that occupy the matrix and striosome compartments of the dorsal basal ganglia. Such studies include:* He et al. 2021 "Transcriptional and anatomical diversity of medium spiny neurons in the primate striatum"* Krienen et al. 2020 "Innovations present in the primate interneuron repertoire"* A.B. MuÃ±oz-Manchado et al. 2018 "Diversity of interneurons in the dorsal striatum revealed by single-cell RNA sequencing and PatchSeq".* Stanley et al. 2022 "Continuous and discrete neuron types of the adult murine striatum"* By not referencing these prior works the authors have duplicated cell typing efforts, in primates, in a manner that is not spatially informed within each region and ignores previous reported cell types which the authors have now redefined and renamed. As an example the authors report on a DRD2-MSN4 type found to differentially express RXFP1. If the authors were to compare with He et al. 2021 the DRD2-MSN4 type would be revealed as the D1/D2-Hybrids that He et al. extensively validated with mFISH. Additionally, the Pvalb6 type appears to be the TAC3+ primate specific interneuron type reported in Krienen et al. 2020.* Given these observations, the reporting of cell types and differential expression cannot be related to current state-of-the-field for primate basal ganglia and the novel aspect of associated results sections are questionable.* If the authors can incorporate the above prior studies then exploring the epigenetic signatures of spatial resolved types from He et al. is an exciting direction for revision.* Incorporation of prior studies could be achieved by learning a mapping between the authors cell types and those reported in each study referenced above.2. The authors identified cell types via independent clustering of both the snRNA-seq and snATAC-seq data. Translation between RNA and ATAC types is identified through label transfer using Seurat. It seems that there is a 1-

1 translation between most, if not all, RNA and ATAC cell types. As such, a common cell type nomenclature across single nucleus modalities would make the manuscript more concise and easier to follow.3. After performing the co-embedding of RNA and ATAC was any regional variation lost in either modality?4. After integration of human, macaque and mouse SN data the authors report "We found that the majority of cell types showed a uniform cross-species distribution". It is unclear what the authors are referring to here, conservation of all cell types across all species?5. From the TF motif analysis the authors identified motifs associated with known transcription factors that appear to also be markers of the identified cell types. The motif analysis is not surprising for the most part and reporting of such analysis should be more concise.6. The higher conservation of TF motifs between primates as compared to primates vs. mouse is expected from previous studies. It would be interesting if the authors report on species specific motifs that are highly enriched in a cell type that derives species specific epigenetic and transcriptional signatures.7. When comparing DA cREs to GWAS summary statistics, were the DA cREs found to be specific to DAs through differential accessibility analysis? Was a cutoff or specific heuristics used to identify DA specific regulatory elements for the LDSC analysis?8. The authors appear to find no MSN subtype specific association of BD-associated loci, was this expected? Could there be a confounding between species or aspects of how MSN subtype specific cREs were identified that lead to no enrichment in specific MSN subtypes?9. The authors state: "Our results proposed medium spiny neurons as potential pathological and therapeutic targets for the future SCZ studies". It would be helpful for the authors to reference previous literature linking MSNs to SCZ.10. To support the authors association of NHP epigenetics to GWAS summary stats in human I would like to see validation that accessibility is conserved in human. BOCA2 from Panos Roussos's lab would provide human validation.11. In the discussion the authors mention providing a comprehensive data resource for NHP disease models which I don't believe the authors have achieved given the limited comparison against previous basal ganglia studies in both primate and rodents.12. Lines 475-478 again discuss the DRD2-MSN4 type related to mouse eMSNs and call for further research which has already been done in He et al. 2021.13. In general the manuscript should be made more concise and focus more on the regional differences that can be identified through the sampling strategy developed by the authors.Minor concerns:* For all dotplot figures (Figure 2D for example), 0 expression should be made blank or no circle shown.* Figure 2B CHAT is hard to see, consider using another color for all panels.* Figure 2G seems blurry?* Dendrogram of Figure 3C could be simplified and reduced.* Motifs reported in Figure 3H are not readable, consider reformatting the panel.

Level of Interest

Please indicate how interesting you found the manuscript: Choose an item.

Quality of Written English

Please indicate the quality of language in the manuscript: Choose an item.

Declaration of Competing Interests

Please complete a declaration of competing interests, considering the following questions:

- Have you in the past five years received reimbursements, fees, funding, or salary from an organisation that may in any way gain or lose financially from the publication of this manuscript, either now or in the future?
- Do you hold any stocks or shares in an organisation that may in any way gain or lose financially from the publication of this manuscript, either now or in the future?
- Do you hold or are you currently applying for any patents relating to the content of the manuscript?
- Have you received reimbursements, fees, funding, or salary from an organization that holds or has applied for patents relating to the content of the manuscript?
- Do you have any other financial competing interests?
- Do you have any non-financial competing interests in relation to this paper?

If you can answer no to all of the above, write 'I declare that I have no competing interests' below. If your reply is yes to any, please give details below.

I declare that I have no competing interests.

I agree to the open peer review policy of the journal. I understand that my name will be included on my report to the authors and, if the manuscript is accepted for publication, my named report including any attachments I upload will be posted on the website along with the authors' responses. I agree for my report to be made available under an Open Access Creative Commons CC-BY license (http://creativecommons.org/licenses/by/4.0/). I understand that any comments which I do not wish to be included in my named report can be included as confidential comments to the editors, which will not be published.

Choose an item.

To further support our reviewers, we have joined with Publons, where you can gain additional credit to further highlight your hard work (see: https://publons.com/journal/530/gigascience). On publication of this paper, your review will be automatically added to Publons, you can then choose whether or not to claim your Publons credit. I understand this statement.

Yes Choose an item.
