# Supplementary material for: Deciphering the distinct transcriptomic and gene regulatory map in adult macaque basal ganglia cells
Source: Gigascience. 2023 Dec 13;12:giad095. doi: 10.1093/gigascience/giad095 (PMC10716911; doi:10.1093/gigascience/giad095)
Supplement: giad095_Reviewer_2_Report_Original_Submission — Yiqiao Wang -- 7/5/2023 Reviewed [file giad095_reviewer_2_report_original_submission.pdf]

Reviewer Report

Title: Deciphering the distinct transcriptomic and gene regulatory map in adult macaque basal ganglia cells

Version: Original Submission **Date:** 7/5/2023

Reviewer name: Yiqiao Wang

Reviewer Comments to Author:

The study titled " Deciphering the distinct transcriptomic and gene regulatory map in adult macaque basal ganglia cells" presents a detailed investigation into the cellular composition and molecular regulation of the macaque basal ganglia using state-of-the-art transcriptomic and epigenomic techniques. The study addresses an important knowledge gap in our understanding of the basal ganglia and its relevance to neurological disorders.One of the strengths of this study is the integration of multiple-omics datasets, including single-nucleus RNA sequencing (snRNA-seq) and single-nucleus Assay for Transposase-Accessible Chromatin sequencing (snATAC-seq). The conversion of chromatin accessibility data to gene activity data further strengthens the robustness of their findings. The study's methodology is well-described, and the data analysis approaches are appropriate and well-justified. The integration of transcriptomics with epigenomics, as well as the linkage of GWAS loci to cell types, demonstrates the authors' comprehensive approach to unraveling the regulatory mechanisms underlying the macaque basal ganglia. The inclusion of multiple species (humans, macaques, and mice) adds to the significance and generalizability of the findings.While the study presents a wealth of valuable information, there are a few areas that could benefit from further discussion or clarification. The authors could elaborate on the potential functional implications of the identified heterogeneity within neuronal subtypes and provide more context for the observed differences between species. Additionally, the potential limitations of the study, such as technical constraints or sample size considerations, could be acknowledged to provide a balanced assessment of the findings.Specific Comments:ï□¬ Figures 1A and 1B (Supplementary Figures 1A and 1C)The clusters of the major cell types are quite different between snATAC-seq and snRNA-seq. To what extent this might affect the quality of the results?ï□¬ Figure 2E1. The annotation of some genes with significant differences lacks clarity (perhaps need more lines to connect the names and dots).2. It is strange that there is a big gap between log2 Fold Change -2 to 2 in the DA cRE panel.ï□¬ Figure 3AThe cluster of AST1 doesn't look good. Many cells are dispersed in the clusters of AST3 and AST4.ï□¬ Figure 4GThe values of the Min and Max are missed.ï□¬ Supplementary Figure 2FThe GeneRatio is only labeled with two values, 0.05 and 0.1, but the range of GeneRatio in the figure is larger. It is recommended to add 1-2 additional values for labeling.Minor Suggestions:1. Include a brief introduction to contextualize the study and highlight the potential applications and implications of investigating gene expression and regulatory elements in different cell types, such as their relevance to precision medicine, therapeutic targeting, and understanding developmental processes.2. Provide more context regarding the potential limitations of the study, including potential sources of bias, the generalizability of the findings to other tissues or species, and areas for future research. This would help the readers better understand the scope and

potential boundaries of the study.Overall, the authors have successfully addressed their research objectives and provided a comprehensive analysis of the macaque basal ganglia at the transcriptomic and epigenomic levels. The integration of various omics data, the identification of cell type heterogeneity, and the exploration of regulatory mechanisms enhance our understanding of the basal ganglia and its relevance to neurological disorders. With the suggested minor revisions and additions, the manuscript would be ready for publication.

Level of Interest

Please indicate how interesting you found the manuscript: Choose an item.

Quality of Written English

Please indicate the quality of language in the manuscript: Choose an item.

Declaration of Competing Interests

Please complete a declaration of competing interests, considering the following questions:

- Have you in the past five years received reimbursements, fees, funding, or salary from an organisation that may in any way gain or lose financially from the publication of this manuscript, either now or in the future?
- Do you hold any stocks or shares in an organisation that may in any way gain or lose financially from the publication of this manuscript, either now or in the future?
- Do you hold or are you currently applying for any patents relating to the content of the manuscript?
- Have you received reimbursements, fees, funding, or salary from an organization that holds or has applied for patents relating to the content of the manuscript?
- Do you have any other financial competing interests?
- Do you have any non-financial competing interests in relation to this paper?

If you can answer no to all of the above, write 'I declare that I have no competing interests' below. If your reply is yes to any, please give details below.

I declare that I have no competing interests.

I agree to the open peer review policy of the journal. I understand that my name will be included on my report to the authors and, if the manuscript is accepted for publication, my named report including any attachments I upload will be posted on the website along with the authors' responses. I agree for my report to be made available under an Open Access Creative Commons CC-BY license (http://creativecommons.org/licenses/by/4.0/). I understand that any comments which I do not wish to

be included in my named report can be included as confidential comments to the editors, which will not be published.

Choose an item.

To further support our reviewers, we have joined with Publons, where you can gain additional credit to further highlight your hard work (see: https://publons.com/journal/530/gigascience). On publication of this paper, your review will be automatically added to Publons, you can then choose whether or not to claim your Publons credit. I understand this statement.

Yes Choose an item.
